# Exploring correlations between gut mycobiome and lymphocytes in melanoma patients undergoing anti-PD-1 therapy

**DOI:** 10.1007/s00262-024-03918-9

**Published:** 2025-02-25

**Authors:** Natalia Szóstak, Michał Budnik, Katarzyna Tomela, Luiza Handschuh, Anna Samelak-Czajka, Bernadeta Pietrzak, Marcin Schmidt, Mariusz Kaczmarek, Łukasz Galus, Jacek Mackiewicz, Andrzej Mackiewicz, Piotr Kozlowski, Anna Philips

**Affiliations:** 1 Institute of Bioorganic Chemistry, Polish Academy of Sciences, Poznan, Poland; 2 Department of Cancer Immunology, Chair of Medical Biotechnology, Poznan University of Medical Sciences, Poznan, Poland; 3 Department of Diagnostics and Cancer Immunology, Greater Poland Cancer Centre, 61-866 Poznan, Poland; 4 Department of Food Biotechnology and Microbiology, Poznan University of Life Sciences, Poznan, Poland; 5 Department of Medical and Experimental Oncology, Institute of Oncology, Poznan University of Medical Sciences, Poznan, Poland

**Keywords:** Melanoma, Gut mycobiome, Immune response, Lymphocytes, Anti-PD-1

## Abstract

Research has shown that the microbiome can influence how the immune system responds to melanoma cells, affecting the course of the disease and the outcome of the therapy. Here, we used the metagenomic approach and flow cytometry analyses of blood cells to discover correlations between gut fungi of metastatic melanoma patients enrolled in anti-PD-1 therapy and lymphocytes in their blood.

We analyzed the patterns of associations before the first administration of anti-PD-1 therapy (BT, n = 61) and in the third month of the therapy (T3, n = 37), allowing us to track changes during treatment. To understand the possible impact of gut fungi on the efficacy of anti-PD-1 therapy, we analyzed the associations in clinical beneficiaries (CB, n = 37) and non-beneficiaries (NB, n = 24), as well as responders (R, n = 28) and non-responders (NR, n = 33).

Patients with LDH < 338 units/L, overall survival (OS) > 12, CB, as well as R, had lower levels of Shannon diversity (*p* = 0.02, *p* = 0.05, *p* = 0.05, and *p* = 0.03, respectively). We found that the correlation pattern between intestinal fungi and lymphocytes was specific to the type of response, positive or negative. When comparing CB and NB groups, correlations with opposite directions were detected for *C. albicans*, suggesting a response-specific immune reaction. For CB, *M. restricta* exhibited a set of correlations with different types of lymphocytes, with prevalent positive correlations, suggesting a robust immune response in the CB group. This result extends our former research, where *M. restricta* and *C. albicans* were associated with an increased risk of melanoma progression and a poorer response to anti-PD-1 treatment.

**Supplementary Information:**

The online version contains supplementary material available at 10.1007/s00262-024-03918-9.

## Introduction

The emergence of immuno-oncology and the integration of anti-PD-1 antibodies, such as nivolumab and pembrolizumab, into mainstream clinical practice have substantially enhanced the outlook for advanced melanoma patients. Notwithstanding the considerable advancements in the prognosis of individuals with metastatic melanoma, around 50–70% of patients receiving PD-1 inhibitors alone or in combination with anti-CTLA4 or anti-LAG3 did not exhibit a positive response to the treatment. Additionally, one-third of patients who displayed initial response developed resistance to the drugs, leading to melanoma progression within a span of three years [1]. Furthermore, among melanoma patients undergoing immune checkpoint inhibitors (ICIs) treatment, most of them develop immune-related adverse events (irAEs), with the incidence of serious grade (grade 3–5) rising to more than 50% when nivolumab is administered with ipilimumab (anti-CTLA4) [2].

In this light, recent research has pointed to the gut microbiome as a contributing factor in melanoma progression and immunotherapy effectiveness [3]. Alterations in microbiome diversity have been documented in melanoma patients [4], showing that the microbiome can impact melanoma development by influencing host cell metabolism. Studies have demonstrated the microbiome’s ability to modulate the immune response to melanoma cells, thereby affecting disease progression and ICI treatment outcome, including the severity of irAEs [5, 6].

So far, research on gut microbiota has been primarily focused on bacteria, and only recently, attention has also turned to fungi residing in the intestine as important contributors to melanoma progression. Very little is known about gut fungi and their role in homeostasis, not to mention the details concerning the interplay between specific immune pathways and particular fungi species. Thus far, only several gut fungal species have been more extensively studied in these terms, including *Saccharomyces cerevisiae* [7], *Aspergillus fumigatus*, and species from the *Candida* genus, with *Candida albicans* upfront [8]. Their role in the immune response during cancer treatment is even more elusive. Still, those hints should not be overlooked, as some studies show the possible connections between mycobiota and cancer progression, treatment efficiency, and patient prognosis.

The influence of fungi is linked to the production of various metabolites that strongly affect metabolic pathways [9]. Additionally, the fungal cell wall components, including chitin, β-glucan, and mannan, can interact with the host immune system, inducing pro-inflammatory responses [10, 11]. Considering inflammation as a key contributor to cancer development [12, 13], intestinal inflammation associated with altered microbial profiles may contribute to maintaining a systemic pro-inflammatory and potentially pro-oncogenic state [14, 15], thereby influencing immunotherapy effectiveness.

In our last paper, we demonstrated that patients with malignant melanoma exhibited a distinct structure of gut mycobiota compared to healthy individuals [16]. Potentially harmful fungal species, namely *C. albicans*, *Candida dubliniensis*, and *Neurospora crassa,* were more abundant in oncological patients, while considered beneficial *S. cerevisiae* and *Debaryomyces hansenii* were found in lower amounts. We also showed that during anti-PD-1 treatment, the relative amount of *Malassezia restricta* and *C. albicans* increased. We demonstrated that patients who did not experience clinical benefits from ICI therapy had higher richness and diversity of gut fungi than patients who did benefit clinically. Also, an elevated abundance of *Saccharomyces paradoxus* correlated with a favorable response to anti-PD-1 treatment, whereas a heightened presence of *Tetrapisispora blattae* was linked to an absence of clinical benefits. We revealed that high levels of potentially harmful fungal species, namely *M. restricta* and *C. albicans,* were associated with an increased risk of melanoma progression and a poorer response to anti-PD-1 treatment.

Although we have already revealed some links between intestinal fungi, melanoma progression, and the efficacy of anti-PD-1 therapy, the exact mechanism is far from understood. Meanwhile, understanding the dynamics between intestinal fungi and the immune response may be crucial to the success of immunotherapy. In this study, we employed high-throughput sequencing and liquid chromatography-mass spectrometry analysis to explore the correlations between gut fungal microbiota and lymphocytes circulating in the blood of individuals undergoing anti-PD-1 therapy for malignant melanoma. Our research endeavors to unravel the nuanced correlations within this intricate system, shedding light on potential avenues for therapeutic optimization and personalized patient care.

## Materials and methods

### Ethics approval and consent to participate

This study involved human participants and was approved by the Bioethical Commission of the Karol Marcinkowski University of Medical Sciences, Poznan, Poland (resolution No 316/22, passed on 14 April 2022, as an extension of resolution No. 485/19, passed on 11 April 2019). The study was conducted in accordance with the Declaration of Helsinki. Participants gave written informed consent to collect stool samples and study information before taking part. All methods were carried out in accordance with relevant guidelines and regulations.

Patients or the public were not involved in the design, conduct, reporting, or dissemination of the research.

### Cohort description and study participants

Stool samples were collected from 61 metastatic melanoma patients selected from the Polish Microbiome Map project (ClinicalTrials.gov study identifier: NCT04169867). All patients had histologically confirmed unresectable stage III or stage IV cutaneous melanoma and were enrolled in treatment with anti-PD-1 therapy (nivolumab or pembrolizumab) as a part of the Ministry of Health (Poland) drug program [17]. The dosage for patients treated with nivolumab was 480 mg infused in cycles every four weeks, and for patients treated with pembrolizumab, it was 400 mg in cycles every six weeks. Samples of peripheral blood and stool samples were obtained from patients at two time points: at the initiation of therapy (BT, n = 61) and in the third month of treatment (T3, n = 37). T3 patients treated with nivolumab received three cycles of treatment, and patients treated with pembrolizumab had two cycles. Patients with infectious diseases were excluded from this study.

All patients were recruited to the study at the Department of Medical and Experimental Oncology, Heliodor Swiecicki Clinical Hospital, Poznan University of Medical Sciences (Poznan, Poland), from June 2018 to December 2021.

Clinical information, including tumor stage and serum lactate dehydrogenase (LDH) concentration, was collected from the medical records. Response to anti-PD-1 therapy was assessed according to the response evaluation criteria in solid tumors (RECIST) v.1.1 criteria in the third month of treatment. Responders (R, n = 27) were defined as patients with complete (CR) or partial (PR) responses, while non-responders (NR, n = 34) were defined as patients with stable disease (SD) or progression (PD). An additional, independent classification categorized patients into those who exhibited clinical benefit (CB, n = 37) from ICI therapy (CR, PR, and SD) and those who did not (NB, n = 24, consisting of PD). Patients’ data is available in Table S1.

### Lymphocyte separation and cryopreservation

Peripheral blood mononuclear cells (PBMC) were isolated within three hours of donation from blood collected in anticoagulant (heparin) tubes. Separated by gradient centrifugation in a Histopaque 1077 (#SD10771B, Sigma-Aldrich, St. Louis, MO, USA), cells were washed in phosphate-buffered saline (PBS) solution, cryopreserved using the CTL-Cryo™ ABC Media Kit (#CTLC-ABC, CTL, Cleveland, OH, USA) and stored in the vapor phase of liquid nitrogen. Samples were frozen for a maximum duration of three years. Thawed and washed twice in PBS, the PBMC pellet was used for flow cytometry analyses.

### Flow cytometry for lymphocytes and subsets

Cell immunophenotyping was performed by flow cytometry. In several designed experiments, cell staining was performed using up to six labeled monoclonal antibodies (mAbs) for each blood sample. A detailed list of mAbs is provided in Table S2.

PBMC pellet was resuspended in 100 µl PBS, stained with mAbs, incubated for 15 min, and protected from light. Next, 500 µL of cell lysis solution (#349,202, BD Biosciences, Franklin Lakes, NJ, USA) was added due to cell fixation properties and stabilization of cell-antibody interaction, and the tubes were incubated for 10 min. After washing with PBS, samples were acquired using a FACS Aria flow cytometer (BD Biosciences, Franklin Lakes, NJ, USA). The obtained results were analyzed with the FACS Diva software v. 6.1.3 (Becton Dickinson, Franklin Lakes, NJ, USA), integrated with the cytometer. The percentage of positive cells and mean fluorescence intensity (MFI) were determined for each examined antibody.

Definitions of lymphocytes used in this study are available in Table S3.

### Metagenomic sequencing

Sampling, DNA isolation, sequencing libraries preparation, and whole genome high throughput sequencing were performed according to the protocol described in our previous studies [18, 19]. The details are provided below.

### Sampling and storage of volunteer samples

Fecal samples (approx. 1 g) were self-collected by all donors into vials containing 3 ml of RNAlater Stabilization Solution (Invitrogen, ThermoFisher Scientific) and delivered within 24 h to the laboratory, where the samples were anonymized and stored at 4 °C for up to one week. Each sample was homogenized. Tubes were centrifuged at 14,000 g for 5 min, the supernatant was discarded, and residues were transferred to a − 20 °C freezer for storage until DNA extraction (usually a few weeks).

### DNA isolation

The frozen stool samples were thawed on ice, and DNA was extracted from them using a DNAeasy PowerSoil Pro Kit (Qiagen, Germany) according to the manufacturer’s instructions. The following protocol adjustments were applied. The liquid phase of stabilized stool samples was thoroughly discarded to remove high salt content that may interfere with a subsequent DNA purification step. Next, the stabilized stool samples (250 mg) were bead-beaten in PowerBead Pro tubes (Qiagen, cat. no. 19301) containing a mix of zirconium beads of different diameters using a Mixer Mill MM400 (Retsch, Germany) for 15 min at 25 Hz. To remove RNA and increase DNA yield, each sample was incubated with 5 µL RNase (10 mg/mL concentration; A&A Biotechnology, Poland) at 60 °C for 10 min. The DNA quality was verified with agarose gel electrophoresis. The final DNA concentration was measured by a Nanodrop ND-1000 spectrophotometer (Thermo Fisher Scientific, USA).

### DNA library preparation

Libraries were constructed with the TruePrep DNA library prep kit V2 for Illumina TD501 (Vazyme Biotech, China) according to the manufacturer’s protocol. 500 ng of stool-extracted DNA was used for each library preparation. In the library amplification step, six PCR cycles were applied. Library concentration was measured using a Qubit fluorometer and Qubit DNA HS Assay Kit (Thermo Fisher Scientific, USA). The quality of libraries and fragment distribution were analyzed using a Bioanalyzer 2100 and DNA 1000 Kit or High Sensitivity DNA Kit (Agilent Technologies, USA), depending on the obtained library quantity. Purified libraries were stored for a few weeks at − 20 °C until sequencing.

### High throughput whole genome sequencing

Before sequencing, all libraries were thawed on ice and normalized to a final concentration of 10 nM. Libraries with distinctive index combinations were pooled together and diluted with EB Buffer (Qiagen, Germany) to obtain a mix of 2 nM libraries, according to Protocol A: Standard Normalization Method for the NextSeq system (Illumina, USA). Whole genome sequencing was performed with NextSeq 550 (Illumina, USA) using High Output Kit v2.5 reagents (Illumina, USA); approximately 10 million 150 bp paired-end reads were generated per library. Neither human DNA sequence depletion nor microbial or viral DNA enrichment was performed.

### Data preprocessing and quality control

Demultiplexing was run on the raw BCL intensity file with the bcl2fastq tool [20] for base calling and separating the reads from different samples. To assess the quality of the sequencing procedure, we generated quality control reports with FastQC [21] and MultiQC [22]. We preprocessed the raw fastq reads with cutadapt [23] using the following procedure: we trimmed the adapter sequences (based on TruSeq adapter sequences) and poly-G tails observed in the data characteristic of the two-channel sequencing technology of NextSeq. We also filtered out reads shorter than 140 bases to remove the bias in taxonomy profiling that could emerge from the shorter sequences. The remaining reads were subjected to further analysis.

### Fungal community profiling

The fungal microbiome was profiled via Kraken2 [24, 25], followed by Bracken [26].

### Statistical analysis

Statistical analysis of the data was performed in R language environment v. 4.1.1. The Kruskal–Wallis test (for continuous data), Chi-square test, and Fisher’s exact test (for categorical data) were used to study whether there were significant differences between analyzed groups (R vs. NR, CB vs. NB) in terms of variables, accepting a confidence level of 95%. Data were log-ratio centered to analyze the structure of mycobial communities across samples (β-diversity, clr transformation, clr function, compositions R package). A pseudocount of 0.005 was added to the species raw count prior to distance calculation (Euclidean distance) to avoid zeros, as clr transformation cannot be used with non-positive data. For β-diversity significance testing, Permutational Multivariate analysis of Variance Using Distance Matrices was conducted (adonis [27], Euclidean distance, vegan R package, 999 permutations), accompanied by the Wilcoxon rank-sum test with continuity correction (pairwise.wilcox.test function, stats R package, p.adjust BH method). The principal component analysis (PCA, R package stats) was employed to illustrate how the community structure of individual samples across the study population differs. Pearson correlation was used to study the associations between the fungal abundance and lymphocyte counts, as well as patients’ characteristics and lymphocyte counts. For analysis of the correlation between the change in counts of each analyzed fungal species (BT vs. T3) and the change in the number of each analyzed lymphocyte type (BT vs. T3), paired samples correlation was performed with the use of the Pearson method, *p* < 0.05. The difference in counts for each fungal species was obtained by subtracting the fungal species count before the treatment (BT) from the count in the third month of the treatment (T3) for all matched samples. Similarly, the change in the number of lymphocytes was obtained by subtracting the number of a given lymphocyte type before the treatment (BT) from the number in the third month of the treatment (T3) for all matched samples.

#### Data availability

Data generated in this study are available within the article and its Supplementary Data Files or from the corresponding author upon reasonable request. The raw NGS data is available in the NCBI SRA repository (https://www.ncbi.nlm.nih.gov/sra) under accession number PRJNA972625.

## Results

### Characteristics of the study cohort

Sixty-one patients with unresectable stage III or stage IV cutaneous melanoma treated with anti-PD-1 inhibitors were recruited for the study. Stool and blood samples from the enrolled melanoma patients were analyzed at two time points: before treatment (BT) and after three months of treatment (T3) (61 and 37 samples, respectively). In total, we obtained 98 samples from melanoma patients. Among the melanoma patients treated with the anti-PD-1, 37 patients exhibited clinical benefits (CB) from ICI therapy, and 24 did not (NB); of these, 28 patients responded to therapy (R), while 33 did not (NR). The baseline clinical data and characteristics of patients and controls are shown in Table 1. The mean age of melanoma participants was 64 ± 13 years; the youngest patient was 32, and the oldest was 92. Twenty-three (37.7%) of the enrolled participants were female and thirty-eight (62.3%) were male. Over 95% of patients were diagnosed at the IV stage, and only three of them (4.92%) were diagnosed at the III stage of the disease. The type of therapy was decided by the patient’s oncologist: 33 (54.10%) of the patients were treated with nivolumab, and the other 28 received pembrolizumab (45.90%). For 52 patients (85.2%), it was the first line of treatment; for 9 (14.8%), it was the second.

**Table 1:** Demographic and clinical data for advanced melanoma patients included in the study

Patients Characteristics	Responders (R, n = 28)	Non-responders (NR, n = 33)	*p* (R ~ NR)	Clinical beneficiaries (CB, n = 37)	Nonbeneficiaries (NB, n = 24)	*p* (CB ~ NB)
Sex, *n* (%)						
Male	14 (50.00)	24 (72.73)	0.12^a^	21 (56.76)	17 (70.83)	0.40^a^
Female	14 (50.00)	9 (27.27)	0.11^b^	16 (43.24)	7 (29.17)	0.29^b^
Age (years)						
Median	63.50	69.00	0.11^c^	64	70	0.17^c^
Mean	61.64	67.36		62.76	67.79	
Min–max	32–84	38–92		32–85	38–92	
SD	13.15	12.63		13.32	12.36	
Serum LDH (U/L)						
Median	204	232	0.08^c^	194	285	**0.01**^c^
Mean	219.14	339.79		213.84	393.21	
Min–max	121–474	141–1173		121–474	141–1173	
SD	71.22	250.47		64.75	275.50	
M-stage at diagnosis, *n* (%)						
IV M1a	8 (28.57)	7 (21.21)	0.30^b^	13 (35.14)	2 (8.33)	**0.03**^b^
IV M1b	4 (14.29)	5 (15.15)	0.35^c^	6 (16.22)	3 (12.50)	**0.02**^c^
IV M1c	9 (32.14)	13 (39.39)		11 (29.73)	11 (45.83)	
IV M1d	4 (14.29)	8 (24.24)		4 (10.81)	8 (33.33)	
III M1c	3 (10.71)			3 (8.11)		
BRAF mutation status, *n* (%)						
BRAF −	18 (64.29)	18 (54.55)	0.61^a^	24 (64.86)	12 (50.0)	0.38^a^
BRAF +	10 (35.71)	15 (45.45)	0.67^b^	13 (35.14)	12 (50.0)	0.29^b^
Immunotherapy *n* (%)						
Nivolumab	14 (50.00)	19 (57.58)	0.74^a^	20 (54.05)	13 (54.17)	1^a^
Pembrolizumab	14 (50.00)	14 (42.42)	0.61^b^	17 (45.95)	11 (45.83)	1^b^
Line of treatment, *n* (%)						
I	24 (85.71)	28 (84.85)	1^a^	31 (83.78)	21 (87.5)	0.98^a^
II	4 (1.29)	5 (15.15)	1^b^	6 (16.22)	3 (12.5)	1^b^
Best overall response, *n* (%)						
Partial response (PR)	28 (100.00)		** < 2.2e-16**^**a**^	28 (75.68)		** < 2.2e-16**^**a**^
Stable disease (SD)		9 (27.27)	** < 2.2e-16**^**b**^	9 (24.32)		** < 2.2e-16**^**b**^
Progressive disease (PD)		24 (72.73)			24 (100.00)	
PFS (months)						
Median	25.38	2.75	**1.548e-06**^**c**^	25.25	2.50	**1.348e-10**^**c**^
Mean	29.60	9.69		29.40	2.22	
Min–max	3.0–59.0	0.50–53.5		3.0–59.0	0.5–3.5	
SD	18.35	15.18		17.99	0.86	
OS (months)						
Median	45.00	9.25	**1.256e-06**	45	7	**2.741e-09**
Mean	42.76	16.91		41.53	8.78	
Min–max	7.25–59.00	0.50–53.50		7.25–59.00	0.50–32.50	
SD	14.17	16.66		14.19	8.37	

Statistically significant results were marked in bold

PFS, Progression-free survival; OS, Overall survival

^a^Chi-square test

^b^Fisher’s exact test

^c^Kruskal–Wallis rank sum test

The mean progression-free survival (PFS) time of enrolled patients was 19 ± 19.4 months. PFS for the CB group was 29.40 ± 17.99 while NB 2.22 ± 0.86 (*p* < 0.01; 29.60 ± 18.35 and 9.69 ± 15.18 for R and NR, respectively, *p* < 0.01, Table 1, Figs. S1A, B). In our cohort, we observed a 1.84-fold higher mean LDH concentration in the NB group compared to the CB group (393.21 ± 275.50 and 213.84 ± 64.75 units/L, respectively, *p* = 0.01) (Table 1, Fig. S1C). When analyzing R versus NR, NR had a 1.55-fold higher mean LDH concentration than NR (339.79 ± 250.47 and 219.14 ± 71.22 units/L, respectively, *p* = 0.08) (Table 1, Fig. S1D). Ten of 24 (41.67%) patients from NB had LDH serum levels above 338 units/L (> 1.5 × upper limit of normal concentration), and only two of 37 (5.41%) patients from the CB group exceeded the threshold.

### Lymphocytes and patients-related characteristics

Before diving into correlations between gut fungi and lymphocytes, we analyzed correlations between lymphocytes and the cohort’s characteristics, such as age, serum LDH level, overall survival, and progression-free survival (Table 2). Although age differences in the R versus NR and CB versus NB groups were insignificant, age was chosen because of the association between aging and immune system aging, especially given the wide range of this parameter in the study cohort (32–96 years). Serum LDH level was chosen considering its association with response to anti-PD-1 therapy, and OS and PFS were analyzed because of their direct relation to the efficacy of the immune response to ICI therapy, as shown by the calculation of the statistical significance of these parameters in R versus NR and CB versus NB groups (Table 2). To analyze the plausible correlation between the selected factors and the circulating lymphocyte levels, data collected before treatment (n = 61) were analyzed.

**Table 2:** Correlations between melanoma patients’ characteristics and lymphocytes circulating in their blood (Pearson correlation)

Condition	Blood parameter	estimate	*p*.value
Age [years]	Naive Tc % of Cytotoxic Lymphocytes T	− 0.40	< 0.01
	B reg transitional % of Lymphocytes B	− 0.30	0.03
	TD Tc % of Cytotoxic Lymphocytes T	**0.29**	0.04
Serum LDH level [units/L]	Post-germ center B cells % of memory switched Lymphocytes B	**0.51**	< 0.01
	Switched post-germ center % of memory switched Lymphocytes B	− 0.45	< 0.01
	Naive Th % of Regulatory Lymphocytes T	**0.31**	0.03
	Late memory % of Lymphocytes B	**0.29**	0.03
	B cells % of PBMCs	− 0.28	0.04
OS [months]	T cells % of all PBMCs	**0.49**	< 0.01
	T cells % of all Lymphocytes	**0.41**	< 0.01
	Late memory % of Lymphocytes B	− 0.39	< 0.01
	EM Tc % of Cytotoxic Lymphocytes T	**0.37**	< 0.01
	Lymphocytes % of PBMC	**0.35**	0.01
	CD4+/CD8+ % of Lymphocytes T	**0.33**	0.02
	B cells % of PBMCs	**0.30**	0.03
PFS [months]	EM Tc % of Cytotoxic Lymphocytes T	**0.31**	0.03
	Late memory % of Lymphocytes B	− 0.27	0.05
	B cells % of PBMCs	**0.30**	0.03

Only samples before treatment (BT) were taken for analysis. Only statistically significant results are shown, *p* ≤ 0.05

Positive correlations were marked in bold

OS, overall survival; PFS, progression-free survival

Correlation analysis revealed several associations between the different types of lymphocytes and patients-related factors. A complete list of statistically significant correlations is presented in Table 2 and Fig. S2. From the selected factors, OS was correlated with the highest number of tracked lymphocyte types, followed by the serum LDH level. The majority of correlations between analyzed patient-related factors were positive. Inverse relations were observed for serum LDH level and PFS (and OS). When the LDH level rose, the B cells % of PBMCs dropped (*p* = 0.04); conversely, when OS or PSF increased, the B cells % of PBMCs also rose (*p* = 0.03 and *p* = 0.03, respectively). Similar but inverse relations were observed for Late memory % of Lymphocytes B; a positive correlation was shown for Late memory % of Lymphocytes B and serum LDH level (*p* = 0.03), and a negative correlation was spotted between Late memory % of Lymphocytes B and OS (*p* < 0.01) and PFS (*p* = 0.05).

Additionally, for each selected parameter, we divided subjects into two groups based on the given threshold and compared the levels of lymphocytes in the obtained groups (complete list in Table 3, Fig. S3). Most changes in the number of lymphocytes of different types were associated with divisions relative to OS and PFS. Again, opposite directions of changes were observed for groups of patients with increased serum LDH levels and longer OS. In the group of patients with elevated serum LDH, Late memory % of Lymphocytes B and T cells CD273+/CD274+ % of all Lymphocytes were higher (*p* < 0.01), and T cells % of all PBMCs was lower (*p* = 0.04), compared to the group with the lower level of serum LDH. On the contrary, patients with longer OS were characterized by lower levels of Late memory % of Lymphocytes B (*p* < 0.01) and T cells CD273+/CD274+ % of all Lymphocytes (*p* < 0.01), and higher levels of T cells % of all PBMCs (*p* < 0.01). Partly similar changes were observed when analyzing OS and PFS. Patients with longer PFS also had lower levels of Late memory % of Lymphocytes B(*p* = 0.01). Additionally, T cell % of all Lymphocytes was higher in the group with longer OS (*p* < 0.01) and PFS (*p* = 0.03), compared to shorter OS and PFS, respectively.

**Table 3:** Comparison of groups of melanoma patients divided by their characteristics (two-sample Wilcoxon test)

Condition	Blood parameter	Groups		*p*
Age [years]		**< 60 years** (n = 19 mean 49.26 ± 7.03 years)	**≥ 60 years** (n = 42, mean 71.74 ± 8.22 years)	< 0.01
	B reg memory % of Lymphocytes B	**13.79 ± 6.19**	11.39 ± 9.76	0.02
	B reg transitional % of Lymphocytes B	**9.07 ± 4.19**	6.86 ± 3.71	0.04
	Memory switched % of Lymphocytes B	**11.63 ± 4.89**	8.75 ± 6.47	0.03
	Transitional % of Lymphocytes B	**7.25 ± 6.11**	4.24 ± 3.97	0.04
Serum LDH level [units/L]		**LDH < 338 units/L** (n = 49 mean 206.08 ± 43.40 units/L)	**LDH ≥ 338 units/L** (n = 12 mean 604.25 ± 260.48 units/L)	< 0.01
	Late memory % of Lymphocytes B	6.17 ± 6.00	**13.55 ± 15.56**	< 0.01
	T cells CD273+/CD274+ % of all Lymphocytes	0.10 ± 0.14	**0.53 ± 0.66**	< 0.01
	T cells % of all PBMCs	**38.56 ± 11.30**	28.76 ± 12.97	0.04
OS [months]		**OS** $\le$ **12 months** (n = 21 mean 5.69 ± 3.50 months)	**OS > 12 months** (n = 39 mean 41.51 ± 12.81 months)	< 0.01
	B cells % of PBMCs	6.88 ± 5.12	**9.34 ± 3.82**	0.03
	Lymphocytes % of PBMC	60.37 ± 14.04	**68.93 ± 10.20**	0.03
	T cells % of all Lymphocytes	47.14 ± 13.41	**59.05 ± 12.11**	< 0.01
	T cells % of all PBMCs	28.24 ± 10.79	**40.90 ± 10.61**	< 0.01
	Late memory % of Lymphocytes B	**12.58 ± 13.76**	4.99 ± 3.30	< 0.01
	Naive Th % of Regulatory Lymphocytes T	**28.52 ± 12.09**	20.27 ± 15.68	0.05
	T cells CD273+/CD274+ % of all Lymphocytes	**0.37 ± 0.54**	0.08 ± 0.14	< 0.01
PFS [months]		**PFS** $\le$ **12 months** (n = 32 mean 3.82 ± 3.19)	**PFS > 12 months** (n = 28 mean 36.30 ± 15.01)	0.01
	CD27 + Memory % of Lymphocytes B	39.90 ± 14.96	**49.15 ± 16.93**	0.03
	Memory non-switched % of Lymphocytes B	29.03 ± 14.19	**40.99 ± 16.56**	< 0.01
	Non-maturated non-act B cells % of Lymphocytes B	1.25 ± 1.40	**1.93 ± 1.62**	0.02
	Non-maturated act B cells % of all Lymphocytes	27.05 ± 1.72	**34.34 ± 13.32**	0.04
	T cells % of all Lymphocytes	51.77 ± 12.74	**59.10 ± 14.00**	0.03
	CD27- Non-memory % of Lymphocytes B	**60.1 ± 14.96**	50.85 ± 16.93	0.03
	Late memory % of Lymphocytes B	**10.23 ± 11.65**	4.54 ± 2.86	0.01

Only samples before treatment (BT) were taken for the analysis. Only statistically significant results are shown, *p* ≤ 0.05

Higher values in the analyzed group were marked in bold

OS, overall survival; PFS, progression-free survival

### Lymphocytes and anti-PD-1 therapy efficacy

First, we compared the levels of lymphocytes before starting the anti-PD-1 therapy and in the third month of the treatment. We observed that in the T3 group, the levels of B reg transitional % of Lymphocytes B (*p* = 0.03), Naive B cells non-memory % of Lymphocytes B (*p* = 0.05), and T cells PD-1-/CD152- % of all Lymphocytes (*p* < 0.01) were higher compared to the levels observed in the BT group (Table 4, Fig. S4). On the other hand, the level of T cells PD-1+ % of all Lymphocytes (*p* < 0.01) was elevated in BT compared to the T3 group (Table 4, Fig S4). Then, we analyzed whether lymphocyte levels correlate with the outcome of the anti-PD-1 therapy in our cohort. To this end, we compared levels of lymphocytes before treatment between R and NR, and CB and NB, and similarly between these groups but in the third month of the anti-PD-1 therapy (Table 4, Figs. S5 and S6). We found that division into CB and NB resulted in more differences in lymphocyte levels than division into R and NR when analyzing samples before treatment. The pattern of lymphocyte differences in the CB versus NB group before treatment was similar to that from the OS division (OS $\le$ 12 versus OS > 12 months). Some of these differences were also visible in the R versus NR comparison for BT and T3 samples and CB versus NB samples from T3. Only the pattern for Late memory % of Lymphocytes B has reversed when comparing the R versus NR from two time points, BT and T3. Late memory % of Lymphocytes B was lower (*p* = 0.03) in the R group compared to NR when BT samples were examined; conversely, when T3 samples were analyzed, the Late memory % of Lymphocytes B was higher in R (*p* = 0.02). The remaining statistically significant associations are present in Table 4.

**Table 4:** Comparison of groups of melanoma patients divided by treatment time and response to anti-PD-1 therapy (two-sample Wilcoxon test)

Condition	Blood parameter	Groups		*p*
Time of treatment		**BT** (n = 61)	**T3** (n = 37)	
	B reg transitional % of Lymphocytes B	7.62 ± 3.99	**9.69 ± 4.31**	0.03
	Naive B cells non-memory % of Lymphocytes B	48.41 ± 16.17	**54.23 ± 13.94**	0.05
	T cells PD-1-/CD152- % of all Lymphocytes	82.55 ± 6.24	**93.07 ± 5.08**	< 0.01
	T cells PD-1+ % of all Lymphocytes	**17.01 ± 6.21**	6.47 ± 4.91	< 0.01
BT		**CB** (n = 37)	**NB** (n = 24)	
	B cells % of all Lymphocytes	**14.48 ± 5.57**	10.17 ± 7.94	< 0.01
	B cells % of PBMCs	**9.81 ± 3.61**	6.20 ± 4.88	< 0.01
	Memory non-switched % of Lymphocytes B	**38.27 ± 16.57**	28.19 ± 13.67	0.01
	Non-maturated act B cells % of all Lymphocytes	**1.95 ± 1.55**	0.91 ± 1.28	< 0.01
	T cells % of all Lymphocytes	**58.74 ± 12.80**	48.90 ± 12.13	< 0.01
	T cells % of all PBMCs	**40.01 ± 11.17**	31.04 ± 11.95	< 0.01
	T cells CD273+/CD274+ % of all Lymphocytes	0.1 ± 0.15	**0.32 ± 0.53**	0.05
	Late memory % of Lymphocytes B	4.58 ± 2.81	**12.22 ± 12.89**	< 0.01
BT		**R** (n = 28)	**NR** (n = 33)	
	Non-maturated act B cells % of all Lymphocytes	**1.88 ± 1.25**	1.23 ± 1.67	< 0.01
	B cells % of PBMCs	**9.57 ± 3.45**	7.32 ± 5.03	0.03
	Late memory % of Lymphocytes B	4.60 ± 2.68	**10.24 ± 11.67**	0.03
T3		**CB** (n = 29)	**NB** (n = 8)	
	T cells % of all Lymphocytes	**58.36 ± 13.61**	41.53 ± 9.64	< 0.01
	T cells % of all PBMCs	**39.07 ± 12.l7**	27.33 ± 8.15	0.02
	T cells CD273+/CD274+ % of all Lymphocytes	0.09 ± 0.15	**0.18 ± 0.16**	0.04
T3		**R** (n = 22)	**NR** (n = 15)	
	Late memory % of Lymphocytes B	**6.00 ± 3.61**	3.58 ± 1.95	0.02
	T cells % of all Lymphocytes	**58.85 ± 15.54**	49.25 ± 11.17	0.02
	T cells % of all PBMCs	**39.89 ± 13.66**	32.04 ± 8.68	0.04

For testing groups of responses, only samples before treatment (BT) were taken for the analysis. Only statistically significant results are shown, *p* ≤ 0.05

Higher values in the analyzed group were marked in bold

CB, clinical beneficiaries; NB, non-clinical beneficiaries; R, responders; NR, non-responders

### Gut mycobiota of the study cohort

**Fig. 1:**
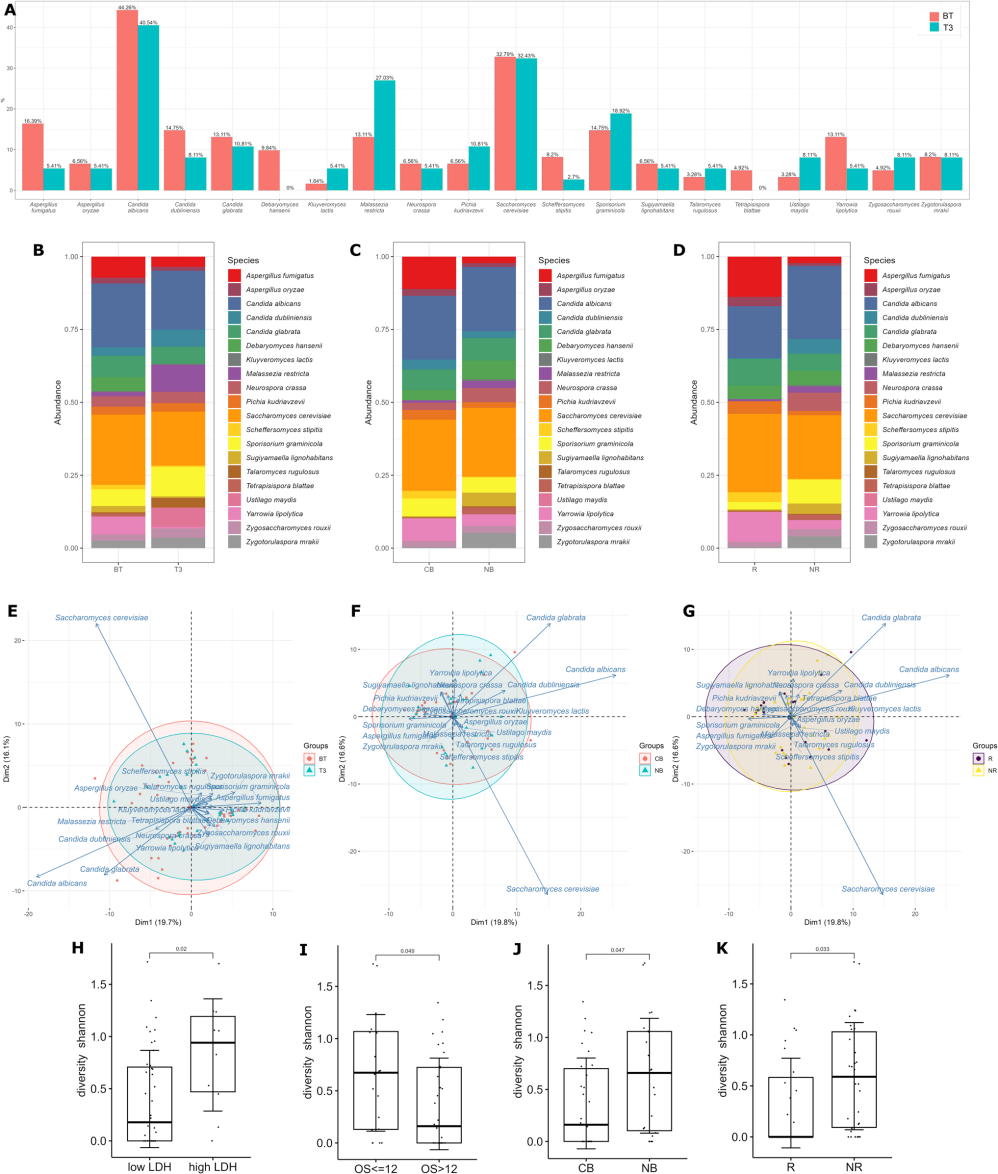
Gut mycobiota of analyzed samples. **A** The percentage of samples in which a particular fungal species was identified. **B**–**D** The relative abundance of fungal species regarding **B** the time of treatment, BT (n = 53) vs. T3 (n = 33), **C** response to anti-PD-1 therapy, CB (n = 30) versus NB (n = 23), and **D** R (n = 23) versus NR (n = 30). **E**–**G** Principal component analysis representation of cohorts distribution based on fungal species composition taking into account the **E** the time of treatment, BT (n = 53) versus T3 (n = 33), **F** response to anti-PD-1 therapy, CB (n = 30) versus NB (n = 23) and **G** R (n = 23) versus NR (n = 30). **H–K** Comparison of Shannon diversity taking into account the selected patient differentiation criteria such as **H** LDH level (low (n = 43) versus high (n = 10)), **I** overall survival (OS, ≤ 12 (n = 20) versus > 12 (n = 32)), (J) CB (n = 30) versus NB (n = 23), and (K) R (n = 23) versus NR (n = 30). The Wilcoxon test was performed to compare groups, p < 0.05. The horizontal line shows the median. The lower and upper hinges correspond to the first and third quartiles. The upper and the lower whiskers extend from the hinge to the largest and smallest value, respectively, no further than 1.5 * IQR from the hinge (where IQR is the inter-quartile range or distance between the first and third quartiles). Dots represent individual samples

Twenty fungal species belonging to 15 genera were detected in at least five stool samples of melanoma patients. *C. albicans*, *S. cerevisiae*, and *Aspergillus fumigatus* were the most common fungal species among the samples BT (present in 44.26%, 32.79%, and 16.39% of the samples, respectively, Fig. 1A). In samples taken three months after the first administration of anti-PD-1 treatment (T3), the most frequently occurring fungal species were *C. albicans*, *S. cerevisiae*, and *M. restricta* (present in 40.54%, 32.43%, and 27.03% of the samples, respectively, Fig. 1A). The most abundant species in BT samples were *S. cerevisiae*, *C. albicans*, and *Candida glabrata* (24.09%, 21.91%, and 7.32%, respectively, Figs. 1B, S7, and S8). In T3 samples, the most abundant were *C. albicans*, S*. cerevisiae*, and *Sporisorium graminicola* (20.20%, 18.46%, and 10.08%, respectively, Figs. 1B, S7 and S8). Statistics for higher taxonomic levels (phylum to genus) are shown in Fig. S7, and the distribution of fungal taxa across samples is shown in Fig. S8.

When analyzing BT in division into CB and NB (and R/NR) in CB and R the most abundant gut fungal species were *S. cerevisiae*, *C. albicans*, and *A. fumigatus* (24.43%, 21.82%, and 11.09% in CB, respectively, and 26.90%, 17.78%, and 13.84% for R, respectively, Figs. 1C, D, S9, S10, S11 and S12). The most abundant species in the NB group were similar to those in BT, namely *S. cerevisiae*, *C. albicans*, and *C. glabrata* (23.64%, 22.02%, and 7.68%, respectively, Figs. 1C, S9, and S10). NR samples were abundant in *C. albicans*, S*. cerevisiae*, and *S. graminicola* (25.07%, 21.93%, and 8.18%, respectively, Figs. 1D, S11, and S12), similarly to T3 samples. Statistics for higher taxonomic levels (phylum to genus) are shown in Fig. S9 (CB vs. NB) and S11 (R. vs. NR), and the distribution of fungal taxa across samples is shown in Figs. S10 (CB vs. NB) and S12 (R. vs. NR).

Analysis of gut mycobiota structure did not show statistically significant differences for any of the compared groups (Figs. 1E, F, G). Shannon diversity of fungi species found in stool samples taken from patients with lower serum LDH levels (< LDH < 338 units/L) was lower than in the stool samples from patients with high levels of serum LDH (*p* = 0.02, Fig. 1H). Also, patients with OS > 12, clinical beneficiaries (CB), and responders (R) had lower levels of Shannon diversity (*p* = 0.05, *p* = 0.05, and *p* = 0.03, respectively, Figs. 1I, J, K).

### Lymphocytes and gut fungi during the anti-PD-1 therapy

**Fig. 2:**
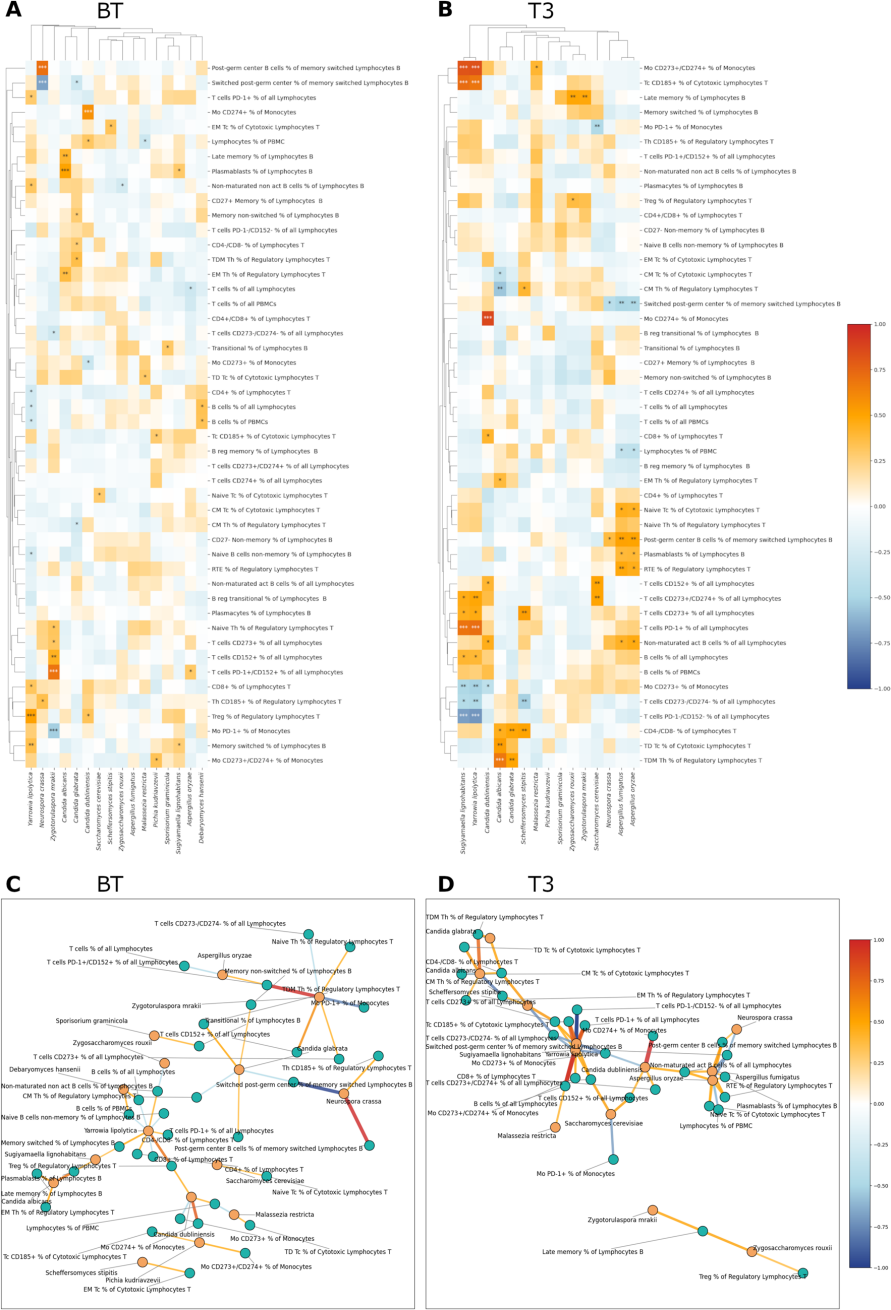
Relationships between gut fungi and lymphocytes regarding the treatment time, BT (n = 53) and T3 (n = 33). Heatmap for **A** samples taken before treatment (BT, n = 53) and **B** in the third month of the therapy (T3, n = 33). Graphs of correlations for (C) BT (n = 53) and (D) T3 (n = 33). Only statistically significant correlations are shown on graphs. Pearson correlation was performed, *p* < 0.05. 0.001***, 0.01**, 0.05*

To discover the relationships between the gut mycobiota of melanoma patients enrolled in anti-PD-1 treatment and their immunological response, we performed a correlation analysis between the abundance of fungal species detected in the stool samples and the number of lymphocytes found in the blood of the patients before the anti-PD-1 therapy (BT, n = 61) and after three months of the treatment (T3, n = 31). We discovered that the abundance of many gut fungal species correlated with the levels of different types of lymphocytes circulating in the blood of melanoma patients, and this view often changed depending on the analyzed time point (BT or T3). All associations are presented in the form of a heatmap (Fig. 2A for BT and Fig. 2B for T3), accompanied by the correlations graph showing statistically significant correlations (Figs. 2C and D for BT and T3, respectively; complete associations list is available also in Table S4).

Generally, in the BT group, we discovered 42 correlations of 35 lymphocytes with 14 fungi. Most of them were positive (n = 29). The fungus with the highest number of correlations was *Yarrowia lipolytica* (n = 9). In BT, strong correlations (*p* < 0.01) were visible for *Y. lipolytica*, *Zygotorulaspora mrakii*, *N. crassa*, and fungi from the *Candida* genus. *C. albicans* and *Candida glabrata* displayed similar patterns of correlations with lymphocytes, and as such, they were clustered together.

In the T3 group, a higher number of correlations was detected than in the BT group (n = 58 vs. n = 42), and the number of positive and negative correlations was similar (30 vs. 29, respectively). In the case of T3, the fungi with the highest number of correlations were *Sugiyamaella lignohabitans* (n = 9) and, similar to BT, *Y. lipolytica* (n = 9). Of notice, even though *Y. lipolytica* was among the fungi with the highest number of detected correlations with lymphocytes both in BT and T3, it displayed different patterns of correlations in these two groups. In T3, *Sugiyamaella lignohabitans* and* Y. lipolytica* were clustered together as they displayed comparable patterns of correlations with lymphocytes; similarly for pairs *C. albicans* and *C. glabrata*, and *A. fumigatus* and *Aspergillus oryzae* which were clustered together. In T3, *S. cerevisiae* showed correlations not detected before treatment, namely positive correlations with T cells CD152+% of all Lymphocytes, T cells CD273+/CD274+ % of all Lymphocytes, and negative with Mo PD-1+ % of Monocytes (Table S4). In this group, fungi often showed correlations with the lymphocytes expressing CD273 and/or CD274. Overall, correlations in T3 were stronger than in the BT group.

**Fig. 3:**
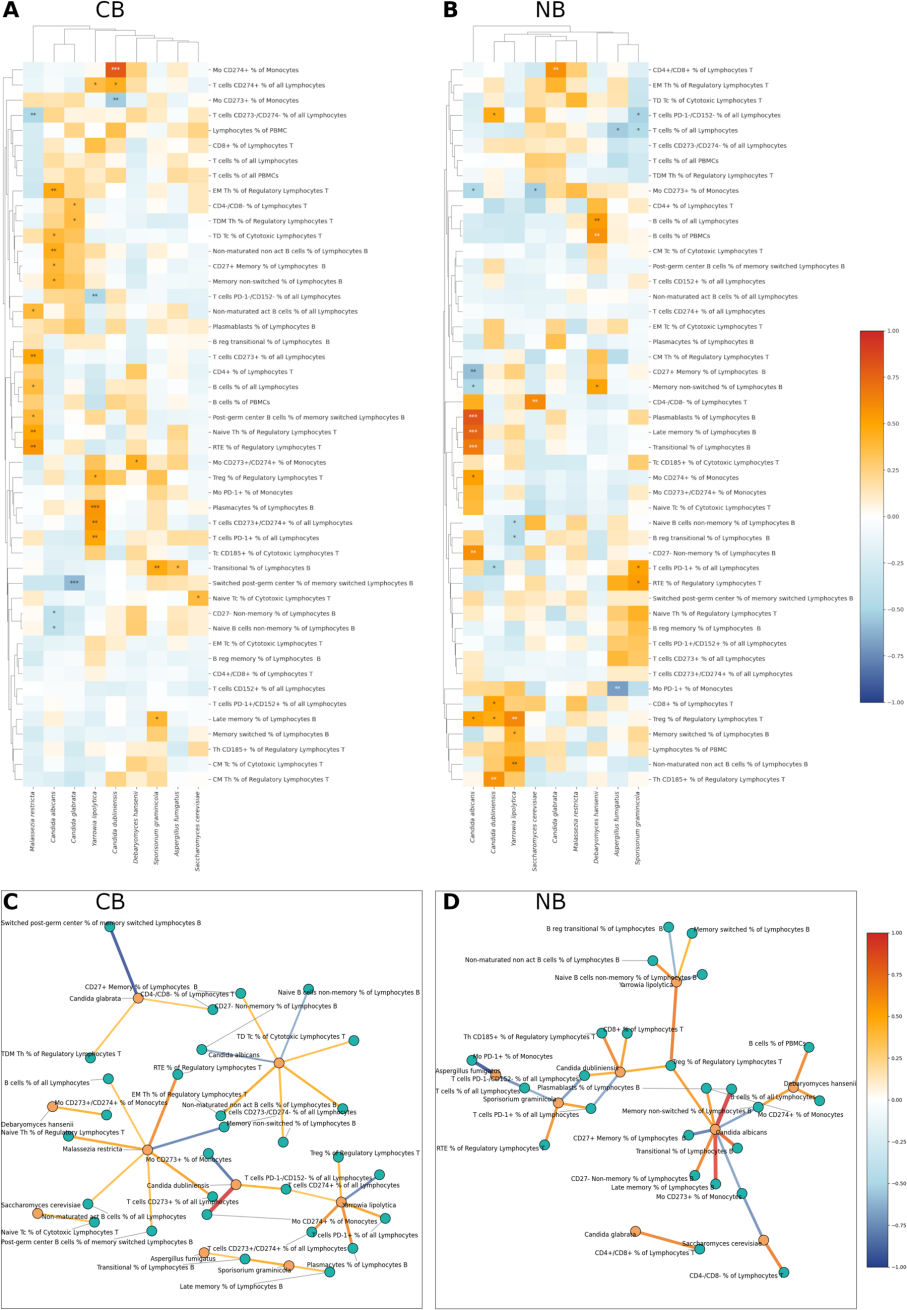
Relationships between gut fungi and lymphocytes regarding the response to anti-PD-1 therapy, CB and NB. Heatmap for **A** clinical beneficiaries (CB, n = 30) and **B** nonbeneficiaries (NB, n = 23). Graphs of correlations for (C) CB (n = 30) and (D) NB (n = 23). Only statistically significant correlations are shown on graphs. Pearson correlation was performed, *p* < 0.05. 0.001***, 0.01**, 0.05*

To discover whether and how the pattern of correlations between gut fungi and lymphocytes changes depending on the response to immunotherapy, we reanalyzed the BT samples, considering the division into CB (n = 37) and NB (n = 24), and R (n = 28) and NR (n = 33) groups. We found out that the correlation pattern was specific to the type of response, positive or negative. A complete list of detected correlations is available in Table S5 for CB and NB groups and Table S6 for R and NR groups, as well as heatmaps and correlation graphs (Figs. 3A, B and 3C, D, respectively). When comparing CB and NB groups, 31 correlations were detected for both CB and NB, in both groups, the majority of correlations were positive (n = 25 for CB, n = 20 for NB). In the CB group, fungi with the highest number of correlations were *C. albicans* (n = 7) and *M. restricta* (n = 7), and in the NB group, *C. albicans* (n = 9). Moreover, correlations with contradictory directions were detected for *C. albicans* when comparing CB and NB. Also, *C. dubliniensis*, *C. glabrata,* and *Y. lipolytica* displayed different correlation patterns depending on the analyzed group, CB or NB. A similar pattern was visible for R and NR (Fig. S13). In CB, *C. albicans* clustered with *C. glabrata*, while in NB, these two fungi displayed different patterns of correlations. In NB, *C. dubliniensis* and *Y. lipolytica* were clustered together, as were *A. fumigatus* and *S. graminicola*. When examining the CB, previously unspotted in BT, *M. restricta* showed up as fungi that exhibited a set of correlations with different types of lymphocytes, with prevalent positive correlations (Fig. 3, Table S5). Moreover, *S. cerevisiae* in the CB group correlated positively with the naïve Tc % of Cytotoxic Lymphocytes T, while in the NB group, we observed a positive correlation with the CD4-/CD8- % of Lymphocytes T and a negative with the Mo CD273 + % of Monocytes (Fig. 3, Table S5). In CB, many fungi positively correlated with the lymphocytes expressing CD273 or/and CD274, while in NB, apart from correlations with the lymphocytes expressing CD273 or/and CD274, correlations with PD-1 T cells and monocytes can be seen.

**Fig. 4:**
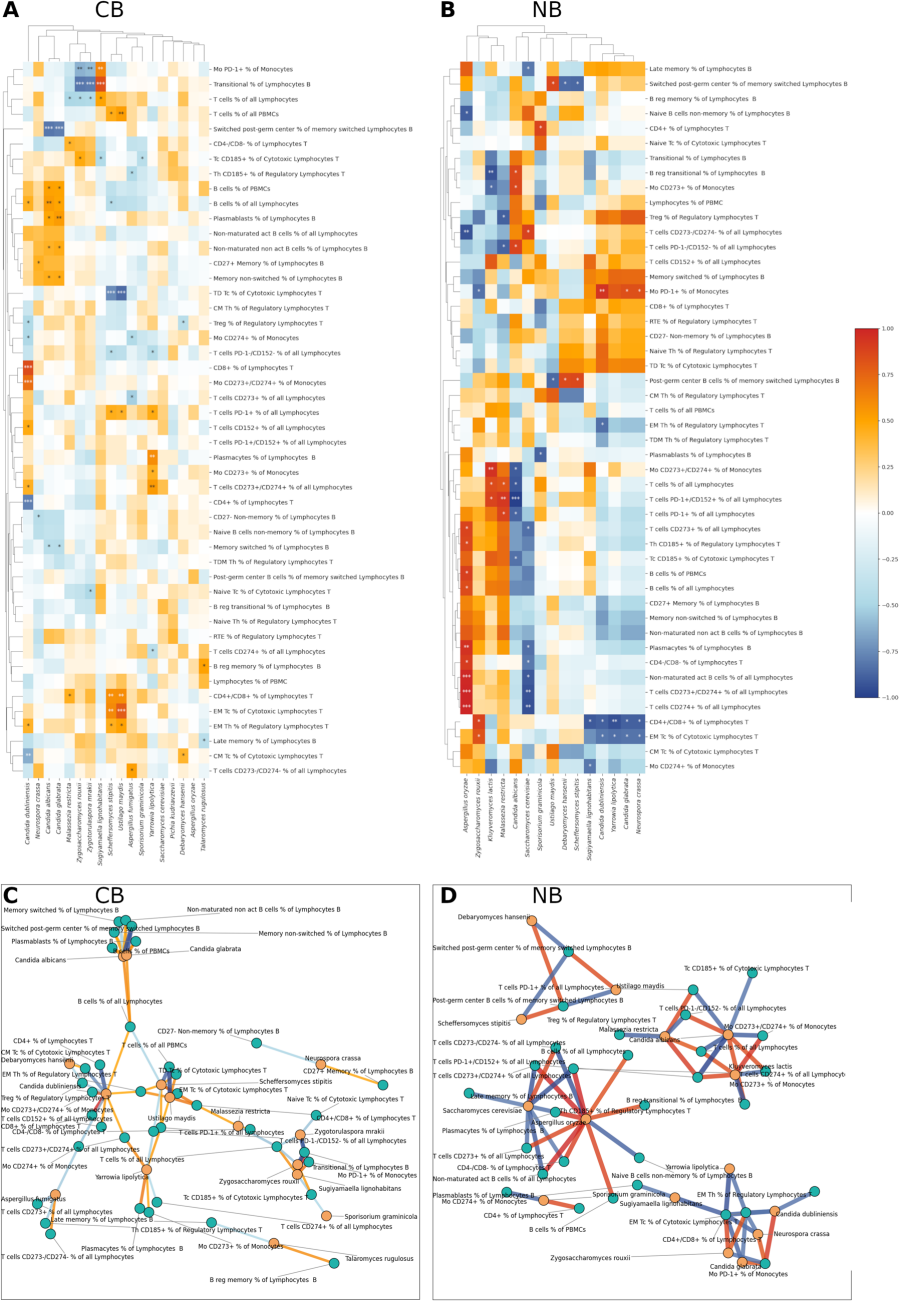
Relationships between changes in gut fungi count and changes in the number of lymphocytes regarding the response to anti-PD-1 therapy, CB and NB. Heatmap for **A** clinical beneficiaries (CB, n = 28) and **B** nonbeneficiaries (NB, n = 8). Graphs of correlations for **C** CB (n = 28) and (D) NB (n = 8). Paired samples from the BT (n = 36) and T3 (n = 36) groups were analyzed. Only statistically significant correlations are shown on graphs. Pearson correlation was performed, *p* < 0.05. 0.001***, 0.01**, 0.05*

To further analyze associations between the gut fungi and lymphocytes, we performed paired samples correlations (Pearson method). We compared the change in counts of each analyzed fungal species, and the change in the number of each analyzed lymphocyte before starting the anti-PD-1 treatment and in the third month of the anti-PD-1 treatment with respect to response to the treatment (CB vs. NB and R vs. NR). For this analysis, we selected only BT (n = 36) and T3 (n = 36) paired samples from our cohort. A complete list of detected significant correlations is available in Table S7 for CB (n = 28) and NB (n = 8) groups and Table S8 for R (n = 22) and NR (n = 14) groups. The correlations are also visualized on heatmaps and correlation graphs (Figs. 4 and S14, respectively).

Again, we found out that the correlation pattern was specific to the type of response, positive or negative. When comparing the CB and NB groups, 70 correlations were detected for the CB group, and 62 for the NB group. Of notice, the NB group was characterized by stronger correlations than the CB group, and in the CB group, most correlations were positive (n = 40), while in the NB, most were negative (n = 34). In the CB group, the fungus with the highest number of correlations was *C. dubliniensis* (n = 10). In the NB group, *Aspergillus oryzae* (n = 11) with nine being positive, followed by *S. cerevisiae* (n = 8) with seven being negative, and *C. albicans* (n = 8). For lymphocytes, B cells % of all Lymphocytes and T cells % of all Lymphocytes displayed the highest number of correlations in the CB group (n = 4, both), with three out of four correlations seen for B cells % of all Lymphocytes with fungi belonging to the *Candida* genus, all of them positive. In the NB group, lymphocytes with the highest number of correlations with fungi were CD4+/CD8+ % of Lymphocytes T (n = 6), EM Tc % of Cytotoxic Lymphocytes T (n = 5), and Mo PD-1+% of Monocytes (n = 4). Correlations with CD4+/CD8+ % of Lymphocytes T and EM Tc % of Cytotoxic Lymphocytes T were mostly positive, while correlations with Mo PD-1+ % of Monocytes were mostly negative. As previously, in CB, *C. albicans* clustered with *C. glabrata* (Fig. 4A), similar to R (Fig. S14A), while in NB, these two fungi displayed different patterns of correlations (Fig. 4B). In NB, a cluster was formed by *C. dubliniensis* and *Y. lipolytica*, additionally clustered with *C. glabrata* and *N. crassa* (Fig. 4B), similar to NR (Fig. S14B).

## Discussion

In this study, we used an extensive metagenomic approach and flow cytometry analyses to discover correlations between gut fungi and lymphocytes circulating in the blood of metastatic melanoma patients enrolled in anti-PD-1 therapy. Using advanced statistical methods, we revealed the complex network of associations between gut fungi in melanoma patients and their immune response. To our knowledge, this is the first study of the gut mycobiome in melanoma patients diving into details of immune response in the context of the anti-PD-1 treatment efficacy.

In our previous research, two fungi were associated with the increased risk of melanoma progression and a poorer response to anti-PD-1 treatment, namely *M. restricta* and *C. albicans*. [16] Both species were previously correlated with various types of cancer, including melanoma [28–34]. Here we show that *M. restricta* in CB patients was positively correlated with B cells % of all Lymphocytes, Naive Th % of Regulatory Lymphocytes T, Non-maturated act B cells % of all Lymphocytes, post-germ center B cells % of all Lymphocytes, RTE % of Regulatory Lymphocytes T, T cells CD152 + % of all Lymphocytes, and negatively with T cells CD273-/CD274- % of all Lymphocytes. Moreover, a paired analysis showed that in the NB group, change in *M. restricta* was strongly positively correlated with the change of T cells PD-1+/CD152+ % of all Lymphocytes, T cells PD-1+ % of all Lymphocytes, T cells % of all Lymphocytes and negatively with Treg % of Regulatory Lymphocytes T and T cells PD-1-/CD152- % of all Lymphocytes. These results could imply a diverse, response-associated, robust immune reaction against *M. restricta*-associated antigens or metabolites in melanoma patients treated with anti-PD1 or even an immune response induced by *M. restricta*.

CD273+ (PD-L2) is coded by the Pdcd1lg2 gene, and its expression level is predictive of clinical response to PD-1-directed immunotherapy [35]. PD-L2 overexpression indicates a poor prognosis in head and neck squamous cell carcinoma (HNSCC) [36], adenoid cystic carcinoma [37], and oesophageal cancer [38]. Among HNSCC patients, an increased expression of PD-L2 was positively correlated to poor relapse-free survival (RFS), PFS, and OS [36]. Consistent with these studies, in our cohort, T cells CD273+/CD274+ % of all lymphocytes was higher in NB group, patients with shorter OS and increased LDH serum level, a known predictor of poor OS in melanoma. The correlation between PD-L2 and adverse cancer prognosis might stem from PD-L2’s stronger binding affinity to the PD-1 receptor, surpassing PD-L1 by 6 to 10 times [39]. This heightened affinity could pose a considerable challenge to therapeutic anti-PD-1 antibodies by competing for receptor binding and engagement, consequently reducing their effectiveness.

Although the exact mechanism of action between *M. restricta* and T cells CD273+ and CD274+ remains unknown, the association of *M. restricta* with worse prognosis for anti-PD-1 patients and its positive correlation with PD-L2 is a fact. It has been shown that *M. restricta* induces a broad range of proinflammatory cytokines and chemokines, including TNFα, IL-6, IL-1β IL-12p40, IL-10, and CXCL1, and stimulates the production of pro-IL-1β as well as activation of the NLRP3 inflammasome, both required for IL-1β production [40]. Typically, IL-1β exhibits a pro-tumor function. However, its role can shift to anti-tumor depending on tumor type or treatment modalities. [41, 42]

For the *C. albicans*, in our study in the BT group, its abundance was positively correlated with EM Th % of Regulatory Lymphocytes T, Late memory % of Lymphocytes B, and Plasmacytes % of Lymphocytes B, suggesting the role of *C. albicans* in the emergence of the long-term adaptive immune response [43]. A more complicated picture was visible when analyzing CB and NB groups separately. In the CB, there was a positive correlation between *C. albicans* and various lymphocytes connected with long-term immunity, such as CD27+ Memory % of Lymphocytes B, EM Th % of Regulatory Lymphocytes T, Memory non-switched % of Lymphocytes B, and Non-maturated non-act B cells % of all Lymphocytes. Similar results were obtained with a paired analysis. Specifically, the Tp55 protein encoded by the CD27 gene is a member of the TNF-receptor superfamily, required for the generation and long-term maintenance of T cell immunity. TNF, which can exhibit both pro- and anti-tumorigenic effects depending on the context [44], is implicated not only in carcinogenesis but also in non-specific resistance against *C. albicans* [45, 46]. This diversified role of TNF highlights its complex involvement in immune responses. Interestingly, TNF blockade has been shown to overcome resistance to anti-PD-1 therapy in experimental melanoma [47], indicating potential cross-talk between TNF signaling and immune checkpoint pathways. The abundance of *C. albicans* in the CB group also correlated positively with the cytotoxic TD lymphocytes, suggesting activation of the antifungal immune reaction in this group. [48]

On the other hand, the abundance of *C. albicans* in the NB group exhibits a strong positive correlation with a subset of lymphocytes responsible for the early antibody response, plasmablasts, and long-term memory late memory lymphocytes, as well as with regulatory T cells. Late memory % of Lymphocytes B also negatively correlated with the OS and PFS, and positively with LDH serum level and lack of benefits from anti-PD-1 treatment in the BT group, suggesting it as a marker of worse prognosis in melanoma. Moreover, in the NB group *C. albicans* negatively correlated with the Mo CD273+ % of Monocytes (PD-L2) and, at the same time, positively with the Mo CD274+ % of Monocytes (PD-L1). Of notice, in the NB group, an increase in *C. albicans* was correlated with a decrease in T cells in general, T cells expressing PD-1, PD-1 and CD 152, cytotoxic T cells expressing CD185, and monocytes expressing CD273 and CD274. At the same time, an increase in *C. albicans* was associated in this group with an increase in monocytes expressing CD273 and PD-1-/CD152- T cells. PD-L1 negatively regulates host antifungal immunity against *C. albicans* infection by inhibiting neutrophil release from the bone marrow [49]. The mechanism seen in both murine and human neutrophils involves the activation of Dectin-1 by fungal β-glucans induced PD-L1 expression. Increased PD-L1 levels control the movement of neutrophils by regulating their self-secretion of CXCL1 and CXCL2. This regulation hinders the release of neutrophils from the bone marrow into the bloodstream, exacerbating *C. albicans* infection. The differences in immune response between NB and CB underscore a potential link between immune responses to fungal infections like *C. albicans* and the efficacy of immunotherapeutic strategies in cancer treatment.

Like *M. restricta*, *C. albicans* can also exist in several morphological growth forms that can have critical implications for commensalism and pathogenic potential [50–52] and further drive the type of immune response [53]. Reversely, immune responses can also provide selective pressure that favors colonization by specific morphotypes [54, 55]. These bi-directional interactions add another layer of complexity to the interactions between gut fungi and the host, with possible critical implications for immune therapy.

In cancer treatment, *S. cerevisiae* enhances the integrity of enterocyte tight junctions, influences host cell signaling, reduces ERK1/2 and EGFR signaling activities, and deactivates tyrosine kinase receptors [56]. Additionally, β-Glucan from *S. cerevisiae* has been shown to activate the immune system in mammals, indicating possible benefits in managing infectious diseases and cancer [57]. In S180 tumor-bearing mice, β-d-Glucan significantly enhanced the mouse immune responses by, among other effects, decreasing the ratio of CD4 to CD8 lymphocytes [58] In our study, in NB patients, *S. cerevisiae* showed a negative correlation with monocytes expressing PD-2L and a positive correlation with CD4-/CD8-lymphocytes expressing CD3. Moreover, in the NB group, the paired analysis revealed a positive correlation between the change in *S. cerevisiae* and T cells CD273-/CD274- % of all Lymphocytes, and negative with T cells expressing PD-2L, PD-1L or both, as well CD4-/CD8- % of Lymphocytes T, Non-maturated act B cells % of all Lymphocytes, Plasmacytes % of Lymphocytes B, and Late memory % of Lymphocytes B. This result may suggest, that in patients showing no benefits from anti-PD-1 treatment (CB), a drop in *S. cerevisiae* abundance may be associated with an increase in PD-1L, PD-2L, and lymphocytes associated with worse progression-free and overall survival, and an increase in serum LDH level.

*D. hansenii*, fungi with antioxidant and antitumor properties [59], showed a positive correlation with the monocytes expressing PD-1L and PD-2L in CB patients. It has been shown that oral administration of this fungi improved immune innate response in mice, upregulating gene expression of pro-inflammatory cytokines (INF-γ, IL-6, and IL-1β) after the *E. coli* challenge [60]. In another animal model, a goat, *D. hansenii*, showed an immunostimulatory effect on leukocytes through β-glucans [61] In this case, the oral administration of *D. hansenii* CBS 8339 stimulated immune response, antioxidant agents, and immune-associated signaling pathways genes in a short time. These findings suggest *D. hansenii* is a promising agent for further research into enhancing the efficacy of immunotherapy.

One of the fungi that displayed a diverse set of correlations was *Y. lipolytica*. This species, previously known as *Candida lipolytica*, apart from its widespread nature [62, 63], has been reported as a human colonizer and weak human pathogen [64]. It was especially associated with bloodstream infections in immunocompromised patients [65, 66]. It has also been shown that *Y. lipolytica* L-asparaginase inhibits the growth and migration of lung and breast cancer cells [67], and it can be used to treat acute lymphocytic leukemia, Human Burkitt's lymphoma, and non-Hodgkin's lymphoma [68]. In the case of the CB patients, we observed positive correlations between *Y. lipolytica* and T cells expressing PD-1L or PD-1L and PD-2L, as well as PD-1 T cells, the pattern not seen in NB patients, suggesting a possible positive impact of this yeast on the melanoma patients response to the anti-PD-1 therapy. This was also confirmed by the paired analysis, in the CB group, an increase in *Y. lipolytica* was associated with an increase in monocytes expressing PD-2L, T cells expressing PD-1L and PD-2L, as well as PD-1 T cells, and a drop in PD-1-/CD152- T cells. On the other hand, in the NB group, there was a negative correlation between the change in *Y. lipolytica* abundance and CD4+/CD8+ % of Lymphocytes T and EM Tc % of Cytotoxic Lymphocytes T.

Our findings underscore the complex interplay between immune responses to gut fungi and the effectiveness of cancer immunotherapy, pointing to avenues for further investigation into how fungal-immune interactions might be leveraged for therapeutic benefit in cancer patients. Given the intricate network of interactions within the gut microbiome, particularly in the context of chronic inflammation and cancer-induced systemic disequilibrium, further research is needed to clarify the mechanisms by which specific fungal taxa may affect the efficacy of immunotherapy. Furthermore, considering the ecological correlation between fungi and the bacterial microbiome, future studies should incorporate a comprehensive analysis of both microbial components. Such a holistic approach may provide a clearer understanding of the diverse outcomes observed in previous research. By further exploring these interactions, we can uncover valuable insights into the complex dynamics of gut microbial communities and their impact on cancer immunotherapy, potentially guiding the development of more effective treatment strategies.

The present study has several limitations that need to be addressed in the future. Although we used a state-of-the-art bioinformatics pipeline to assign individual reads to proper taxa, community profiling still potentially harbors errors due to the genetic similarity of some fungal species. The genetic similarity between certain fungal species complicates the taxonomic resolution in metagenomic analyses, making it difficult to assign fungal species with certainty to specific immune cell interactions. Additionally, the multifactorial nature of immune responses further complicates pinpointing precise associations between individual fungal species and lymphocyte subtypes. Finally, intervention studies would be necessary to derive precise mechanistic insights into gut fungi associations with response or nonresponse to ICI treatment and to confirm the direction of causality. Until then, we believe that this work provides valuable insights to help future research and development in this field, highlighting the role of gut fungi in the modulation of immune response to ICI treatment and opening avenues for further research directed at understanding the role of gut mycobiota in effective cancer treatment and personalized medicine.

## Supplementary Information

Below is the link to the electronic supplementary material.

Supplementary file1 (XLSX 58 KB)

Supplementary file2 (PDF 6831 KB)

## Data Availability

Data generated in this study are available within the article and its Supplementary Data Files or from the corresponding author upon reasonable request. The raw NGS data is available in the NCBI SRA repository (https://www.ncbi.nlm.nih.gov/sra) under accession number PRJNA972625.
