# Supplementary material for: Exploring correlations between gut mycobiome and lymphocytes in melanoma patients undergoing anti-PD-1 therapy
Source: Cancer Immunol Immunother. 2025 Feb 25;74(4):110. doi: 10.1007/s00262-024-03918-9 (PMC11861499; doi:10.1007/s00262-024-03918-9)

# SUPPLEMENTARY TABLES

**Table S1. Patients' data.** Available as a separate Excel file.

**Table S2. A detailed list of monoclonal antibodies (mAbs) used throughout the study.**

| Antibody | Catalog number | Producer |
|---|---|---|
| **FITC Mouse Anti-Human CD27** | 555440 | BD Biosciences |
| **PE Mouse Anti-Human CCR7 (CD197)** | 566741 | BD Biosciences |
| **PerCP-Cy5.5 Mouse Anti-Human CD4** | 566923 | BD Biosciences |
| **PE-Cy7 Mouse Anti-Human CD8** | 335822 | BD Biosciences |
| **APC Mouse Anti-Human CD45RA** | 550855 | BD Biosciences |
| **APC-Cy7 Mouse Anti-Human CD3** | 557832 | BD Biosciences |
| **Alexa Fluor 488 Rat Anti-Human CD185** | 558112 | BD Biosciences |
| **PE Mouse Anti-Human CD45RO** | 555493 | BD Biosciences |
| **FITC Mouse Anti-Human CD127** | 560549 | BD Biosciences |
| **PE Mouse Anti-Human CD31** | 555446 | BD Biosciences |
| **PE-Cy7 Mouse Anti-Human CD25** | 302612 | Biolegend |
| **PE Mouse Anti-Human IgD** | 555779 | BD Biosciences |
| **PerCP-Cy5.5 Mouse Anti-Human IgM** | 561285 | BD Biosciences |
| **PE-Cy7 Mouse Anti-Human CD21** | 561374 | BD Biosciences |
| **APC Mouse Anti-Human CD38** | 555462 | BD Biosciences |
| **APC-Cy7 Mouse Anti-Human CD19** | 557791 | BD Biosciences |
| **PE Mouse Anti-Human CD24** | 555428 | BD Biosciences |
| **PerCP-Cy5.5 Mouse Anti-Human CD138** | 564605 | BD Biosciences |
| **FITC Mouse Anti-Human CD274** | 558065 | BD Biosciences |
| **PE Mouse Anti-Human CD273** | 345506 | Biolegend |
| **PerCP-Cy5.5 Mouse Anti-Human CD14** | 562692 | BD Biosciences |
| **PE-Cy7 Mouse Anti-Human CD279 (PD-1)** | 329918 | Biolegend |
| **APC Mouse Anti-Human CD152** | 555855 | BD Biosciences |

**Table S3. Definition for lymphocytes' nomenclature used throughout the study.**

| Name | Definition |
|---|---|
| **B cells % of all Lymphocytes** | Lymphocytes CD19+ (B lymphocytes in the total lymphocyte population) |
| **B cells % of PBMCs** | Lymphocytes CD19+ in PBMC (in the entire initial sample) |
| **B reg memory % of Lymphocytes B** | Lymphocytes CD19+/CD27+/CD24+ |
| **B reg transitional % of Lymphocytes B** | Lymphocytes CD19+/CD38+/CD24+ |
| **CD27+ Memory % of Lymphocytes B** | Lymphocytes CD19+/CD27+ |
| **CD27- Non-memory % of Lymphocytes B** | Lymphocytes CD19+/CD27- |
| **CD4+ % of Lymphocytes T** | Lymphocytes CD3+/CD4+/CD8- |
| **CD4+/CD8+ % of Lymphocytes T** | Lymphocytes CD3+/CD4+/CD8+ |
| **CD4-/CD8- % of Lymphocytes T** | Lymphocytes CD3+/CD4-/CD8- |
| **CD8+ % of Lymphocytes T** | Lymphocytes CD3+/CD4-/CD8+ |
| **CM Tc % of Cytotoxic Lymphocytes T** | Lymphocytes CD3+/CD4-/CD8+/CD45RA-/CD27+/CD197+ |
| **CM Th % of Regulatory Lymphocytes T** | Lymphocytes CD3+/CD4+/CD8-/CD45RA-/CD27+ |
| **EM Tc % of Cytotoxic Lymphocytes T** | Lymphocytes CD3+/CD4-/CD8+/CD45RA-/CD27-/CD197+ |
| **EM Th % of Regulatory Lymphocytes T** | Lymphocytes CD3+/CD4+/CD8-/CD45RA-/CD27- |
| **Late memory % of Lymphocytes B** | Lymphocytes CD19+/IgD-/CD27- |
| **Lymphocytes % of PBMC** | Lymphocytes in all PBMC |
| **Memory non-switched % of Lymphocytes B** | Lymphocytes CD19+/IgD+/CD27+ |
| **Memory switched % of Lymphocytes B** | Lymphocytes CD19+/IgD-/CD27+ |
| **Mo CD273+ % of Monocytes** | Monocytes CD14+/CD3-/CD273+/CD274- |
| **Mo CD273+/CD274+ % of Monocytes** | Monocytes CD14+/CD3-/CD273+/CD274+ |
| **Mo CD274+ % of Monocytes** | Monocytes CD14+/CD3-/CD273-/CD274+ |
| **Mo PD-1+ % of Monocytes** | Monocytes CD14+/CD3-/PD1+/CD152- |
| **Naive B cells non-memory % of Lymphocytes B** | Lymphocytes CD19+/CD27-/IgD+ |
| **Naive Tc % of Cytotoxic Lymphocytes T** | Lymphocytes CD3+/CD4-/CD8+/CD45RA+/CD27+/CD197+ |
| **Naive Th % of Regulatory Lymphocytes T** | Lymphocytes CD3+/CD4+/CD8-/CD45RA+/CD27+ |
| **Non-maturated act B cells % of all Lymphocytes** | Lymphocytes CD19+/CD38+/CD21- |
| **Non-maturated non act B cells % of Lymphocytes B** | Lymphocytes CD19+CD21-/CD38+- |

| | |
|---|---|
| **Plasmablasts % of Lymphocytes B** | Lymphocytes CD19+CD38+/IgM- |
| **Plasmacytes % of Lymphocytes B** | Lymphocytes CD19+ |
| **Post-germ center B cells % of memory switched Lymphocytes B** | Lymphocytes CD19+/IgD-/CD27+/IgM+ (subpopulation of Memory switched) |
| **RTE % of Regulatory Lymphocytes T** | Lymphocytes CD3+/CD4+/CD45RA+/CD31+ |
| **Switched post-germ center % of memory switched Lymphocytes B** | Lymphocytes CD19+/IgD-/CD27+/IgM- |
| **T cells % of all Lymphocytes** | Lymphocytes CD3+ |
| **T cells % of all PBMCs** | Lymphocytes CD3+ |
| **T cells CD152+ % of all Lymphocytes** | Lymphocytes CD3+/CD14-/CD152+ |
| **T cells CD273+ % of all Lymphocytes** | Lymphocytes CD3+/CD14-/CD273+ |
| **T cells CD273+/CD274+ % of all Lymphocytes** | Lymphocytes CD3+/CD14-/CD273+/CD274+ |
| **T cells CD273-/CD274- % of all Lymphocytes** | Lymphocytes CD3+/CD14-/CD273-/CD274- |
| **T cells CD274+ % of all Lymphocytes** | Lymphocytes CD3+/CD14-/CD274+ |
| **T cells PD-1+ % of all Lymphocytes** | Lymphocytes CD3+/CD14-/PD1+ |
| **T cells PD-1+/CD152+ % of all Lymphocytes** | Lymphocytes CD3+/CD14-/PD1+/CD152+ |
| **T cells PD-1-/CD152- % of all Lymphocytes** | Lymphocytes CD3+/CD14-/PD1-/CD152- |
| **Tc CD185+ % of Cytotoxic Lymphocytes T** | Lymphocytes CD3+/CD4-/CD8+/CD45RO+/CD185+ |
| **TD Tc % of Cytotoxic Lymphocytes T** | Lymphocytes CD3+/CD4-/CD8+/CD45RA+/CD27-/CD197- |
| **TDM Th % of Regulatory Lymphocytes T** | Lymphocytes CD3+/CD4+/CD8-/CD45RA+/CD27- |
| **Th CD185+ % of Regulatory Lymphocytes T** | Lymphocytes CD3+/CD4+/CD8-/CD45RO+/CD185+ |
| **Transitional % of Lymphocytes B** | Lymphocytes CD19+CD38+/IgM+ |
| **Treg % of Regulatory Lymphocytes T** | Lymphocytes CD3+/CD4+/CD25++/CD127- |

**Table S4. Results of correlation analysis between gut fungi detected in analyzed samples and different types of lymphocytes circulating in the blood of melanoma patients in BT (n=61) and T3 (n=37) groups.** Pearson correlation was performed, $p<0.05$.

| Fungi species | Lymphocytes | corr coef BT | pval BT | corr coef T3 | pval T3 | signif BT | signif T3 |
|---|---|---|---|---|---|---|---|
| ***Aspergillus fumigatus*** | Lymphocytes % of PBMC | 0.084 | 0.563 | **-0.375** | **0.045** | | * |
| ***Aspergillus fumigatus*** | Naive Tc % of Cytotoxic Lymphocytes T | -0.036 | 0.804 | **0.449** | **0.015** | | * |
| ***Aspergillus fumigatus*** | Non-maturated act B cells % of all Lymphocytes | 0.159 | 0.247 | **0.428** | **0.021** | | * |
| ***Aspergillus fumigatus*** | Plasmablasts % of Lymphocytes B | -0.014 | 0.919 | **0.387** | **0.038** | | * |
| ***Aspergillus fumigatus*** | Post-germ center B cells % of memory switched Lymphocytes B | -0.104 | 0.455 | **0.511** | **0.005** | | ** |
| ***Aspergillus fumigatus*** | RTE % of Regulatory Lymphocytes T | 0.236 | 0.099 | **0.478** | **0.009** | | ** |
| ***Aspergillus fumigatus*** | Switched post-germ center % of memory switched Lymphocytes B | 0.128 | 0.357 | **-0.499** | **0.006** | | ** |
| ***Aspergillus oryzae*** | Lymphocytes % of PBMC | -0.096 | 0.507 | **-0.384** | **0.039** | | * |
| ***Aspergillus oryzae*** | Naive Tc % of Cytotoxic Lymphocytes T | 0.146 | 0.312 | **0.432** | **0.019** | | * |
| ***Aspergillus oryzae*** | Non-maturated act B cells % of all Lymphocytes | 0.031 | 0.820 | **0.467** | **0.011** | | * |
| ***Aspergillus oryzae*** | Plasmablasts % of Lymphocytes B | -0.059 | 0.667 | **0.392** | **0.035** | | * |
| ***Aspergillus oryzae*** | Post-germ center B cells % of memory switched Lymphocytes B | -0.064 | 0.645 | **0.502** | **0.005** | | ** |
| ***Aspergillus oryzae*** | RTE % of Regulatory Lymphocytes T | -0.007 | 0.961 | **0.447** | **0.015** | | * |
| ***Aspergillus oryzae*** | Switched post-germ center % of memory switched Lymphocytes B | 0.076 | 0.583 | **-0.488** | **0.007** | | ** |
| ***Aspergillus oryzae*** | T cells % of all Lymphocytes | **-0.305** | **0.032** | -0.068 | 0.727 | * | |
| ***Aspergillus oryzae*** | T cells PD-1+/CD152+ % of all Lymphocytes | **0.298** | **0.036** | -0.001 | 0.997 | * | |
| ***Candida albicans*** | CD4-/CD8- % of Lymphocytes T | 0.204 | 0.155 | **0.438** | **0.017** | | * |
| ***Candida albicans*** | CM Tc % of Cytotoxic Lymphocytes T | -0.011 | 0.940 | **-0.376** | **0.044** | | * |
| ***Candida albicans*** | CM Th % of Regulatory Lymphocytes T | -0.195 | 0.175 | **-0.557** | **0.002** | | ** |
| ***Candida albicans*** | EM Th % of Regulatory Lymphocytes T | **0.369** | **0.008** | **0.376** | **0.044** | ** | * |
| ***Candida albicans*** | Late memory % of Lymphocytes B | **0.407** | **0.002** | 0.089 | 0.648 | ** | |
| ***Candida albicans*** | Plasmablasts % of Lymphocytes B | **0.502** | **0.000** | -0.284 | 0.135 | *** | |
| ***Candida albicans*** | TD Tc % of Cytotoxic Lymphocytes T | 0.255 | 0.074 | **0.524** | **0.004** | | ** |
| ***Candida albicans*** | TDM Th % of Regulatory Lymphocytes T | 0.214 | 0.135 | **0.672** | **0.000** | | *** |
| ***Candida dubliniensis*** | CD8+ % of Lymphocytes T | 0.270 | 0.058 | **0.414** | **0.026** | | * |
| ***Candida dubliniensis*** | Lymphocytes % of PBMC | **0.311** | **0.028** | 0.024 | 0.902 | * | |
| ***Candida dubliniensis*** | Mo CD273+ % of Monocytes | **-0.323** | **0.022** | **-0.395** | **0.034** | * | * |
| ***Candida dubliniensis*** | Mo CD274+ % of Monocytes | **0.576** | **0.000** | **0.863** | **0.000** | *** | *** |
| ***Candida dubliniensis*** | Non-maturated act B cells % of all Lymphocytes | 0.041 | 0.768 | **0.448** | **0.015** | | * |
| ***Candida dubliniensis*** | T cells CD152+ % of all Lymphocytes | -0.093 | 0.518 | **0.405** | **0.029** | | * |
| ***Candida dubliniensis*** | Treg % of Regulatory Lymphocytes T | **0.324** | **0.022** | 0.033 | 0.866 | * | |
| ***Candida glabrata*** | CD4-/CD8- % of Lymphocytes T | **0.281** | **0.048** | **0.485** | **0.008** | * | ** |
| ***Candida glabrata*** | CM Th % of Regulatory Lymphocytes T | **-0.280** | **0.049** | -0.329 | 0.081 | * | |
| ***Candida glabrata*** | Memory non-switched % of Lymphocytes B | **0.276** | **0.041** | -0.057 | 0.770 | * | |
| ***Candida glabrata*** | Switched post-germ center % of memory switched Lymphocytes B | **-0.309** | **0.023** | 0.014 | 0.943 | * | |
| ***Candida glabrata*** | TDM Th % of Regulatory Lymphocytes T | **0.359** | **0.011** | **0.499** | **0.006** | * | ** |
| ***Malassezia restricta*** | Lymphocytes % of PBMC | -0.312 | 0.028 | **0.211** | **0.271** | * | |
| ***Malassezia restricta*** | Mo CD273+/CD274+ % of Monocytes | -0.191 | 0.184 | **0.399** | **0.032** | | * |

| | | | | | | | |
|---|---|---|---|---|---|---|---|
| ***Malassezia restricta*** | TD Tc % of Cytotoxic Lymphocytes T | **0.302** | **0.033** | -0.193 | 0.316 | * | |
| ***Neurospora crassa*** | Post-germ center B cells % of memory switched Lymphocytes B | **0.706** | **0.000** | **0.368** | **0.050** | *** | * |
| ***Neurospora crassa*** | Switched post-germ center % of memory switched Lymphocytes B | **-0.677** | **0.000** | **-0.414** | **0.025** | *** | * |
| ***Neurospora crassa*** | Th CD185+ % of Regulatory Lymphocytes T | **0.344** | **0.014** | 0.233 | 0.224 | * | |
| ***Pichia kudriavzevii*** | Mo CD273+/CD274+ % of Monocytes | **0.334** | **0.018** | -0.107 | 0.579 | * | |
| ***Pichia kudriavzevii*** | Tc CD185+ % of Regulatory Lymphocytes T | **0.284** | **0.046** | -0.007 | 0.970 | * | |
| ***Saccharomyces cerevisiae*** | Mo PD-1+ % of Monocytes | -0.114 | 0.432 | **-0.476** | **0.009** | | ** |
| ***Saccharomyces cerevisiae*** | Naive Tc % of Cytotoxic Lymphocytes T | **0.284** | **0.046** | 0.243 | 0.204 | * | |
| ***Saccharomyces cerevisiae*** | T cells CD152+ % of all Lymphocytes | -0.174 | 0.228 | **0.495** | **0.006** | | ** |
| ***Saccharomyces cerevisiae*** | T cells CD273+/CD274+ % of all Lymphocytes | -0.100 | 0.490 | **0.497** | **0.006** | | ** |
| ***Scheffersomyces stipitis*** | CD4-/CD8- % of Lymphocytes T | -0.056 | 0.701 | **0.507** | **0.005** | | ** |
| ***Scheffersomyces stipitis*** | CM Th % of Regulatory Lymphocytes T | -0.051 | 0.723 | **0.400** | **0.032** | | * |
| ***Scheffersomyces stipitis*** | EM Tc % of Cytotoxic Lymphocytes T | **0.325** | **0.021** | 0.243 | 0.204 | * | |
| ***Scheffersomyces stipitis*** | T cells CD273-/CD274- % of all Lymphocytes | 0.026 | 0.858 | **-0.517** | **0.004** | | ** |
| ***Scheffersomyces stipitis*** | T cells CD273+ % of all Lymphocytes | -0.020 | 0.889 | **0.524** | **0.004** | | ** |
| ***Sporisorium graminicola*** | Transitional % of Lymphocytes B | **0.304** | **0.024** | -0.168 | 0.384 | * | |
| ***Sugiyamaella lignohabitans*** | B cells % of all Lymphocytes | -0.121 | 0.379 | **0.402** | **0.031** | | * |
| ***Sugiyamaella lignohabitans*** | Memory switched % of Lymphocytes B | **0.271** | **0.045** | -0.081 | 0.677 | * | |
| ***Sugiyamaella lignohabitans*** | Mo CD273+ % of Monocytes | 0.018 | 0.901 | **-0.486** | **0.008** | | ** |
| ***Sugiyamaella lignohabitans*** | Mo CD273+/CD274+ % of Monocytes | 0.197 | 0.170 | **0.815** | **0.000** | | *** |
| ***Sugiyamaella lignohabitans*** | Plasmablasts % of Lymphocytes B | **0.296** | **0.028** | -0.156 | 0.420 | * | |
| ***Sugiyamaella lignohabitans*** | T cells CD273-/CD274- % of all Lymphocytes | 0.011 | 0.942 | **-0.445** | **0.015** | | * |
| ***Sugiyamaella lignohabitans*** | T cells CD273+ % of all Lymphocytes | -0.011 | 0.942 | **0.439** | **0.017** | | * |
| ***Sugiyamaella lignohabitans*** | T cells CD273+/CD274+ % of all Lymphocytes | 0.082 | 0.571 | **0.460** | **0.012** | | * |
| ***Sugiyamaella lignohabitans*** | T cells PD-1-/CD152- % of all Lymphocytes | -0.206 | 0.150 | **-0.686** | **0.000** | | *** |
| ***Sugiyamaella lignohabitans*** | T cells PD-1+ % of all Lymphocytes | 0.217 | 0.130 | **0.692** | **0.000** | | *** |
| ***Sugiyamaella lignohabitans*** | Tc CD185+ % of Regulatory Lymphocytes T | 0.241 | 0.092 | **0.719** | **0.000** | | *** |
| ***Yarrowia lipolytica*** | B cells % of all Lymphocytes | **-0.268** | **0.048** | **0.392** | **0.035** | * | * |
| ***Yarrowia lipolytica*** | B cells % of PBMCs | **-0.267** | **0.049** | 0.263 | 0.168 | * | |
| ***Yarrowia lipolytica*** | CD4+ % of Lymphocytes T | **-0.280** | **0.049** | 0.138 | 0.475 | * | |
| ***Yarrowia lipolytica*** | CD8+ % of Lymphocytes T | **0.319** | **0.024** | -0.208 | 0.280 | * | |
| ***Yarrowia lipolytica*** | Memory switched % of Lymphocytes B | **0.351** | **0.009** | -0.128 | 0.507 | ** | |
| ***Yarrowia lipolytica*** | Mo CD273+ % of Monocytes | -0.098 | 0.499 | **-0.514** | **0.004** | | ** |
| ***Yarrowia lipolytica*** | Mo CD273+/CD274+ % of Monocytes | 0.230 | 0.107 | **0.826** | **0.000** | | *** |
| ***Yarrowia lipolytica*** | Naive B cells non-memory % of Lymphocytes B | **-0.271** | **0.045** | 0.121 | 0.532 | * | |
| ***Yarrowia lipolytica*** | Non-maturated non act B cells % of all Lymphocytes | **0.309** | **0.022** | 0.068 | 0.727 | * | |
| ***Yarrowia lipolytica*** | T cells CD273-/CD274- % of all Lymphocytes | 0.109 | 0.452 | **-0.474** | **0.009** | | ** |
| ***Yarrowia lipolytica*** | T cells CD273+ % of all Lymphocytes | -0.115 | 0.425 | **0.467** | **0.011** | | * |
| ***Yarrowia lipolytica*** | T cells CD273+/CD274+ % of all Lymphocytes | 0.097 | 0.501 | **0.486** | **0.008** | | ** |
| ***Yarrowia lipolytica*** | T cells PD-1-/CD152- % of all Lymphocytes | -0.273 | 0.055 | **-0.714** | **0.000** | | *** |

| | | | | | | | |
|---|---|---|---|---|---|---|---|
| ***Yarrowia lipolytica*** | T cells PD-1+ % of all Lymphocytes | **0.288** | **0.042** | **0.718** | **0.000** | * | *** |
| ***Yarrowia lipolytica*** | Tc CD185+ % of Regulatory Lymphocytes T | 0.226 | 0.115 | **0.683** | **0.000** | | *** |
| ***Yarrowia lipolytica*** | Treg % of Regulatory Lymphocytes T | **0.509** | **0.000** | 0.264 | 0.167 | *** | |
| ***Zygosaccharomyces rouxii*** | Late memory % of Lymphocytes B | -0.058 | 0.675 | **0.486** | **0.007** | | ** |
| ***Zygosaccharomyces rouxii*** | Non-maturated non act B cells % of all Lymphocytes | **-0.273** | **0.044** | -0.234 | 0.222 | * | |
| ***Zygosaccharomyces rouxii*** | Treg % of Regulatory Lymphocytes T | 0.023 | 0.873 | **0.374** | **0.046** | | * |
| ***Zygotorulaspora mrakii*** | Late memory % of Lymphocytes B | -0.051 | 0.710 | **0.480** | **0.008** | | ** |
| ***Zygotorulaspora mrakii*** | Mo PD-1+ % of Monocytes | **-0.525** | **0.000** | -0.123 | 0.526 | *** | |
| ***Zygotorulaspora mrakii*** | Naive Th % of Regulatory Lymphocytes T | **0.313** | **0.027** | -0.169 | 0.382 | * | |
| ***Zygotorulaspora mrakii*** | T cells CD152+ % of all Lymphocytes | **0.435** | **0.002** | -0.028 | 0.886 | ** | |
| ***Zygotorulaspora mrakii*** | T cells CD273-/CD274- % of all Lymphocytes | **-0.289** | **0.042** | 0.140 | 0.470 | * | |
| ***Zygotorulaspora mrakii*** | T cells CD273+ % of all Lymphocytes | **0.311** | **0.028** | -0.138 | 0.477 | * | |
| ***Zygotorulaspora mrakii*** | T cells PD-1+/CD152+ % of all Lymphocytes | **0.693** | **0.000** | 0.106 | 0.585 | *** | |

Statistically significant correlations were marked in **bold**. 0.001***, 0.01**, 0.05*

**Table S5. Results of correlation analysis between gut fungi detected in analyzed samples and different types of lymphocytes circulating in the blood of melanoma patients in CB (n=37) and NB (n=24) groups.** Only samples from the BT group were analyzed. Pearson correlation was performed, $p<0.05$.

| Fungi species | Lymphocytes | corr coef CB | pval CB | corr coef NB | pval NB | signif CB | signif NB |
|---|---|---|---|---|---|---|---|
| ***Aspergillus fumigatus*** | Mo PD-1+ % of Monocytes | 0.113 | 0.544 | **-0.691** | **0.001** | | ** |
| ***Aspergillus fumigatus*** | T cells % of all Lymphocytes | 0.131 | 0.482 | **-0.539** | **0.017** | | * |
| ***Aspergillus fumigatus*** | Transitional % of Lymphocytes B | **0.363** | **0.041** | -0.107 | 0.628 | * | |
| ***Candida albicans*** | CD27- Non-memory % of Lymphocytes B | **-0.385** | **0.029** | **0.586** | **0.003** | * | ** |
| ***Candida albicans*** | CD27+ Memory % of Lymphocytes B | **0.385** | **0.029** | **-0.586** | **0.003** | * | ** |
| ***Candida albicans*** | EM Th % of Regulatory Lymphocytes T | **0.468** | **0.008** | 0.003 | 0.990 | ** | |
| ***Candida albicans*** | Late memory % of Lymphocytes B | 0.073 | 0.693 | **0.754** | **0.000** | | *** |
| ***Candida albicans*** | Memory non-switched % of Lymphocytes B | **0.407** | **0.021** | **-0.490** | **0.018** | * | * |
| ***Candida albicans*** | Mo CD273+ % of Monocytes | 0.143 | 0.441 | **-0.483** | **0.036** | | * |
| ***Candida albicans*** | Mo CD274+ % of Monocytes | -0.055 | 0.767 | **0.517** | **0.024** | | * |
| ***Candida albicans*** | Naive B cells non-memory % of Lymphocytes B | **-0.388** | **0.028** | -0.032 | 0.887 | * | |
| ***Candida albicans*** | Non-maturated non act B cells % of all Lymphocytes | **0.450** | **0.010** | -0.147 | 0.502 | ** | |
| ***Candida albicans*** | Plasmablasts % of Lymphocytes B | 0.214 | 0.239 | **0.808** | **0.000** | | *** |
| ***Candida albicans*** | TD Tc % of Cytotoxic Lymphocytes T | **0.386** | **0.032** | -0.225 | 0.354 | * | |
| ***Candida albicans*** | Transitional % of Lymphocytes B | -0.204 | 0.262 | **0.665** | **0.001** | | *** |
| ***Candida albicans*** | Treg % of Regulatory Lymphocytes T | 0.139 | 0.456 | **0.510** | **0.026** | | * |
| ***Candida dubliniensis*** | CD8+ % of Lymphocytes T | 0.164 | 0.379 | **0.502** | **0.029** | | * |
| ***Candida dubliniensis*** | Mo CD273+ % of Monocytes | **-0.534** | **0.002** | 0.061 | 0.803 | ** | |
| ***Candida dubliniensis*** | Mo CD274+ % of Monocytes | **0.791** | **0.000** | -0.069 | 0.778 | *** | |
| ***Candida dubliniensis*** | T cells CD274+ % of all Lymphocytes | **0.443** | **0.013** | -0.084 | 0.733 | * | |
| ***Candida dubliniensis*** | T cells PD-1-/CD152- % of all Lymphocytes | 0.156 | 0.401 | **0.462** | **0.046** | | * |
| ***Candida dubliniensis*** | T cells PD-1+ % of all Lymphocytes | -0.146 | 0.432 | **-0.471** | **0.042** | | * |
| ***Candida dubliniensis*** | Th CD185+ % of Regulatory Lymphocytes T | 0.009 | 0.962 | **0.575** | **0.010** | | ** |
| ***Candida dubliniensis*** | Treg % of Regulatory Lymphocytes T | 0.291 | 0.113 | **0.458** | **0.049** | | * |
| ***Candida glabrata*** | CD4-/CD8- % of Lymphocytes T | **0.360** | **0.046** | -0.120 | 0.624 | * | |
| ***Candida glabrata*** | CD4+/CD8+ % of Lymphocytes T | -0.077 | 0.681 | **0.584** | **0.009** | | ** |
| ***Candida glabrata*** | Switched post-germ center % of memory switched Lymphocytes B | **-0.599** | **0.000** | 0.066 | 0.771 | *** | |
| ***Candida glabrata*** | TDM Th % of Regulatory Lymphocytes T | **0.373** | **0.039** | 0.195 | 0.424 | * | |
| ***Debaryomyces hansenii*** | B cells % of all Lymphocytes | 0.125 | 0.497 | **0.536** | **0.008** | | ** |
| ***Debaryomyces hansenii*** | B cells % of PBMCs | 0.180 | 0.325 | **0.574** | **0.004** | | ** |
| ***Debaryomyces hansenii*** | Memory non-switched % of Lymphocytes B | -0.225 | 0.215 | **0.505** | **0.014** | | * |
| ***Debaryomyces hansenii*** | Mo CD273+/CD274+ % of Monocytes | **0.438** | **0.014** | -0.089 | 0.718 | * | |
| ***Malassezia restricta*** | B cells % of all Lymphocytes | **0.370** | **0.037** | 0.116 | 0.597 | * | |
| ***Malassezia restricta*** | Naive Th % of Regulatory Lymphocytes T | **0.485** | **0.006** | 0.083 | 0.735 | ** | |
| ***Malassezia restricta*** | Non-maturated act B cells % of all Lymphocytes | **0.384** | **0.030** | 0.073 | 0.739 | * | |

| | | | | | | | |
|---|---|---|---|---|---|---|---|
| ***Malassezia restricta*** | Post-germ center B cells % of memory switched Lymphocytes B | **0.398** | **0.024** | -0.164 | 0.466 | * | |
| ***Malassezia restricta*** | RTE % of Regulatory Lymphocytes T | **0.562** | **0.001** | 0.024 | 0.924 | ** | |
| ***Malassezia restricta*** | T cells CD273-/CD274- % of all Lymphocytes | **-0.486** | **0.006** | 0.149 | 0.541 | ** | |
| ***Malassezia restricta*** | T cells CD273+ % of all Lymphocytes | **0.490** | **0.005** | -0.139 | 0.569 | ** | |
| ***Saccharomyces cerevisiae*** | CD4-/CD8- % of Lymphocytes T | 0.130 | 0.486 | **0.606** | **0.006** | | ** |
| ***Saccharomyces cerevisiae*** | Mo CD273+ % of Monocytes | 0.022 | 0.906 | **-0.529** | **0.020** | | * |
| ***Saccharomyces cerevisiae*** | Naive Tc % of Cytotoxic Lymphocytes T | **0.379** | **0.036** | -0.379 | 0.109 | * | |
| ***Sporisorium graminicola*** | Late memory % of Lymphocytes B | **0.399** | **0.024** | -0.042 | 0.849 | * | |
| ***Sporisorium graminicola*** | RTE % of Regulatory Lymphocytes T | -0.053 | 0.778 | **0.537** | **0.018** | | * |
| ***Sporisorium graminicola*** | T cells % of all Lymphocytes | 0.036 | 0.848 | **-0.458** | **0.048** | | * |
| ***Sporisorium graminicola*** | T cells PD-1-/CD152- % of all Lymphocytes | -0.063 | 0.737 | **-0.513** | **0.025** | | * |
| ***Sporisorium graminicola*** | T cells PD-1+ % of all Lymphocytes | 0.066 | 0.724 | **0.505** | **0.027** | | * |
| ***Sporisorium graminicola*** | Transitional % of Lymphocytes B | **0.464** | **0.007** | -0.009 | 0.966 | ** | |
| ***Yarrowia lipolytica*** | B reg Transitional % of Lymphocytes B | 0.110 | 0.549 | **-0.436** | **0.038** | | * |
| ***Yarrowia lipolytica*** | Memory switched % of Lymphocytes B | -0.122 | 0.505 | **0.423** | **0.044** | | * |
| ***Yarrowia lipolytica*** | Naive B cells non-memory % of Lymphocytes B | -0.020 | 0.912 | **-0.428** | **0.041** | | * |
| ***Yarrowia lipolytica*** | Non-maturated non act B cells % of all Lymphocytes | 0.186 | 0.308 | **0.544** | **0.007** | | ** |
| ***Yarrowia lipolytica*** | Plasmacytes % of Lymphocytes B | **0.561** | **0.001** | -0.219 | 0.315 | *** | |
| ***Yarrowia lipolytica*** | T cells CD273+/CD274+ % of all Lymphocytes | **0.547** | **0.001** | -0.094 | 0.703 | ** | |
| ***Yarrowia lipolytica*** | T cells CD274+ % of all Lymphocytes | **0.367** | **0.042** | -0.108 | 0.660 | * | |
| ***Yarrowia lipolytica*** | T cells PD-1-/CD152- % of all Lymphocytes | **-0.478** | **0.006** | -0.043 | 0.862 | ** | |
| ***Yarrowia lipolytica*** | T cells PD-1+ % of all Lymphocytes | **0.490** | **0.005** | 0.062 | 0.801 | ** | |
| ***Yarrowia lipolytica*** | Treg % of Regulatory Lymphocytes T | **0.425** | **0.017** | **0.610** | **0.006** | * | ** |

Statistically significant correlations were marked in bold. 0.001***, 0.01**, 0.05*

**Table S6. Results of correlation analysis between gut fungi detected in analyzed samples and different types of lymphocytes circulating in the blood of melanoma patients in R (n=28) and NR (n=33) groups.** Only samples from the BT group were analyzed. Pearson correlation was performed, p<0.05.

| Fungi species | Lymphocytes | corr coef R | pval R | corr coef NR | pval NR | signif R | signif NR |
|---|---|---|---|---|---|---|---|
| ***Aspergillus fumigatus*** | Mo PD-1+ % of Monocytes | 0.085 | 0.701 | **-0.543** | **0.003** | | ** |
| ***Aspergillus fumigatus*** | T cells % of all Lymphocytes | 0.138 | 0.529 | **-0.403** | **0.037** | | * |
| ***Candida albicans*** | CD4-/CD8- % of Lymphocytes T | **0.474** | **0.022** | -0.015 | 0.943 | * | |
| ***Candida albicans*** | EM Th % of Regulatory Lymphocytes T | 0.329 | 0.125 | **0.406** | **0.036** | | * |
| ***Candida albicans*** | Late memory % of Lymphocytes B | -0.054 | 0.800 | **0.493** | **0.005** | | ** |
| ***Candida albicans*** | Plasmablasts % of Lymphocytes B | 0.329 | 0.116 | **0.553** | **0.001** | | ** |
| ***Candida albicans*** | Switched post-germ center % of memory switched Lymphocytes B | **-0.761** | **0.000** | 0.174 | 0.359 | *** | |
| ***Candida albicans*** | TDM Th % of Regulatory Lymphocytes T | **0.473** | **0.023** | -0.045 | 0.824 | * | |
| ***Candida albicans*** | Transitional % of Lymphocytes B | -0.191 | 0.370 | **0.365** | **0.044** | | * |
| ***Candida dubliniensis*** | Mo CD273+ % of Monocytes | 0.118 | 0.593 | **-0.398** | **0.040** | | * |
| ***Candida dubliniensis*** | Mo CD274+ % of Monocytes | -0.142 | 0.519 | **0.682** | **0.000** | | *** |
| ***Candida dubliniensis*** | Treg % of Regulatory Lymphocytes T | -0.098 | 0.658 | **0.465** | **0.014** | | * |
| ***Candida glabrata*** | CD4-/CD8- % of Lymphocytes T | **0.460** | **0.027** | -0.108 | 0.592 | * | |
| ***Candida glabrata*** | CD4+/CD8+ % of Lymphocytes T | -0.087 | 0.695 | **0.524** | **0.005** | | ** |
| ***Candida glabrata*** | Switched post-germ center % of memory switched Lymphocytes B | **-0.720** | **0.000** | 0.048 | 0.799 | *** | |
| ***Candida glabrata*** | TD Tc % of Cytotoxic Lymphocytes T | **0.436** | **0.037** | 0.118 | 0.559 | * | |
| ***Candida glabrata*** | TDM Th % of Regulatory Lymphocytes T | **0.436** | **0.038** | 0.110 | 0.584 | * | |
| ***Debaryomyces hansenii*** | B cells % of all Lymphocytes | 0.142 | 0.507 | **0.452** | **0.011** | | * |
| ***Debaryomyces hansenii*** | B cells % of PBMCs | 0.239 | 0.261 | **0.431** | **0.016** | | * |
| ***Debaryomyces hansenii*** | Mo CD273+/CD274+ % of Monocytes | **0.456** | **0.029** | -0.060 | 0.765 | * | |
| ***Debaryomyces hansenii*** | Mo CD274+ % of Monocytes | **0.625** | **0.001** | -0.061 | 0.763 | ** | |
| ***Malassezia restricta*** | Naive Th % of Regulatory Lymphocytes T | **0.445** | **0.033** | 0.215 | 0.281 | * | |
| ***Malassezia restricta*** | Non-maturated act B cells % of all Lymphocytes | **0.611** | **0.002** | 0.028 | 0.880 | ** | |
| ***Malassezia restricta*** | RTE % of Regulatory Lymphocytes T | **0.587** | **0.003** | 0.126 | 0.531 | ** | |
| ***Saccharomyces cerevisiae*** | CD4-/CD8- % of Lymphocytes T | 0.175 | 0.424 | **0.454** | **0.017** | | * |
| ***Saccharomyces cerevisiae*** | CD4+ % of Lymphocytes T | -0.193 | 0.377 | **-0.387** | **0.046** | | * |
| ***Sporisorium graminicola*** | Transitional % of Lymphocytes B | **0.754** | **0.000** | -0.088 | 0.637 | *** | |
| ***Yarrowia lipolytica*** | B reg Transitional % of Lymphocytes B | 0.091 | 0.673 | **-0.355** | **0.050** | | * |
| ***Yarrowia lipolytica*** | CD8+ % of Lymphocytes T | **0.421** | **0.046** | 0.256 | 0.197 | * | |
| ***Yarrowia lipolytica*** | Memory switched % of Lymphocytes B | -0.155 | 0.469 | **0.421** | **0.018** | | * |
| ***Yarrowia lipolytica*** | Naive B cells non-memory % of Lymphocytes B | -0.039 | 0.856 | **-0.364** | **0.044** | | * |
| ***Yarrowia lipolytica*** | Non-maturated non act B cells % of all Lymphocytes | 0.232 | 0.275 | **0.415** | **0.020** | | * |
| ***Yarrowia lipolytica*** | Plasmacytes % of Lymphocytes B | **0.570** | **0.004** | -0.196 | 0.291 | ** | |
| ***Yarrowia lipolytica*** | T cells CD273+/CD274+ % of all Lymphocytes | **0.566** | **0.005** | -0.035 | 0.862 | ** | |

| | | | | | | | |
|---|---|---|---|---|---|---|---|
| ***Yarrowia lipolytica*** | T cells CD274+ % of all Lymphocytes | **0.474** | **0.022** | -0.073 | 0.718 | * | |
| ***Yarrowia lipolytica*** | T cells PD-1-/CD152- % of all Lymphocytes | **-0.515** | **0.012** | -0.006 | 0.977 | * | |
| ***Yarrowia lipolytica*** | T cells PD-1+ % of all Lymphocytes | **0.525** | **0.010** | 0.023 | 0.910 | * | |
| ***Yarrowia lipolytica*** | Treg % of Regulatory Lymphocytes T | **0.535** | **0.009** | **0.518** | **0.006** | ** | ** |

Statistically significant correlations were marked in **bold**. 0.001***, 0.01**, 0.05*

**Table S7. Results of paired samples correlation analysis between gut fungi detected in analyzed samples and different types of lymphocytes circulating in the blood of melanoma patients for CB (n=28) and NB (n=8) groups.** Pearson correlation was used for testing the correlation between paired samples, $p<0.05$. Paired samples from the BT (n=36) and T3 (n=36) groups were analyzed. A correlation analysis was performed between the difference in counts for each analyzed fungal species in BT and T3 and the change in the number of each analyzed lymphocyte type between BT and T3. The difference in counts for each fungal species was obtained by subtracting the fungal species count before the treatment (BT) from the count in the third month of the treatment (T3) for all matched samples. Similarly, the change in the number of lymphocytes was obtained by subtracting the number of a given lymphocyte type before the treatment (BT) from the number in the third month of the treatment (T3) for all matched samples.

| Fungi species | Lymphocytes | corr coef CB | pval CB | corr coef NB | pval NB | signif CB | signif NB |
|---|---|---|---|---|---|---|---|
| ***Aspergillus fumigatus*** | T cells CD273+ % of all Lymphocytes | **-0.5250** | **0.0101** | | | * | |
| ***Aspergillus fumigatus*** | T cells CD273-/CD274- % of all Lymphocytes | **0.5173** | **0.0115** | | | * | |
| ***Aspergillus fumigatus*** | Mo CD274+ % of Monocytes | **-0.4649** | **0.0254** | | | * | |
| ***Aspergillus fumigatus*** | Th CD185+ % of Regulatory Lymphocytes T | **-0.4622** | **0.0264** | | | * | |
| ***Aspergillus oryzae*** | T cells CD274+ % of all Lymphocytes | 0.0081 | 0.9707 | **0.9995** | **0.0000** | | *** |
| ***Aspergillus oryzae*** | T cells CD273+/CD274+ % of all Lymphocytes | 0.0363 | 0.8695 | **0.9954** | **0.0000** | | *** |
| ***Aspergillus oryzae*** | Non-maturated act B cells % of all Lymphocytes | -0.1444 | 0.5111 | **0.9905** | **0.0001** | | *** |
| ***Aspergillus oryzae*** | Plasmacytes % of Lymphocytes B | -0.0472 | 0.8307 | **0.9457** | **0.0043** | | ** |
| ***Aspergillus oryzae*** | T cells CD273-/CD274- % of all Lymphocytes | 0.0363 | 0.8694 | **-0.9328** | **0.0066** | | ** |
| ***Aspergillus oryzae*** | CD4-/CD8- % of Lymphocytes T | -0.0424 | 0.8476 | **0.9121** | **0.0112** | | * |
| ***Aspergillus oryzae*** | B cells % of PBMCs | -0.0035 | 0.9874 | **0.8956** | **0.0158** | | * |
| ***Aspergillus oryzae*** | Naive B cells non-memory % of Lymphocytes B | 0.0472 | 0.8307 | **-0.8947** | **0.0161** | | * |
| ***Aspergillus oryzae*** | T cells CD273+ % of all Lymphocytes | -0.0345 | 0.8760 | **0.8814** | **0.0203** | | * |
| ***Aspergillus oryzae*** | Th CD185+ % of Regulatory Lymphocytes T | -0.0548 | 0.8038 | **0.8621** | **0.0272** | | * |
| ***Aspergillus oryzae*** | B cells % of all Lymphocytes | -0.1024 | 0.6418 | **0.8457** | **0.0339** | | * |
| ***Candida albicans*** | T cells PD-1+/CD152+ % of all Lymphocytes | -0.0670 | 0.7615 | **-0.9986** | **0.0000** | | *** |
| ***Candida albicans*** | T cells % of all Lymphocytes | -0.0799 | 0.7172 | **-0.9151** | **0.0105** | | * |
| ***Candida albicans*** | Mo CD273+/CD274+ % of Monocytes | -0.0379 | 0.8636 | **-0.9048** | **0.0132** | | * |
| ***Candida albicans*** | T cells PD-1-/CD152- % of all Lymphocytes | 0.2018 | 0.3557 | **0.8824** | **0.0199** | | * |
| ***Candida albicans*** | T cells PD-1+ % of all Lymphocytes | -0.2112 | 0.3335 | **-0.8718** | **0.0236** | | * |
| ***Candida albicans*** | Mo CD273+ % of Monocytes | -0.0655 | 0.7666 | **0.8636** | **0.0266** | | * |
| ***Candida albicans*** | B reg transitional % of Lymphocytes B | -0.1672 | 0.4457 | **0.8569** | **0.0293** | | * |
| ***Candida albicans*** | Tc CD185+ % of Cytotoxic Lymphocytes T | 0.0490 | 0.8242 | **-0.8344** | **0.0389** | | * |
| ***Candida albicans*** | B cells % of all Lymphocytes | **0.5334** | **0.0088** | -0.7466 | 0.0882 | ** | . |
| ***Candida albicans*** | B cells % of PBMCs | **0.5141** | **0.0121** | -0.6730 | 0.1429 | * | |
| ***Candida albicans*** | Non-maturated non act B cells % of Lymphocytes B | **0.4256** | **0.0429** | -0.5673 | 0.2403 | * | |
| ***Candida albicans*** | Switched post-germ center % of memory switched Lymphocytes B | **-0.8156** | **0.0000** | 0.4464 | 0.3749 | *** | |
| ***Candida albicans*** | Memory non-switched % of Lymphocytes B | **0.4479** | **0.0321** | -0.3912 | 0.4431 | * | |

| | | | | | | | |
|---|---|---|---|---|---|---|---|
| ***Candida albicans*** | Memory switched % of Lymphocytes B | **-0.4226** | **0.0445** | 0.0986 | 0.8526 | * | |
| ***Candida albicans*** | Plasmablasts % of Lymphocytes B | **0.4853** | **0.0189** | 0.0869 | 0.8699 | * | |
| ***Candida dubliniensis*** | Mo PD-1+ % of Monocytes | -0.1215 | 0.5808 | **0.9377** | **0.0057** | | ** |
| ***Candida dubliniensis*** | CD4+/CD8+ % of Lymphocytes T | -0.1879 | 0.3905 | **-0.8855** | **0.0189** | | * |
| ***Candida dubliniensis*** | EM Th % of Regulatory Lymphocytes T | **0.4208** | **0.0456** | **-0.8378** | **0.0373** | * | * |
| ***Candida dubliniensis*** | EM Tc % of Cytotoxic Lymphocytes T | 0.2015 | 0.3564 | **-0.8202** | **0.0456** | | * |
| ***Candida dubliniensis*** | Treg % of Regulatory Lymphocytes T | **-0.4727** | **0.0227** | 0.7040 | 0.1185 | * | |
| ***Candida dubliniensis*** | Mo CD274+ % of Monocytes | **-0.4799** | **0.0205** | -0.3050 | 0.5567 | * | |
| ***Candida dubliniensis*** | CD8+ % of Lymphocytes T | **0.8485** | **0.0000** | 0.2808 | 0.5898 | *** | |
| ***Candida dubliniensis*** | T cells CD152+ % of all Lymphocytes | **0.4639** | **0.0258** | 0.2346 | 0.6546 | * | |
| ***Candida dubliniensis*** | CM Tc % of Cytotoxic Lymphocytes T | **-0.6372** | **0.0011** | -0.2030 | 0.6997 | ** | |
| ***Candida dubliniensis*** | B cells % of all Lymphocytes | **0.4360** | **0.0376** | -0.1737 | 0.7421 | * | |
| ***Candida dubliniensis*** | T cells CD273+/CD274+ % of all Lymphocytes | **0.4325** | **0.0393** | -0.0729 | 0.8908 | * | |
| ***Candida dubliniensis*** | Mo CD273+/CD274+ % of Monocytes | **0.7124** | **0.0001** | 0.0121 | 0.9819 | *** | |
| ***Candida dubliniensis*** | CD4+ % of Lymphocytes T | **-0.7862** | **0.0000** | 0.0024 | 0.9964 | *** | |
| ***Candida glabrata*** | CD4+/CD8+ % of Lymphocytes T | 0.0804 | 0.7155 | **-0.8862** | **0.0187** | | * |
| ***Candida glabrata*** | Mo PD-1+ % of Monocytes | -0.1407 | 0.5218 | **0.8349** | **0.0386** | | * |
| ***Candida glabrata*** | EM Tc % of Cytotoxic Lymphocytes T | -0.0339 | 0.8781 | **-0.8248** | **0.0434** | | * |
| ***Candida glabrata*** | Memory switched % of Lymphocytes B | **-0.4367** | **0.0372** | 0.7325 | 0.0978 | * | . |
| ***Candida glabrata*** | Non-maturated non act B cells % of Lymphocytes B | **0.4600** | **0.0272** | -0.6490 | 0.1632 | * | |
| ***Candida glabrata*** | Memory non-switched % of Lymphocytes B | **0.4544** | **0.0294** | -0.5899 | 0.2178 | * | |
| ***Candida glabrata*** | Switched post-germ center % of memory switched Lymphocytes B | **-0.7935** | **0.0000** | 0.2786 | 0.5929 | *** | |
| ***Candida glabrata*** | B cells % of PBMCs | **0.5003** | **0.0150** | -0.2431 | 0.6425 | * | |
| ***Candida glabrata*** | B cells % of all Lymphocytes | **0.4997** | **0.0152** | -0.1974 | 0.7078 | * | |
| ***Candida glabrata*** | Plasmablasts % of Lymphocytes B | **0.5345** | **0.0086** | -0.1063 | 0.8411 | ** | |
| ***Debaryomyces hansenii*** | Post-germ center B cells % of memory switched Lymphocytes B | -0.0978 | 0.6570 | **0.8555** | **0.0298** | | * |
| ***Debaryomyces hansenii*** | Switched post-germ center % of memory switched Lymphocytes B | 0.1054 | 0.6321 | **-0.8538** | **0.0305** | | * |
| ***Debaryomyces hansenii*** | CM Tc % of Cytotoxic Lymphocytes T | **0.4137** | **0.0497** | -0.6667 | 0.1481 | * | |
| ***Debaryomyces hansenii*** | Treg % of Regulatory Lymphocytes T | **-0.4222** | **0.0448** | -0.1121 | 0.8325 | * | |
| ***Kluyveromyces lactis*** | Mo CD273+/CD274+ % of Monocytes | | | **0.0034** | | ** | |
| ***Kluyveromyces lactis*** | B reg transitional % of Lymphocytes B | | | **0.0060** | | ** | |
| ***Kluyveromyces lactis*** | T cells PD-1+/CD152+ % of all Lymphocytes | | | **0.0213** | | * | |
| ***Kluyveromyces lactis*** | T cells % of all Lymphocytes | | | **0.8477** | **0.0330** | | * |
| ***Kluyveromyces lactis*** | Mo CD273+ % of Monocytes | | | **-0.8456** | **0.0339** | | * |
| ***Malassezia restricta*** | T cells PD-1+/CD152+ % of all Lymphocytes | **0.2593** | 0.2322 | **0.9258** | **0.0080** | | ** |
| ***Malassezia restricta*** | Treg % of Regulatory Lymphocytes T | 0.0718 | 0.7449 | **-0.8904** | **0.0174** | | * |

| | | | | | | | |
|---|---|---|---|---|---|---|---|
| ***Malassezia restricta*** | T cells PD-1-/CD152- % of all Lymphocytes | 0.0626 | 0.7766 | **-0.8801** | **0.0207** | | * |
| ***Malassezia restricta*** | T cells PD-1+ % of all Lymphocytes | -0.0828 | 0.7073 | **0.8787** | **0.0212** | | * |
| ***Malassezia restricta*** | T cells % of all Lymphocytes | **-0.4945** | **0.0165** | **0.8149** | **0.0482** | * | * |
| ***Malassezia restricta*** | CD4+/CD8+ % of Lymphocytes T | **0.4452** | **0.0333** | 0.2846 | 0.5846 | * | |
| ***Malassezia restricta*** | CD4-/CD8- % of Lymphocytes T | **0.4214** | **0.0452** | 0.0307 | 0.9540 | * | |
| ***Neurospora crassa*** | CD4+/CD8+ % of Lymphocytes T | -0.0457 | 0.8360 | **-0.8862** | **0.0187** | | * |
| ***Neurospora crassa*** | Mo PD-1+ % of Monocytes | 0.3291 | 0.1252 | **0.8349** | **0.0386** | | * |
| ***Neurospora crassa*** | EM Tc % of Cytotoxic Lymphocytes T | 0.0324 | 0.8832 | **-0.8248** | **0.0434** | | * |
| ***Neurospora crassa*** | CD27+ Memory % of Lymphocytes B | **0.4360** | **0.0376** | -0.4622 | 0.3560 | * | |
| ***Neurospora crassa*** | CD27- Non-memory % of Lymphocytes B | **-0.4360** | **0.0376** | 0.4622 | 0.3560 | * | |
| ***Saccharomyces cerevisiae*** | T cells CD274+ % of all Lymphocytes | 0.1159 | 0.5986 | **-0.9395** | **0.0054** | | ** |
| ***Saccharomyces cerevisiae*** | T cells CD273+/CD274+ % of all Lymphocytes | 0.3494 | 0.1022 | **-0.9338** | **0.0064** | | ** |
| ***Saccharomyces cerevisiae*** | Non-maturated act B cells % of all Lymphocytes | 0.0035 | 0.9874 | **-0.9078** | **0.0124** | | * |
| ***Saccharomyces cerevisiae*** | T cells CD273-/CD274- % of all Lymphocytes | -0.2086 | 0.3395 | **0.8872** | **0.0184** | | * |
| ***Saccharomyces cerevisiae*** | T cells CD273+ % of all Lymphocytes | 0.2021 | 0.3551 | **-0.8422** | **0.0354** | | * |
| ***Saccharomyces cerevisiae*** | Plasmacytes % of Lymphocytes B | 0.0926 | 0.6742 | **-0.8412** | **0.0358** | | * |
| ***Saccharomyces cerevisiae*** | CD4-/CD8- % of Lymphocytes T | 0.2719 | 0.2095 | **-0.8124** | **0.0495** | | * |
| ***Saccharomyces cerevisiae*** | Late memory % of Lymphocytes B | 0.1117 | 0.6120 | **-0.8122** | **0.0496** | | * |
| ***Scheffersomyces stipitis*** | Post-germ center B cells % of memory switched Lymphocytes B | 0.0524 | 0.8122 | **0.8608** | **0.0277** | | * |
| ***Scheffersomyces stipitis*** | Switched post-germ center % of memory switched Lymphocytes B | -0.0096 | 0.9654 | **-0.8600** | **0.0280** | | * |
| ***Scheffersomyces stipitis*** | TD Tc % of Cytotoxic Lymphocytes T | **-0.7424** | **0.0000** | 0.5877 | 0.2199 | *** | |
| ***Scheffersomyces stipitis*** | EM Th % of Regulatory Lymphocytes T | **0.4388** | **0.0362** | -0.2882 | 0.5796 | * | |
| ***Scheffersomyces stipitis*** | T cells % of all PBMCs | **0.4608** | **0.0269** | -0.2685 | 0.6070 | * | |
| ***Scheffersomyces stipitis*** | EM Tc % of Cytotoxic Lymphocytes T | **0.6043** | **0.0023** | 0.1594 | 0.7629 | ** | |
| ***Scheffersomyces stipitis*** | B cells % of all Lymphocytes | **-0.4397** | **0.0358** | 0.0726 | 0.8913 | * | |
| ***Scheffersomyces stipitis*** | CD4+/CD8+ % of Lymphocytes T | **0.5824** | **0.0035** | 0.0692 | 0.8963 | ** | |
| ***Scheffersomyces stipitis*** | T cells PD-1+ % of all Lymphocytes | **0.4501** | **0.0311** | -0.0660 | 0.9011 | * | |
| ***Scheffersomyces stipitis*** | T cells PD-1-/CD152- % of all Lymphocytes | **-0.4305** | **0.0403** | 0.0585 | 0.9124 | * | |
| ***Sporisorium graminicola*** | CD4+ % of Lymphocytes T | 0.1626 | 0.4586 | **0.8823** | **0.0200** | | * |
| ***Sporisorium graminicola*** | Plasmablasts % of Lymphocytes B | 0.1480 | 0.5004 | **-0.8784** | **0.0213** | | * |
| ***Sporisorium graminicola*** | Tc CD185+ % of Cytotoxic Lymphocytes T | **-0.4277** | **0.0418** | 0.0460 | 0.9311 | * | |
| ***Sugiyamaella lignohabitans*** | CD4+/CD8+ % of Lymphocytes T | -0.1898 | 0.3857 | **-0.9117** | **0.0113** | | * |
| ***Sugiyamaella lignohabitans*** | Mo CD274+ % of Monocytes | -0.2014 | 0.3568 | **-0.8128** | **0.0493** | | * |
| ***Sugiyamaella lignohabitans*** | Transitional % of Lymphocytes B | **0.8334** | **0.0000** | -0.6721 | 0.1436 | *** | |
| ***Sugiyamaella lignohabitans*** | Mo PD-1+ % of Monocytes | **0.6221** | **0.0015** | 0.5831 | 0.2245 | ** | |

| | | | | | | | |
|---|---|---|---|---|---|---|---|
| ***Sugiyamaella lignohabitans*** | T cells % of all Lymphocytes | **0.5065** | **0.0136** | 0.4290 | 0.3960 | * | |
| ***Sugiyamaella lignohabitans*** | Tc CD185+ % of Cytotoxic Lymphocytes T | **-0.4617** | **0.0266** | 0.3946 | 0.4388 | * | |
| ***Talaromyces rugulosus*** | B reg memory % of Lymphocytes B | **0.5099** | **0.0129** | | | * | |
| ***Talaromyces rugulosus*** | Late memory % of Lymphocytes B | **-0.4190** | **0.0466** | | | * | |
| ***Ustilago maydis*** | Post-germ center B cells % of memory switched Lymphocytes B | 0.0876 | 0.6911 | **-0.8608** | **0.0277** | | * |
| ***Ustilago maydis*** | Switched post-germ center % of memory switched Lymphocytes B | -0.0176 | 0.9367 | **0.8600** | **0.0280** | | * |
| ***Ustilago maydis*** | TD Tc % of Cytotoxic Lymphocytes T | **-0.8536** | **0.0000** | -0.5877 | 0.2199 | *** | |
| ***Ustilago maydis*** | EM Th % of Regulatory Lymphocytes T | **0.5153** | **0.0118** | 0.2882 | 0.5796 | * | |
| ***Ustilago maydis*** | T cells % of all PBMCs | **0.5275** | **0.0097** | 0.2685 | 0.6070 | ** | |
| ***Ustilago maydis*** | EM Tc % of Cytotoxic Lymphocytes T | **0.7839** | **0.0000** | -0.1594 | 0.7629 | *** | |
| ***Ustilago maydis*** | CD4+/CD8+ % of Lymphocytes T | **0.5891** | **0.0031** | -0.0692 | 0.8963 | ** | |
| ***Ustilago maydis*** | T cells PD-1+ % of all Lymphocytes | **0.4231** | **0.0442** | 0.0660 | 0.9011 | * | |
| ***Yarrowia lipolytica*** | CD4+/CD8+ % of Lymphocytes T | **0.1520** | **0.4888** | **-0.9440** | **0.0046** | | ** |
| ***Yarrowia lipolytica*** | EM Tc % of Cytotoxic Lymphocytes T | -0.0079 | 0.9715 | **-0.8373** | **0.0376** | | * |
| ***Yarrowia lipolytica*** | Plasmacytes % of Lymphocytes B | **0.5881** | **0.0032** | -0.3914 | 0.4428 | ** | |
| ***Yarrowia lipolytica*** | T cells PD-1+ % of all Lymphocytes | **0.4654** | **0.0252** | -0.2446 | 0.6404 | * | |
| ***Yarrowia lipolytica*** | T cells PD-1-/CD152- % of all Lymphocytes | **-0.4510** | **0.0308** | 0.2245 | 0.6689 | * | |
| ***Yarrowia lipolytica*** | T cells CD274+ % of all Lymphocytes | **-0.4148** | **0.0490** | -0.1286 | 0.8081 | * | |
| ***Yarrowia lipolytica*** | Mo CD273+ % of Monocytes | **0.4318** | **0.0396** | -0.1220 | 0.8179 | * | |
| ***Yarrowia lipolytica*** | T cells CD273+/CD274+ % of all Lymphocytes | **0.5408** | **0.0077** | -0.0950 | 0.8579 | ** | |
| ***Zygosaccharomyces rouxii*** | CD4+/CD8+ % of Lymphocytes T | 0.1814 | 0.4076 | **0.8862** | **0.0187** | | * |
| ***Zygosaccharomyces rouxii*** | Mo PD-1+ % of Monocytes | **-0.6221** | **0.0015** | **-0.8349** | **0.0386** | ** | * |
| ***Zygosaccharomyces rouxii*** | EM Tc % of Cytotoxic Lymphocytes T | 0.0937 | 0.6705 | **0.8248** | **0.0434** | | * |
| ***Zygosaccharomyces rouxii*** | Transitional % of Lymphocytes B | **-0.8143** | **0.0000** | 0.2473 | 0.6366 | *** | |
| ***Zygosaccharomyces rouxii*** | T cells % of all Lymphocytes | **-0.4857** | **0.0188** | 0.1831 | 0.7285 | * | |
| ***Zygosaccharomyces rouxii*** | Tc CD185+ % of Cytotoxic Lymphocytes T | **0.4707** | **0.0234** | 0.1578 | 0.7652 | * | |
| ***Zygotorulaspora mrakii*** | Transitional % of Lymphocytes B | **-0.7990** | **0.0000** | | | *** | |
| ***Zygotorulaspora mrakii*** | Mo PD-1+ % of Monocytes | **-0.5859** | **0.0033** | | | ** | |
| ***Zygotorulaspora mrakii*** | Naive Tc % of Cytotoxic Lymphocytes T | **-0.4905** | **0.0175** | | | * | |
| ***Zygotorulaspora mrakii*** | T cells % of all Lymphocytes | **-0.4625** | **0.0263** | | | * | |

Statistically significant correlations were marked in **bold**. 0.001***, 0.01**, 0.05*

**Table S8. Results of paired samples correlation analysis between gut fungi detected in analyzed samples and different types of lymphocytes circulating in the blood of melanoma patients for R (n=22) and NR (n=14) groups.** Pearson correlation was used for testing the correlation between paired samples, p<0.05. Paired samples from the BT (n=36) and T3 (n=36) groups were analyzed. A correlation analysis was performed between the difference in counts for each analyzed fungal species in BT and T3 and the change in the number of each analyzed lymphocyte type between BT and T3. The difference in counts for each fungal species was obtained by subtracting the fungal species count before the treatment (BT) from the count in the third month of the treatment (T3) for all matched samples. Similarly, the change in the number of lymphocytes was obtained by subtracting the number of a given lymphocyte type before the treatment (BT) from the number in the third month of the treatment (T3) for all matched samples.

| Fungi species | Lymphocytes | corr coef R | pval R | corr coef NR | pval NR | signif R | signif NR |
|---|---|---|---|---|---|---|---|
| ***Aspergillus fumigatus*** | Mo CD274+ % of Monocytes | **-0.6565** | **0.0031** | 0.0820 | 0.8106 | ** | |
| ***Aspergillus fumigatus*** | T cells CD273+ % of all Lymphocytes | **-0.5763** | **0.0123** | -0.2576 | 0.4445 | * | |
| ***Aspergillus fumigatus*** | T cells CD273-/CD274- % of all Lymphocytes | **0.5666** | **0.0142** | 0.2300 | 0.4962 | * | |
| ***Aspergillus fumigatus*** | Th CD185+ % of Regulatory Lymphocytes T | **-0.5124** | **0.0297** | -0.1026 | 0.7640 | * | |
| ***Aspergillus fumigatus*** | CD8+ % of Lymphocytes T | 0.1101 | 0.6638 | **-0.8779** | **0.0004** | | *** |
| ***Aspergillus fumigatus*** | EM Th % of Regulatory Lymphocytes T | 0.3579 | 0.1447 | **-0.7559** | **0.0071** | | ** |
| ***Aspergillus fumigatus*** | CD4+ % of Lymphocytes T | -0.0242 | 0.9241 | **0.8435** | **0.0011** | | ** |
| ***Aspergillus fumigatus*** | T cells CD152+ % of all Lymphocytes | 0.0031 | 0.9901 | **-0.7941** | **0.0035** | | ** |
| ***Aspergillus fumigatus*** | Treg % of Regulatory Lymphocytes T | -0.1016 | 0.6884 | **0.6194** | **0.0421** | | * |
| ***Aspergillus fumigatus*** | CM Tc % of Cytotoxic Lymphocytes T | -0.0705 | 0.7812 | **0.7116** | **0.0140** | | * |
| ***Aspergillus oryzae*** | T cells CD273-/CD274- % of all Lymphocytes | 0.0669 | 0.7918 | **-0.7466** | **0.0083** | | ** |
| ***Aspergillus oryzae*** | T cells CD274+ % of all Lymphocytes | 0.0083 | 0.9739 | **0.7387** | **0.0094** | | ** |
| ***Aspergillus oryzae*** | Late memory % of Lymphocytes B | 0.1891 | 0.4522 | **0.6623** | **0.0264** | | * |
| ***Aspergillus oryzae*** | T cells CD273+ % of all Lymphocytes | -0.0641 | 0.8005 | **0.7126** | **0.0139** | | * |
| ***Aspergillus oryzae*** | T cells CD273+/CD274+ % of all Lymphocytes | 0.0267 | 0.9161 | **0.6929** | **0.0181** | | * |
| ***Candida albicans*** | Switched post-germ center % of memory switched Lymphocytes B | **-0.8475** | **0.0000** | -0.0504 | 0.8829 | *** | |
| ***Candida albicans*** | Non-maturated act B cells % of all Lymphocytes | **0.6576** | **0.0030** | -0.0769 | 0.8222 | ** | |
| ***Candida albicans*** | B cells % of all Lymphocytes | **0.5932** | **0.0095** | -0.3267 | 0.3267 | ** | |
| ***Candida albicans*** | B cells % of PBMCs | **0.5899** | **0.0100** | -0.2345 | 0.4877 | ** | |
| ***Candida albicans*** | TDM Th % of Regulatory Lymphocytes T | **-0.4943** | **0.0370** | **0.6940** | **0.0178** | * | * |
| ***Candida albicans*** | Memory non-switched % of Lymphocytes B | **0.5618** | **0.0153** | -0.1380 | 0.6857 | * | |
| ***Candida albicans*** | Plasmablasts % of Lymphocytes B | **0.5295** | **0.0238** | -0.0509 | 0.8818 | * | |
| ***Candida albicans*** | Non-maturated non act B cells % of Lymphocytes B | **0.5247** | **0.0254** | -0.2711 | 0.4201 | * | |
| ***Candida albicans*** | CD27- Non-memory % of Lymphocytes B | **-0.5176** | **0.0278** | 0.1228 | 0.7190 | * | |
| ***Candida albicans*** | CD27+ Memory % of Lymphocytes B | **0.5176** | **0.0278** | -0.1228 | 0.7190 | * | |
| ***Candida albicans*** | Naive B cells non-memory % of Lymphocytes B | **-0.4897** | **0.0391** | 0.0554 | 0.8715 | * | |
| ***Candida albicans*** | T cells PD-1+/CD152+ % of all Lymphocytes | -0.0075 | 0.9765 | **-0.8597** | **0.0007** | | *** |
| ***Candida albicans*** | T cells PD-1+ % of all Lymphocytes | -0.2013 | 0.4232 | **-0.6873** | **0.0195** | | * |
| ***Candida albicans*** | T cells PD-1-/CD152- % of all Lymphocytes | 0.1892 | 0.4522 | **0.6829** | **0.0206** | | * |
| ***Candida dubliniensis*** | B cells % of PBMCs | **0.7212** | **0.0007** | 0.2441 | 0.4695 | *** | |
| ***Candida dubliniensis*** | Switched post-germ center % of memory switched Lymphocytes B | **-0.6964** | **0.0013** | -0.0152 | 0.9647 | ** | |

| | | | | | | | |
|---|---|---|---|---|---|---|---|
| ***Candida dubliniensis*** | Non-maturated non act B cells % of Lymphocytes B | **0.5990** | **0.0086** | 0.0256 | 0.9404 | ** | |
| ***Candida dubliniensis*** | Non-maturated act B cells % of all Lymphocytes | **0.5329** | **0.0228** | 0.2840 | 0.3974 | * | |
| ***Candida dubliniensis*** | Plasmablasts % of Lymphocytes B | **0.5261** | **0.0249** | -0.2414 | 0.4745 | * | |
| ***Candida dubliniensis*** | Memory non-switched % of Lymphocytes B | **0.4948** | **0.0368** | 0.1113 | 0.7446 | * | |
| ***Candida dubliniensis*** | CD8+ % of Lymphocytes T | -0.0710 | 0.7794 | **0.8800** | **0.0004** | | *** |
| ***Candida dubliniensis*** | CD4+ % of Lymphocytes T | 0.0822 | 0.7457 | **-0.8308** | **0.0015** | | ** |
| ***Candida dubliniensis*** | T cells CD152+ % of all Lymphocytes | -0.0350 | 0.8904 | **0.7905** | **0.0038** | | ** |
| ***Candida dubliniensis*** | CM Tc % of Cytotoxic Lymphocytes T | 0.0416 | 0.8697 | **-0.7158** | **0.0133** | | * |
| ***Candida dubliniensis*** | EM Th % of Regulatory Lymphocytes T | 0.0389 | 0.8783 | **0.6905** | **0.0187** | | * |
| ***Candida glabrata*** | Memory switched % of Lymphocytes B | -0.4287 | 0.0759 | **0.6908** | **0.0186** | . | * |
| ***Candida glabrata*** | Switched post-germ center % of memory switched Lymphocytes B | **-0.8253** | **0.0000** | 0.1743 | 0.6082 | *** | |
| ***Candida glabrata*** | Non-maturated act B cells % of all Lymphocytes | **0.6675** | **0.0025** | -0.1958 | 0.5640 | ** | |
| ***Candida glabrata*** | B cells % of PBMCs | **0.5957** | **0.0091** | -0.1910 | 0.5736 | ** | |
| ***Candida glabrata*** | Memory non-switched % of Lymphocytes B | **0.5774** | **0.0121** | -0.3027 | 0.3656 | * | |
| ***Candida glabrata*** | Non-maturated non act B cells % of Lymphocytes B | **0.5659** | **0.0144** | -0.5047 | 0.1133 | * | |
| ***Candida glabrata*** | B cells % of all Lymphocytes | **0.5626** | **0.0151** | -0.1504 | 0.6589 | * | |
| ***Candida glabrata*** | Plasmablasts % of Lymphocytes B | **0.5599** | **0.0157** | -0.1419 | 0.6774 | * | |
| ***Candida glabrata*** | CD27- Non-memory % of Lymphocytes B | **-0.5290** | **0.0240** | 0.2247 | 0.5065 | * | |
| ***Candida glabrata*** | CD27+ Memory % of Lymphocytes B | **0.5290** | **0.0240** | -0.2247 | 0.5065 | * | |
| ***Candida glabrata*** | Naive B cells non-memory % of Lymphocytes B | **-0.4969** | **0.0359** | 0.0165 | 0.9616 | * | |
| ***Candida glabrata*** | TDM Th % of Regulatory Lymphocytes T | **-0.4964** | **0.0361** | 0.0103 | 0.9759 | * | |
| ***Candida glabrata*** | CD4+/CD8+ % of Lymphocytes T | 0.0946 | 0.7089 | **-0.7950** | **0.0035** | | ** |
| ***Candida glabrata*** | Mo PD-1+ % of Monocytes | -0.1741 | 0.4896 | **0.6178** | **0.0428** | | * |
| ***Debaryomyces hansenii*** | CM Tc % of Cytotoxic Lymphocytes T | **0.6319** | **0.0049** | -0.3287 | 0.3237 | ** | |
| ***Debaryomyces hansenii*** | Post-germ center B cells % of memory switched Lymphocytes B | -0.0780 | 0.7584 | **0.8471** | **0.0010** | | *** |
| ***Debaryomyces hansenii*** | Switched post-germ center % of memory switched Lymphocytes B | 0.1038 | 0.6819 | **-0.7946** | **0.0035** | | ** |
| ***Kluyveromyces lactis*** | Mo CD273+/CD274+ % of Monocytes | | | **0.8726** | **0.0005** | | *** |
| ***Kluyveromyces lactis*** | B reg transitional % of Lymphocytes B | | | **-0.8131** | **0.0023** | | ** |
| ***Kluyveromyces lactis*** | Mo CD274+ % of Monocytes | | | **-0.8086** | **0.0026** | | ** |
| ***Kluyveromyces lactis*** | Mo CD273+ % of Monocytes | | | **-0.7200** | **0.0125** | | * |
| ***Kluyveromyces lactis*** | Tc CD185+ % of Cytotoxic Lymphocytes T | | | **0.7054** | **0.0153** | | * |
| ***Kluyveromyces lactis*** | Transitional % of Lymphocytes B | | | **-0.7014** | **0.0162** | | * |
| ***Malassezia restricta*** | CD4+/CD8+ % of Lymphocytes T | **0.4816** | **0.0430** | 0.2457 | 0.4665 | * | |
| ***Malassezia restricta*** | Mo PD-1+ % of Monocytes | -0.2231 | 0.3735 | **-0.6646** | **0.0257** | | * |
| ***Neurospora crassa*** | CD4+/CD8+ % of Lymphocytes T | | | **-0.7879** | **0.0040** | | ** |
| ***Neurospora crassa*** | Memory switched % of Lymphocytes B | | | **0.6885** | **0.0191** | | * |
| ***Neurospora crassa*** | Mo PD-1+ % of Monocytes | | | **0.6687** | **0.0245** | | * |
| ***Pichia kudriavzevii*** | CD27+ Memory % of Lymphocytes B | **0.6888** | **0.0016** | -0.4945 | 0.1221 | ** | |
| ***Pichia kudriavzevii*** | CD27- Non-memory % of Lymphocytes B | **-0.6888** | **0.0016** | 0.4945 | 0.1221 | ** | |
| ***Pichia kudriavzevii*** | Naive B cells non-memory % of Lymphocytes B | **-0.6637** | **0.0027** | 0.3544 | 0.2850 | ** | |
| ***Pichia kudriavzevii*** | RTE % of Regulatory Lymphocytes T | **0.6130** | **0.0068** | 0.2332 | 0.4902 | ** | |
| ***Pichia kudriavzevii*** | Memory non-switched % of Lymphocytes B | **0.5667** | **0.0142** | -0.4993 | 0.1179 | * | |

| | | | | | | | |
|---|---|---|---|---|---|---|---|
| *Scheffersomyces stipitis* | TD Tc % of Cytotoxic Lymphocytes T | **-0.9066** | **0.0000** | **0.6018** | **0.0501** | *** | . |
| *Scheffersomyces stipitis* | EM Tc % of Cytotoxic Lymphocytes T | **0.8931** | **0.0000** | -0.2789 | 0.4062 | *** | |
| *Scheffersomyces stipitis* | CD4+/CD8+ % of Lymphocytes T | **0.6225** | **0.0058** | 0.0903 | 0.7917 | ** | |
| *Scheffersomyces stipitis* | EM Th % of Regulatory Lymphocytes T | **0.5914** | **0.0097** | -0.0820 | 0.8106 | ** | |
| *Scheffersomyces stipitis* | T cells % of all PBMCs | **0.5865** | **0.0105** | -0.1065 | 0.7554 | * | |
| *Scheffersomyces stipitis* | B cells % of all Lymphocytes | **-0.5303** | **0.0236** | -0.0003 | 0.9993 | * | |
| *Scheffersomyces stipitis* | T cells PD-1+ % of all Lymphocytes | **0.4758** | **0.0459** | -0.0552 | 0.8719 | * | |
| *Scheffersomyces stipitis* | Post-germ center B cells % of memory switched Lymphocytes B | 0.1106 | 0.6621 | **0.8028** | **0.0029** | | ** |
| *Scheffersomyces stipitis* | Switched post-germ center % of memory switched Lymphocytes B | -0.0227 | 0.9288 | **-0.7573** | **0.0069** | | ** |
| *Sporisorium graminicola* | EM Tc % of Cytotoxic Lymphocytes T | 0.1103 | 0.6630 | **-0.8403** | **0.0012** | | ** |
| *Sporisorium graminicola* | Naive Tc % of Cytotoxic Lymphocytes T | 0.0838 | 0.7410 | **0.6777** | **0.0219** | | * |
| *Sporisorium graminicola* | Lymphocytes % of PBMC | -0.0677 | 0.7896 | **-0.6612** | **0.0267** | | * |
| *Sugiyamaella lignohabitans* | Transitional % of Lymphocytes B | **0.8463** | **0.0000** | **-0.6337** | **0.0363** | *** | * |
| *Sugiyamaella lignohabitans* | Mo PD-1+ % of Monocytes | **0.6678** | **0.0025** | 0.4098 | 0.2107 | ** | |
| *Sugiyamaella lignohabitans* | CD4+ % of Lymphocytes T | **0.6241** | **0.0056** | -0.0572 | 0.8673 | ** | |
| *Sugiyamaella lignohabitans* | Tc CD185+ % of Cytotoxic Lymphocytes T | **-0.5366** | **0.0217** | 0.3878 | 0.2386 | * | |
| *Sugiyamaella lignohabitans* | T cells % of all Lymphocytes | **0.5364** | **0.0217** | 0.3108 | 0.3522 | * | |
| *Sugiyamaella lignohabitans* | CD8+ % of Lymphocytes T | **-0.5289** | **0.0240** | 0.1941 | 0.5673 | * | |
| *Sugiyamaella lignohabitans* | Mo CD274+ % of Monocytes | -0.2128 | 0.3966 | **-0.7433** | **0.0088** | | ** |
| *Sugiyamaella lignohabitans* | CD4+/CD8+ % of Lymphocytes T | -0.1859 | 0.4601 | **-0.8148** | **0.0022** | | ** |
| *Sugiyamaella lignohabitans* | B reg transitional % of Lymphocytes B | 0.3664 | 0.1348 | **-0.6197** | **0.0420** | | * |
| *Sugiyamaella lignohabitans* | Mo CD273+/CD274+ % of Monocytes | -0.2656 | 0.2867 | **0.6205** | **0.0417** | | * |
| *Talaromyces rugulosus* | B reg memory % of Lymphocytes B | **0.5201** | **0.0269** | | | * | |
| *Talaromyces rugulosus* | Lymphocytes % of PBMC | **0.4921** | **0.0380** | | | * | |
| *Talaromyces rugulosus* | Late memory % of Lymphocytes B | **-0.4716** | **0.0482** | | | * | |
| *Ustilago maydis* | TD Tc % of Cytotoxic Lymphocytes T | **-0.9066** | **0.0000** | -0.4746 | 0.1402 | *** | |
| *Ustilago maydis* | EM Tc % of Cytotoxic Lymphocytes T | **0.8931** | **0.0000** | 0.0046 | 0.9893 | *** | |
| *Ustilago maydis* | CD4+/CD8+ % of Lymphocytes T | **0.6225** | **0.0058** | -0.0226 | 0.9474 | ** | |
| *Ustilago maydis* | EM Th % of Regulatory Lymphocytes T | **0.5914** | **0.0097** | 0.0187 | 0.9565 | ** | |
| *Ustilago maydis* | T cells % of all PBMCs | **0.5865** | **0.0105** | 0.1076 | 0.7529 | * | |
| *Ustilago maydis* | B cells % of all Lymphocytes | **-0.5303** | **0.0236** | -0.0852 | 0.8033 | * | |
| *Ustilago maydis* | T cells PD-1+ % of all Lymphocytes | **0.4758** | **0.0459** | 0.1164 | 0.7333 | * | |
| *Ustilago maydis* | Post-germ center B cells % of memory switched Lymphocytes B | 0.1106 | 0.6621 | **-0.8518** | **0.0009** | | *** |
| *Ustilago maydis* | Switched post-germ center % of memory switched Lymphocytes B | -0.0227 | 0.9288 | **0.7999** | **0.0031** | | ** |
| *Yarrowia lipolytica* | T cells CD273+/CD274+ % of all Lymphocytes | **0.6049** | **0.0078** | -0.0513 | 0.8810 | ** | |
| *Yarrowia lipolytica* | Plasmacytes % of Lymphocytes B | **0.5902** | **0.0099** | -0.3702 | 0.2624 | ** | |
| *Yarrowia lipolytica* | T cells PD-1+ % of all Lymphocytes | **0.5255** | **0.0251** | -0.2072 | 0.5409 | * | |
| *Yarrowia lipolytica* | T cells PD-1-/CD152- % of all Lymphocytes | **-0.5183** | **0.0276** | 0.1895 | 0.5768 | * | |
| *Yarrowia lipolytica* | Mo CD273+ % of Monocytes | **0.4716** | **0.0482** | -0.0746 | 0.8275 | * | |
| *Yarrowia lipolytica* | CD4+/CD8+ % of Lymphocytes T | 0.1746 | 0.4885 | **-0.8517** | **0.0009** | | *** |
| *Yarrowia lipolytica* | Memory switched % of Lymphocytes B | -0.1843 | 0.4642 | **0.6966** | **0.0172** | | * |

| | | | | | | | |
|---|---|---|---|---|---|---|---|
| ***Zygosaccharomyces rouxii*** | Transitional % of Lymphocytes B | **-0.8463** | **0.0000** | 0.2790 | 0.4061 | *** | |
| ***Zygosaccharomyces rouxii*** | Mo PD-1+ % of Monocytes | **-0.6678** | **0.0025** | -0.5191 | 0.1017 | ** | |
| ***Zygosaccharomyces rouxii*** | CD4+ % of Lymphocytes T | **-0.6241** | **0.0056** | 0.1063 | 0.7558 | ** | |
| ***Zygosaccharomyces rouxii*** | Tc CD185+ % of Cytotoxic Lymphocytes T | **0.5366** | **0.0217** | 0.1606 | 0.6371 | * | |
| ***Zygosaccharomyces rouxii*** | T cells % of all Lymphocytes | **-0.5364** | **0.0217** | 0.0566 | 0.8686 | * | |
| ***Zygosaccharomyces rouxii*** | CD8+ % of Lymphocytes T | **0.5289** | **0.0240** | -0.2121 | 0.5312 | * | |
| ***Zygosaccharomyces rouxii*** | TD Tc % of Cytotoxic Lymphocytes T | 0.0018 | 0.9943 | **-0.6316** | **0.0371** | | * |
| ***Zygotorulaspora mrakii*** | Transitional % of Lymphocytes B | **-0.8132** | **0.0000** | | | *** | |
| ***Zygotorulaspora mrakii*** | CD4+ % of Lymphocytes T | **-0.6486** | **0.0036** | | | ** | |
| ***Zygotorulaspora mrakii*** | Mo PD-1+ % of Monocytes | **-0.6253** | **0.0055** | | | ** | |
| ***Zygotorulaspora mrakii*** | Naive Tc % of Cytotoxic Lymphocytes T | **-0.5260** | **0.0249** | | | * | |
| ***Zygotorulaspora mrakii*** | CD8+ % of Lymphocytes T | **0.4967** | **0.0360** | | | * | |
| ***Zygotorulaspora mrakii*** | T cells % of all Lymphocytes | **-0.4815** | **0.0430** | | | * | |

Statistically significant correlations were marked in **bold**. 0.001***, 0.01**, 0.05*

# SUPPLEMENTARY FIGURES

**Fig S1. Cohort characteristics differentiating analyzed group of patients. (A)** Progression-free survival (PFS) level of clinical beneficiaries (CB, n=37) vs. non-beneficiaries (NB, n=24), and **(B)** responders (R, n=28) vs. non-responders (NR, n=33). **(C)** Serum LDH level of clinical beneficiaries (CB, n=37) vs. non-beneficiaries (NB, n=24), and **(D)** responders (R, n=28) vs. non-responders (NR, n=33). The Wilcoxon test was performed to compare groups, $p<0.05$. The horizontal line shows the median. The lower and upper hinges correspond to the first and third quartiles. The upper and the lower whiskers extend from the hinge to the largest and smallest value, respectively, no further than 1.5 * IQR from the hinge (where IQR is the inter-quartile range, or distance between the first and third quartiles). Dots represent individual samples. Only samples from the BT group were analyzed.

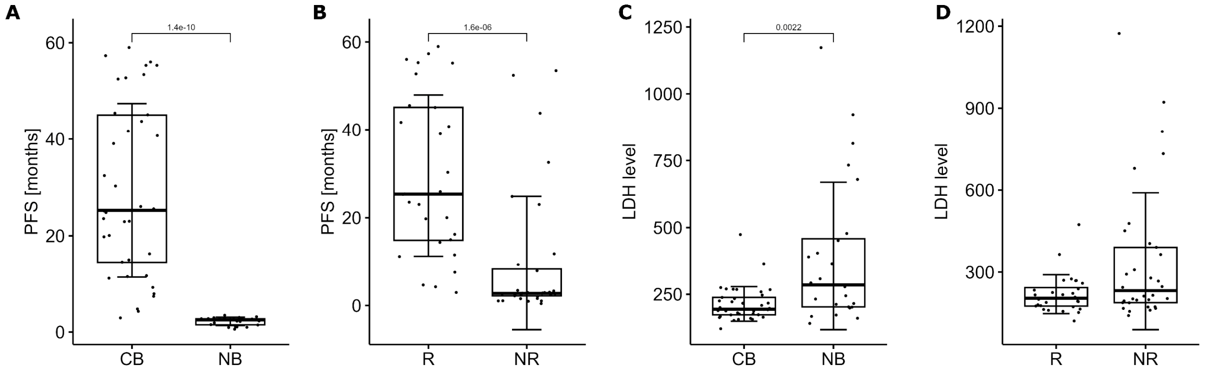

**Fig S2. Relationships between cohort characteristics and levels of lymphocytes circulating in the blood of patients.** Pearson correlation was performed, $p<0.05$. Linear regression (solid line) with 95% confidence interval (grey band) of the fit is shown. Dots represent individual samples. Only samples from the BT group were analyzed.

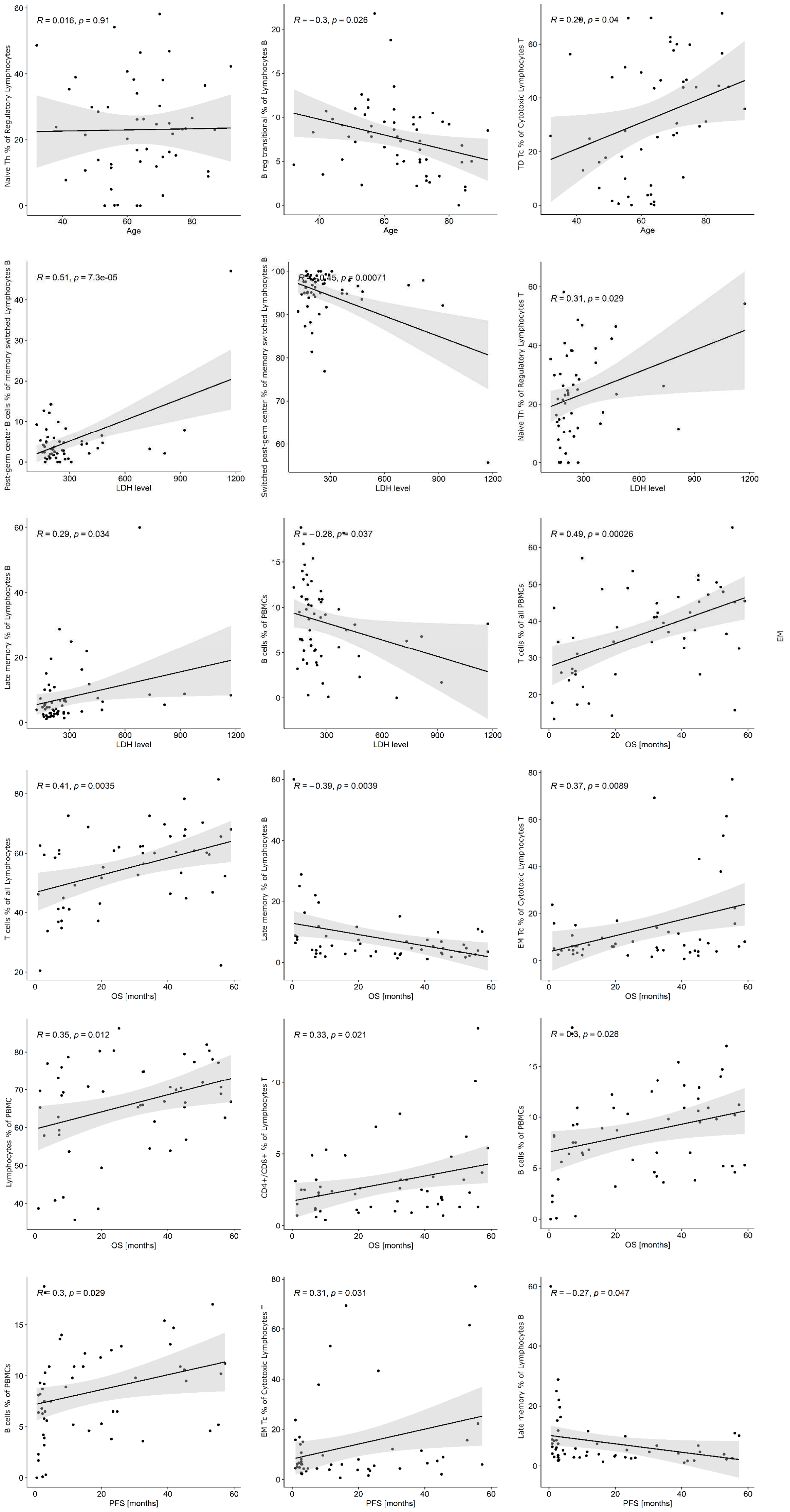

**Fig S3. Differences in lymphocyte levels depending on division according to selected patient differentiation criteria such as age group (<60 (n=19) vs. ≥60 (n=42)), LDH level (low (n=49) vs. high (n=12)), overall survival (OS, ≤12 (n=21) vs. >12 (n=39)), progression-free survival (PFS, ≤12 (n=32), >12 (n=28)).** The Wilcoxon test was performed to compare groups, $p<0.05$. The horizontal line shows the median. The lower and upper hinges correspond to the first and third quartiles. The upper and the lower whiskers extend from the hinge to the largest and smallest value, respectively, no further than 1.5 * IQR from the hinge (where IQR is the inter-quartile range, or distance between the first and third quartiles). Dots represent individual samples. Only samples from the BT group were analyzed.

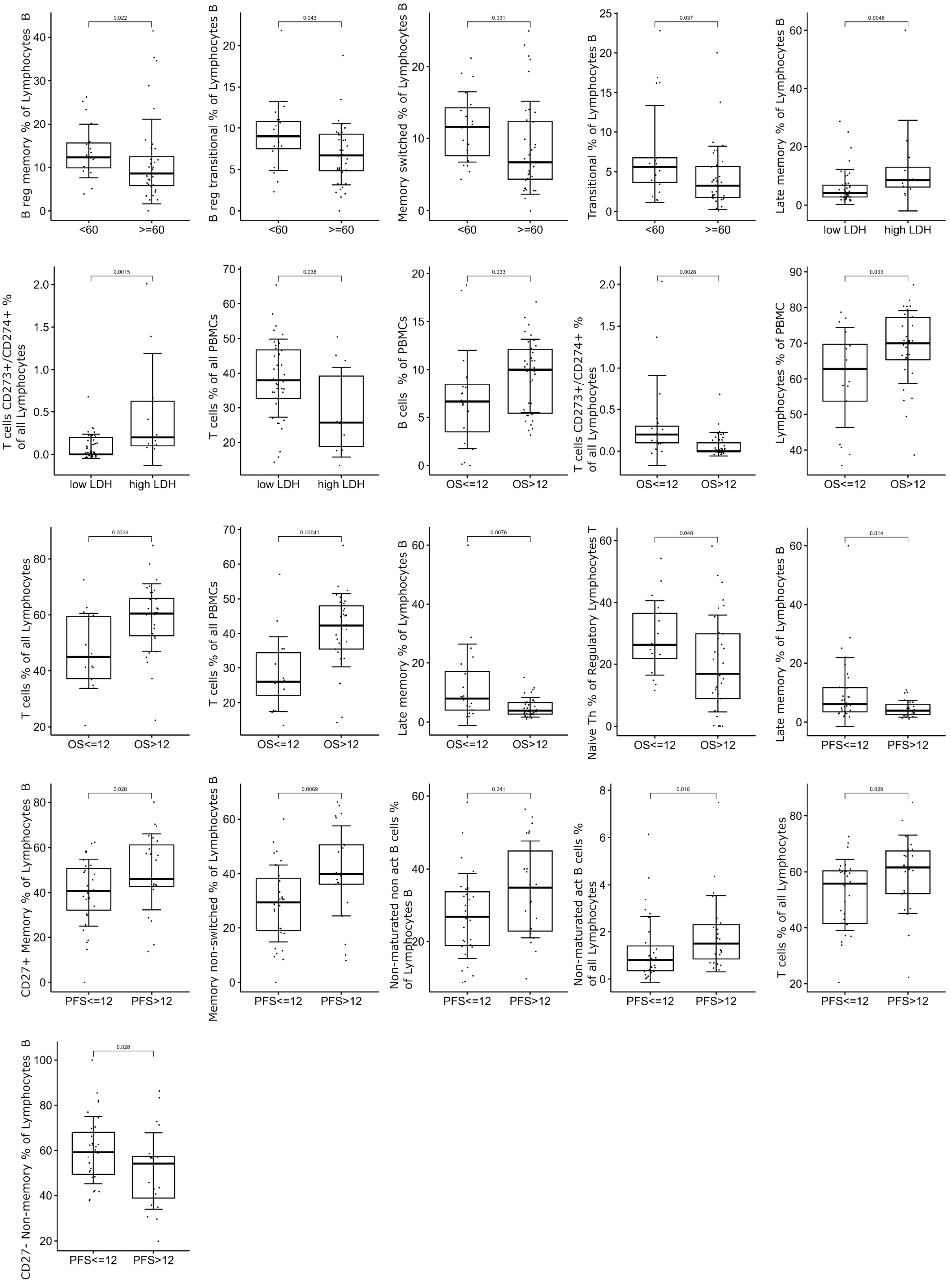

**Fig S4. Differences in lymphocyte levels depending on the time of treatment (BT (n=61) vs. T3 (n=37)).** The Wilcoxon test was performed to compare groups, $p<0.05$. The horizontal line shows the median. The lower and upper hinges correspond to the first and third quartiles. The upper and the lower whiskers extend from the hinge to the largest and smallest value, respectively, no further than 1.5 * IQR from the hinge (where IQR is the inter-quartile range, or distance between the first and third quartiles). Dots represent individual samples. Samples from the BT and T3 groups were analyzed.

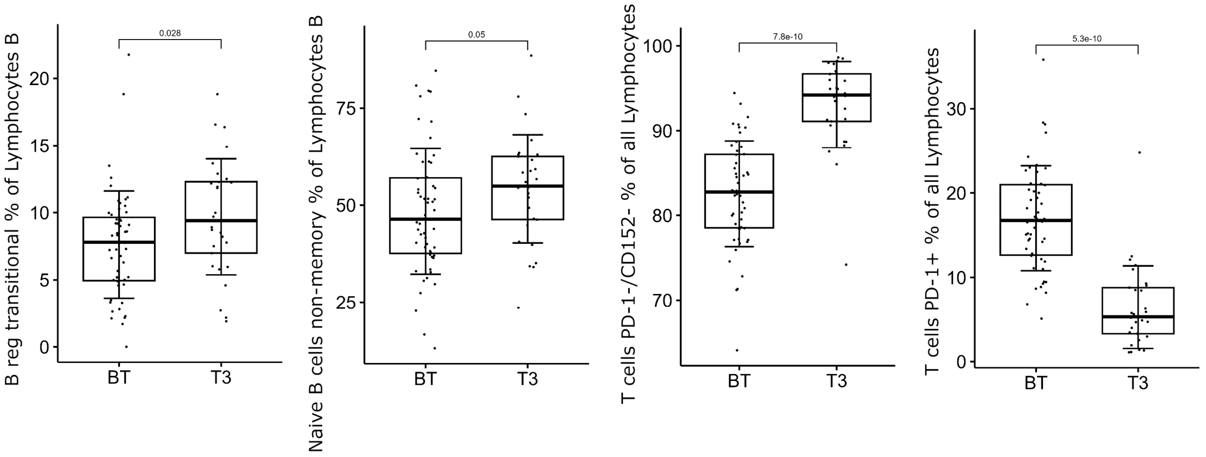

**Fig S5. Differences in lymphocyte levels depending on the clinical response based on data from the BT group (CB (n=37) vs. NB (n=24), and R (n=28) vs. NR (n=33)).** The Wilcoxon test was performed to compare groups, $p<0.05$. The horizontal line shows the median. The lower and upper hinges correspond to the first and third quartiles. The upper and the lower whiskers extend from the hinge to the largest and smallest value, respectively, no further than 1.5 * IQR from the hinge (where IQR is the inter-quartile range, or distance between the first and third quartiles). Dots represent individual samples.

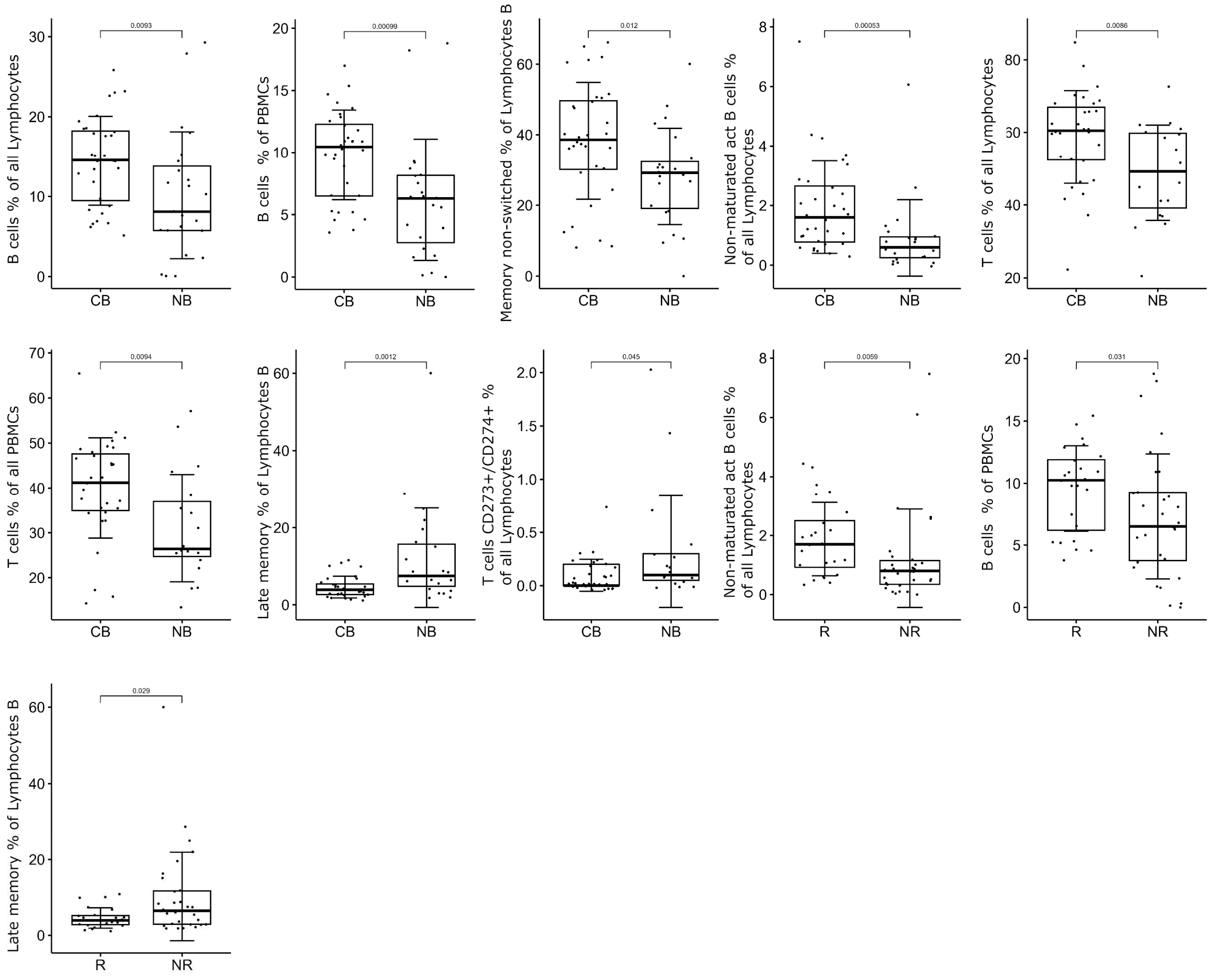

**Fig S6. Differences in lymphocyte levels depending on the clinical response based on data from the T3 group (CB (n=29) vs. NB (n=8), and R (n=22) vs. NR (n=15)).** The Wilcoxon test was performed to compare groups, $p<0.05$. The horizontal line shows the median. The lower and upper hinges correspond to the first and third quartiles. The upper and the lower whiskers extend from the hinge to the largest and smallest value, respectively, no further than 1.5 * IQR from the hinge (where IQR is the inter-quartile range, or distance between the first and third quartiles). Dots represent individual samples.

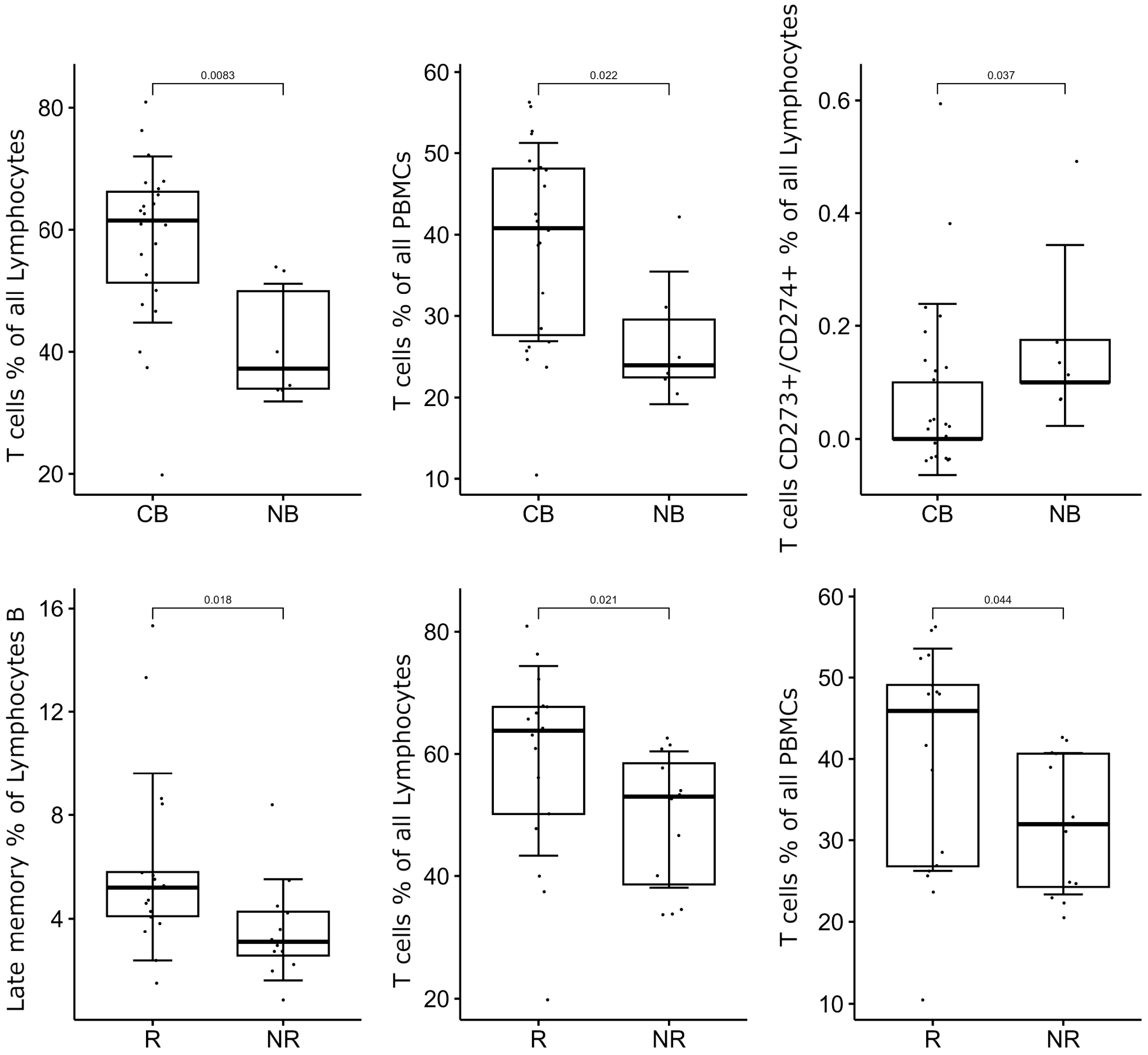

**Fig S7. Mycobiota structure of samples considering the time of treatment (BT (n=53) vs. T3 (n=33)) at different taxonomical levels, averaged by group. (A)** Genus **(B)** Family **(C)** Order **(D)** Class **(E)** Phylum. Samples without fungi were filtered out.

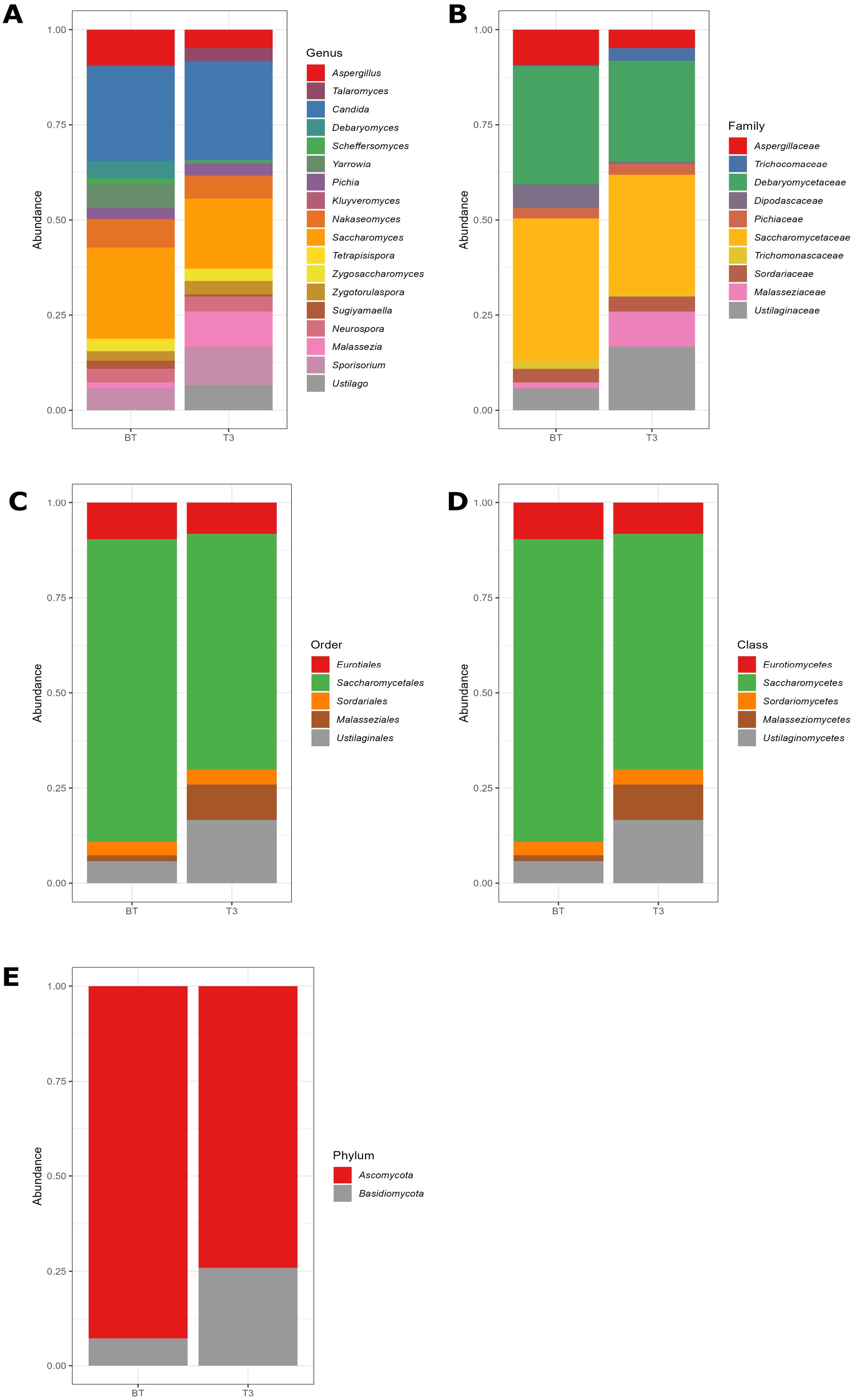

**Fig S8. Mycobiota structure of samples considering the time of treatment (BT (n=53) vs. T3 (n=33)) at different taxonomical levels, relative abundance.** **(A)** Species **(B)** Genus **(C)** Family **(D)** Order **(E)** Class **(F)** Phylum. Samples without fungi were filtered out. Bars represent individual samples.

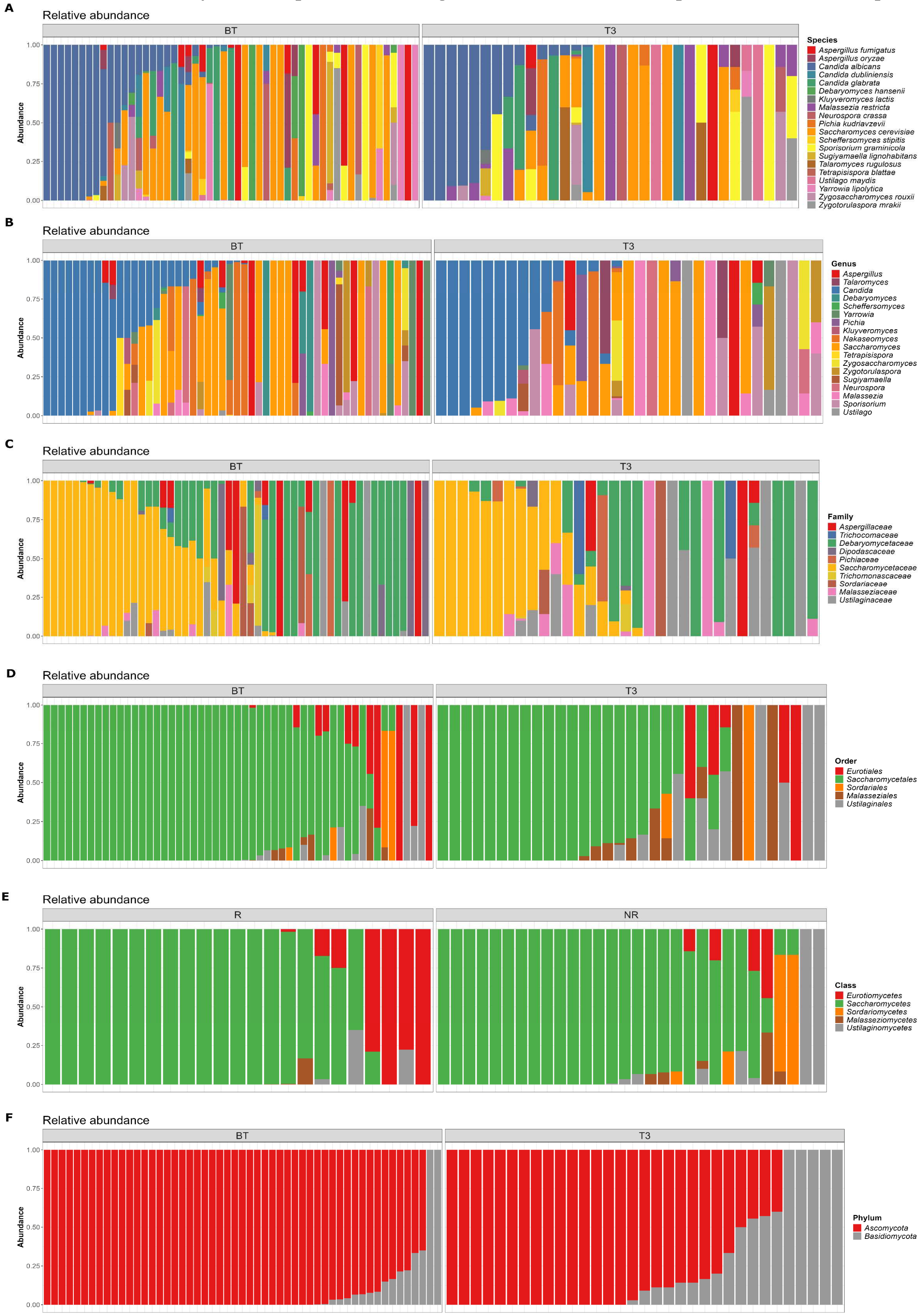

**Fig S9. Mycobiota structure of samples considering the response to the anti-PD-1 therapy (CB (n=30) vs. NB (n=23)) at different taxonomical levels, averaged by group. (A)** Genus **(B)** Family **(C)** Order **(D)** Class **(E)** Phylum. Samples without fungi were filtered out. Bars represent individual samples.

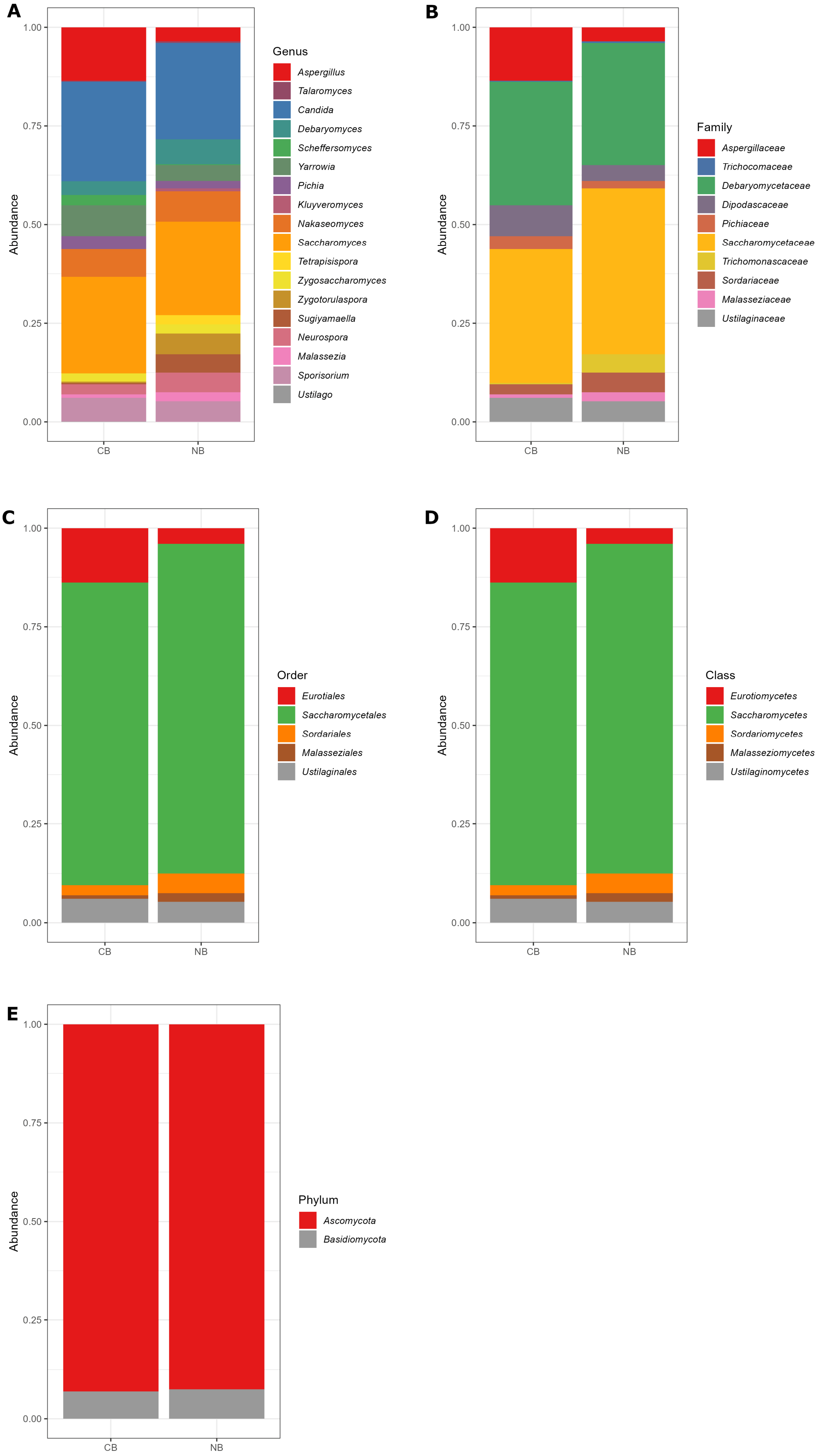

**Fig S10. Mycobiota structure of samples considering the response to the anti-PD-1 therapy (CB (n=30) vs. NB (n=23)), relative abundance.** **(A)** Species **(B)** Genus **(C)** Family **(D)** Order **(E)** Class **(F)** Phylum. Samples without fungi were filtered out. Bars represent individual samples.

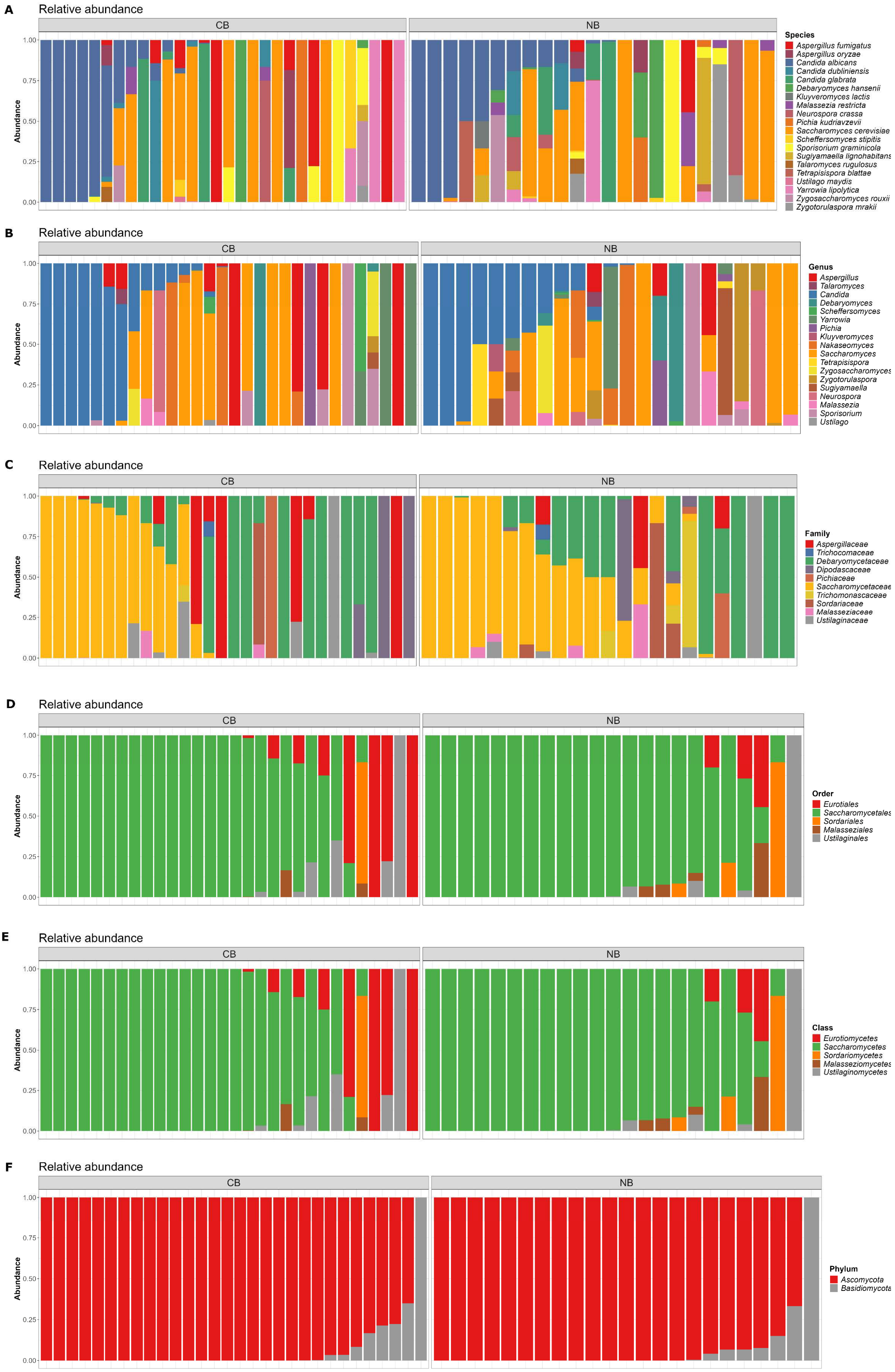

**Fig S11. Mycobiota structure of samples considering the response to the anti-PD-1 therapy (R (n=23) vs. NR (n=30)) at different taxonomical levels, averaged by group.** (**A**) Genus (**B**) Family (**C**) Order (**D**) Class (**E**) Phylum. Samples without fungi were filtered out. Bars represent individual samples.

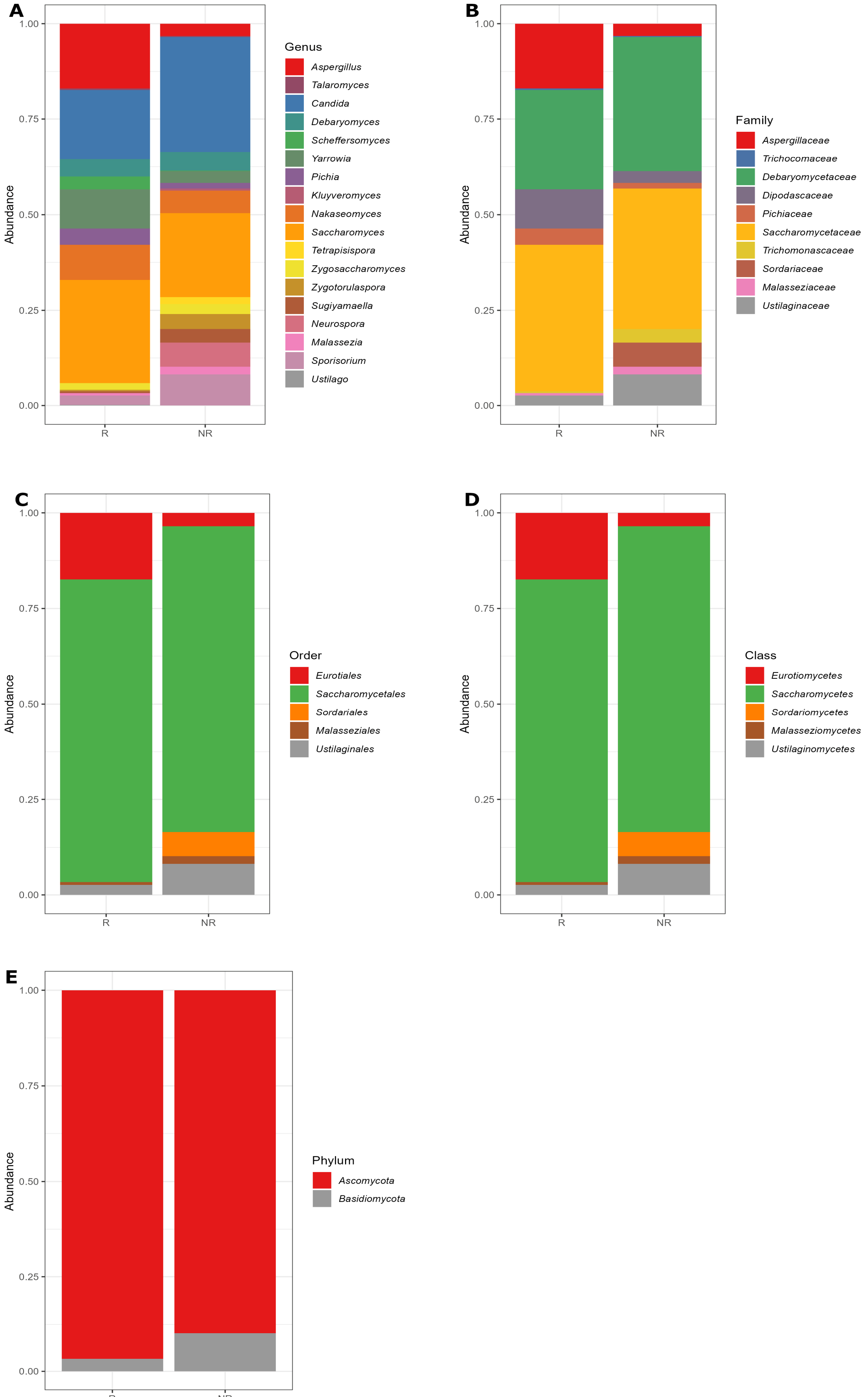

**Fig S12. Mycobiota structure of samples considering the response to the anti-PD-1 therapy (R (n=23) vs. NR (n=30)) at different taxonomical levels, relative abundance. (A)** Species **(B)** Genus **(C)** Family **(D)** Order **(E)** Class **(F)** Phylum. Samples without fungi were filtered out. Bars represent individual samples.

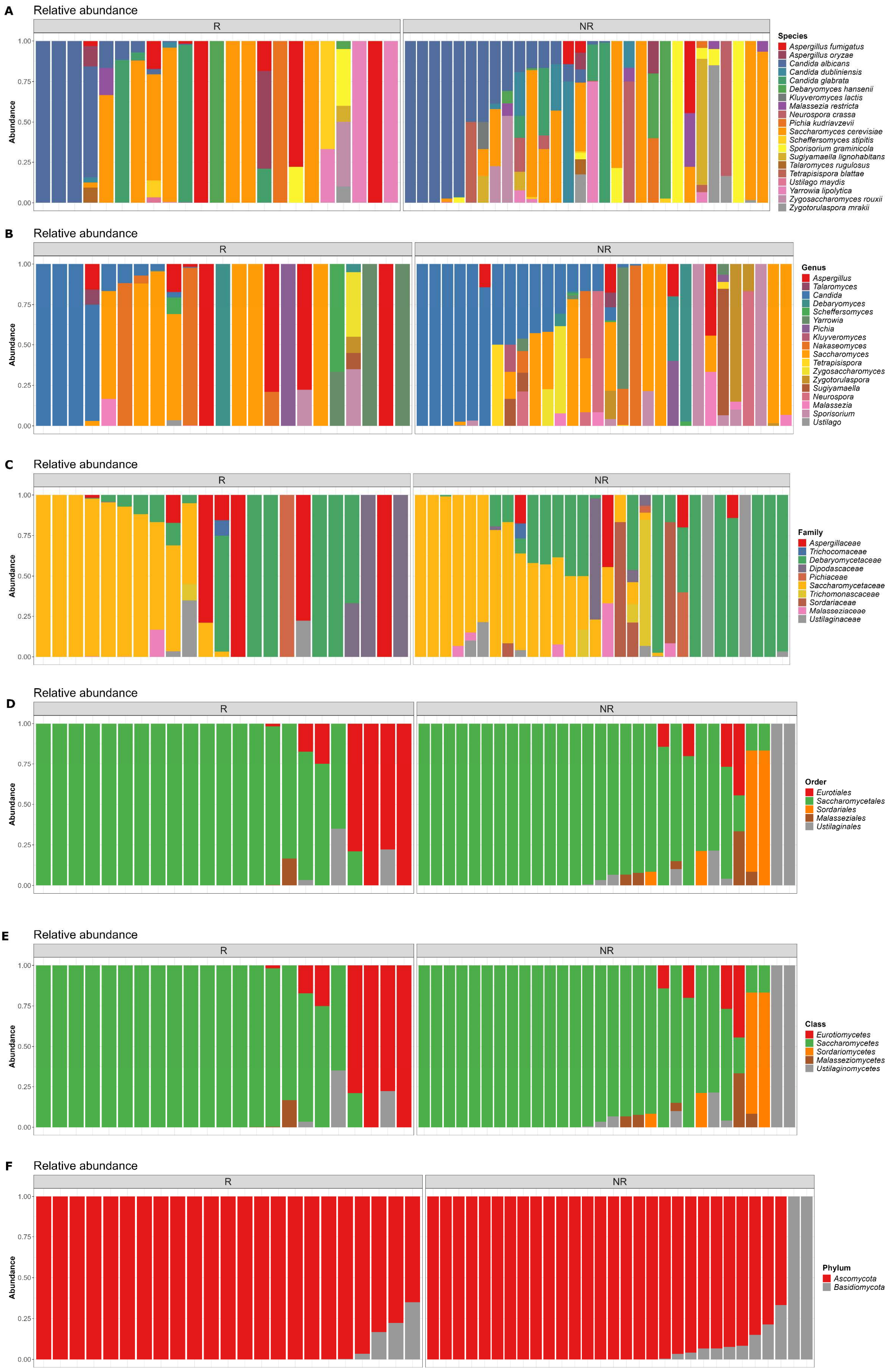

**Fig S13. Relationships between gut fungi and lymphocytes regarding the response to anti-PD-1 therapy, R and NR.** Heatmap for **(A)** R, n=23, and **(B)** NR, n=30. Graphs of correlations for **(C)** R, n=23, and **(D)** NR, n=30. Only statistically significant correlations are shown on graphs. Pearson correlation was performed, p<0.05. 0.001***, 0.01**, 0.05*.

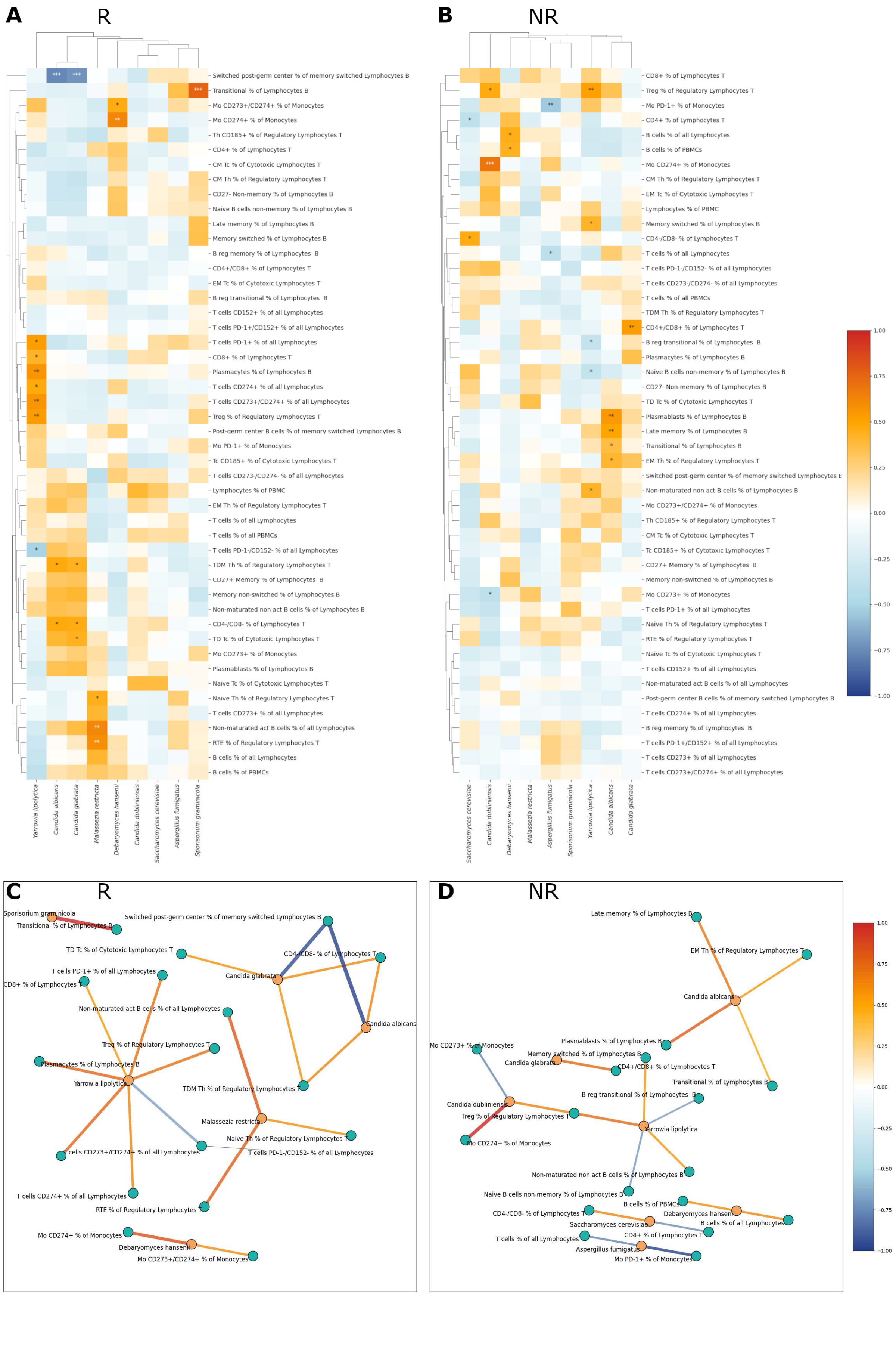

**Fig S14. Relationships between changes in gut fungi count and changes in the number of lymphocytes regarding the response to anti-PD-1 therapy, R and NR.** Heatmap for **(A)** responders (R, n=22) and **(B)** non-responders (NR, n=14). Graphs of correlations for **(C)** R (n=22) and **(D)** NR (n=14). Paired samples from the BT (n=36) and T3 (n=36) groups were analyzed. Only statistically significant correlations are shown on graphs. Pearson correlation was performed, p<0.05. 0.001***, 0.01**, 0.05*.

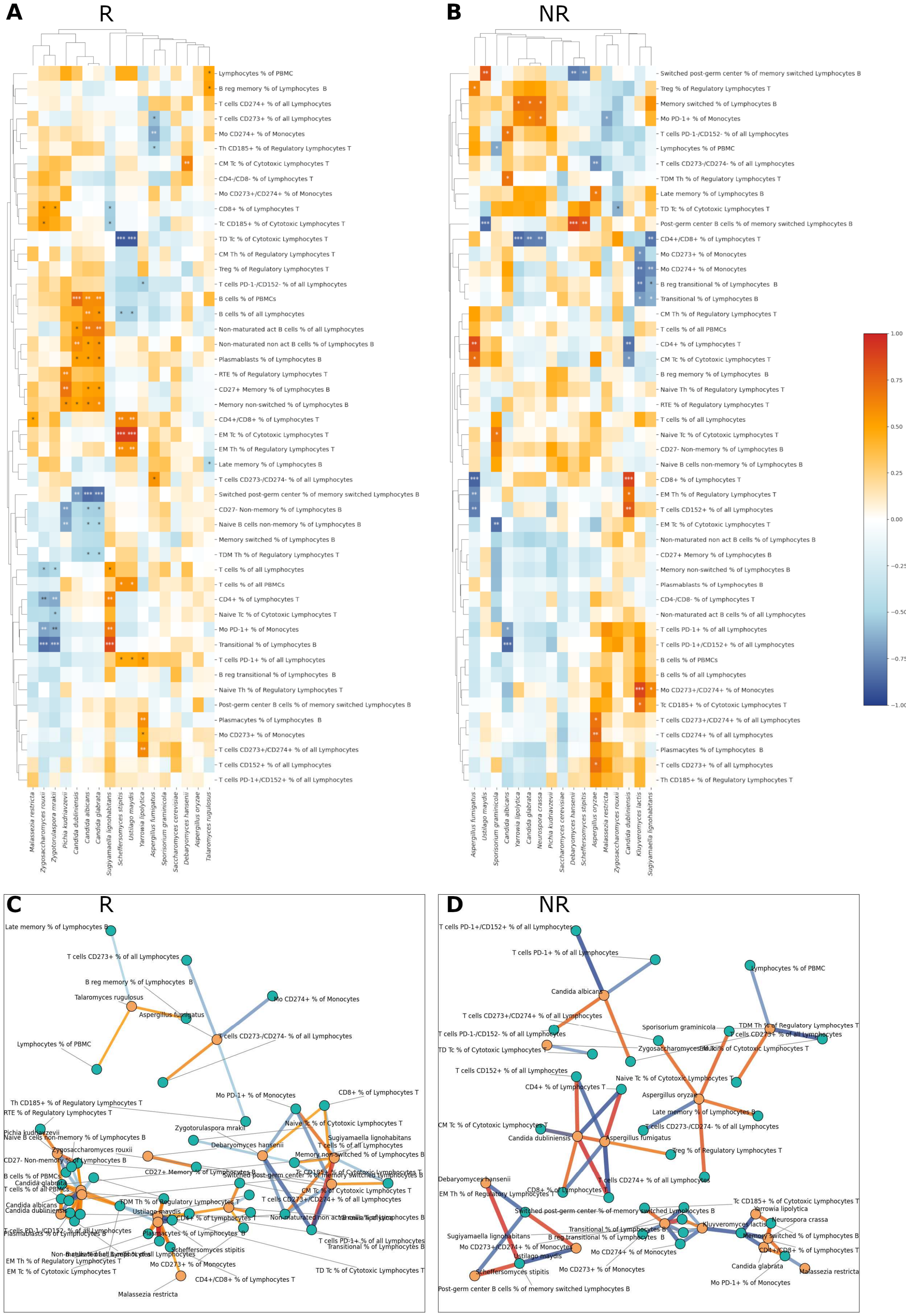

Supplement: Supplementary file 2 — Supplementary file2 (PDF 6831 KB) [file 262_2024_3918_MOESM2_ESM.pdf]
